# Associations between plant-based dietary patterns and risks of type 2 diabetes, cardiovascular disease, cancer, and mortality – a systematic review and meta-analysis

**DOI:** 10.1186/s12937-023-00877-2

**Published:** 2023-10-04

**Authors:** Yeli Wang, Binkai Liu, Han Han, Yang Hu, Lu Zhu, Eric B. Rimm, Frank B. Hu, Qi Sun

**Affiliations:** 1 Department of Nutrition, Harvard T.H. Chan School of Public Health, 677 Huntington Avenue, Boston, MA 02115 USA; 2 CAS Key Laboratory of Nutrition, Metabolism and Food Safety, Shanghai Institute of Nutrition and Health, University of Chinese Academy of Sciences, Chinese Academy of Sciences, Shanghai, China; 3 Department of Epidemiology, Harvard T.H. Chan School of Public Health, Boston, MA USA; 4 Channing Division of Network Medicine, Department of Medicine, Brigham and Women’s Hospital, Harvard Medical School, Boston, MA USA

**Keywords:** Systematic review, Meta-analysis, Plant-based dietary patterns, Type 2 diabetes, Cardiovascular disease, Cancer, Mortality

## Abstract

**Background:**

Plant-based dietary patterns are gaining more attention due to their potential in reducing the risk of developing major chronic diseases, including type 2 diabetes (T2D), cardiovascular disease (CVD), cancer, and mortality, while an up-to-date comprehensive quantitative review is lacking. This study aimed to summarize the existing prospective observational evidence on associations between adherence to plant-based dietary patterns and chronic disease outcomes.

**Methods:**

We conducted a systematic review and meta-analysis of evidence across prospective observational studies. The data sources used were PubMed and MEDLINE, Embase, Web of Science, and screening of references. We included all prospective observational studies that evaluated the association between adherence to plant-based dietary patterns and incidence of T2D, CVD, cancer, and mortality among adults (≥ 18 years).

**Results:**

A total of 76 publications were identified, including 2,230,443 participants with 60,718 cases of incident T2D, 157,335 CVD cases, 57,759 cancer cases, and 174,435 deaths. An inverse association was observed between higher adherence to a plant-based dietary pattern and risks of T2D (RR, 0.82 [95% CI: 0.77–0.86]), CVD (0.90 [0.85–0.94]), cancer (0.88 [0.84–0.92]), and all-cause mortality (0.84 [0.78–0.92]) with moderate to high heterogeneity across studies (*I*^2^ ranged: 30.2–95.4%). The inverse associations with T2D, CVD and cancer were strengthened when healthy plant-based foods, such as vegetables, fruits, whole grains, and legumes, were emphasized in the definition of plant-based dietary patterns (T2D: 0.79 [0.72–0.87]; CVD: 0.85 [0.80–0.92]; cancer: 0.86 [0.80–0.92]; *I*^2^ ranged: 53.1–84.1%). Association for mortality was largely similar when the analyses were restricted to healthy plant-based diets (0.86 [0.80–0.92], *I*^2^ = 91.9%). In contrast, unhealthy plant-based diets were positively associated with these disease outcomes. Among four studies that examined changes in dietary patterns, increased adherence to plant-based dietary patterns was associated with a significantly reduced risk of T2D (0.83 [0.71–0.96]; *I*^2^ = 71.5%) and a marginally lower risk of mortality (0.95 [0.91–1.00]; *I*^2^ = 0%).

**Conclusions:**

Better adherence to plant-based dietary patterns, especially those emphasizing healthy plant-based foods, is beneficial for lowering the risks of major chronic conditions, including T2D, CVD, cancer, as well as premature deaths.

**Registration of review protocol:**

This review was registered at the PROSPERO International Prospective Register of Systematic Reviews (https://www.crd.york.ac.uk/PROSPERO/) with the registration number CRD42022290202.

**Supplementary Information:**

The online version contains supplementary material available at 10.1186/s12937-023-00877-2.

## Introduction

According to the World Health Organization, type 2 diabetes (T2D), cardiovascular disease (CVD), and cancer account for nearly one of every two deaths globally – close to 30 million people in 2020 [1–3]. These diseases had significant clinical and public health implications by undermining health, shortening life expectancy, and causing enormous suffering, disability, and economic costs [4]. Habitual diet is known to play a critical role in modifying risks of these diseases. Plant-based dietary patterns that emphasize foods derived from plant sources and de-emphasize consumption of animal products have gained significant attention in recent years. They have been associated with lower risks of CVD, cancer, and T2D in prospective human studies [5–7], and thus have the potential to prevent and manage these diseases.

Although a few systematic reviews and meta-analyses have examined associations between long-term vegetarian versus non-vegetarian dietary patterns and risks of CVD [6, 8] and cancer [7, 9–11], much less has been examined systematically on whether plant-based diets that do not necessarily exclude animal products also lower risks of these chronic conditions. In comparison with vegan or vegetarian diets, plant-based diets may be more feasible for the general population to adopt. Moreover, more studies have been published since the last meta-analyses of plant-based diets in relation to T2D [12–20] and mortality [21–28]. Furthermore, no meta-analysis has quantitatively evaluated the impact of changes in plant-based dietary patterns on risks of T2D and mortality.

To summarize the current evidence regarding plant-based diets in relation to human health, we conducted an updated systematic review and meta-analysis of prospective studies to assess the association of adherence to plant-based dietary patterns and changes in plant-based dietary patterns with risks of major chronic diseases, including T2D, CVD, and cancer, as well as mortality. We hypothesized that higher adherence to plant-based dietary patterns is associated with lower risks of T2D, CVD, cancer, and mortality.

## Methods

### Data sources and searches

We systematically searched three databases, including PubMed and MEDLINE, Embase, and Web of Science through June 29, 2023. The search initially started in June 2021, and then updated in June 2023. Reference lists of prior systematic reviews were also checked to identify relevant original studies that were not found in three databases. Search terms included “plant-based diet”, “vegetarian”, “vegan”, “type 2 diabetes”, “cardiovascular disease”, “stroke”, “coronary artery disease”, “myocardial infarction”, “angina pectoris”, “cerebrovascular disorders”, “cancer”, and “mortality”. MESH and Emtree terms with explosion of narrower terms were used to broaden search results. The detailed search strategy for each database and the number of records retrieved was presented in Supplemental Table S1. We followed the recommended standards in the Meta-analysis Of Observational Studies in Epidemiology (MOOSE) [29] guideline. To report the studies included, we used the Preferred Reporting Items for Systematic reviews and Meta-Analyses (PRISMA) [30].

### Study selection

The current analysis considered prospective studies (cohort studies, case-cohort studies, or nested case-control studies) that assessed associations between adherence to a priori-defined plant-based dietary patterns and the incidence of T2D, CVD, cancer, and mortality among adults (aged ≥ 18 years). Other types of studies (e.g., cross-sectional studies, reviews, commentaries, meeting abstracts), studies using a posteriori approach (e.g., principal component analysis, factor analysis) to derive dietary patterns, or studies reported outcomes other than T2D (e.g., type 1 diabetes, gestational diabetes), CVD, cancer, and mortality were excluded. The detailed inclusion and exclusion criteria were presented in Supplemental Table S2.

The primary exposure of interest was adherence to plant-based dietary patterns, defined as higher consumption of plant-based foods and lower consumption or exclusion of animal-based foods, or changes in plant-based dietary patterns. By this definition, vegetarian or vegan dietary patterns were also considered as plant-based dietary patterns. In studies that classified plant-based dietary patterns using overall, healthful or unhealthful plant-based dietary indices (PDI, hPDI, uPDI), the association for overall plant-based dietary index was included in the pooled risk estimate. We also summarized studies that explicitly examined healthful or unhealthful plant-based dietary patterns that further de-emphasize the intake of unhealthy plant-based foods, such as refined grains, starchy vegetables (e.g., white potatoes), or sugar (e.g., sweets, desserts, or sugar-sweetened beverages).

For studies that used dietary indices, we used the risk estimate that compared the highest with lowest quantiles, which represent the best (highest quantile) and poorest (lowest quantile) adherence to the plant-based dietary pattern. For studies that compared distinct dietary patterns (vegetarian, semi-vegetarian, non-vegetarian), we considered the study estimates comparing diets that are most restrictive of animal-based foods (vegan or vegetarian) with the least restrictive, such as omnivorous diets.

The primary outcomes were T2D, any CVD, any cancer, and all-cause mortality. In the secondary analysis, we also examined subtypes of CVD (coronary heart disease [CHD] including ischemic heart disease and myocardial infarction, stroke, and heart failure), cancer (e.g., breast cancer, colorectal cancer, lung cancer, and prostate cancer), and mortality (cancer mortality, and CVD mortality).

### Data extraction and assessment of quality

Data from included studies were independently extracted by three authors (Y.W., B.L., and H.H.). Any discrepancies were evaluated by another author (Q.S.) and discussed among four authors. The following information was extracted from each study: author, year, study name, region, disease outcome, number of participants and cases of incident T2D, CVD, cancer, or deaths, mean age, mean body mass index (BMI; calculated as weight in kilograms divided by height in meters squared), sex (percentage of men), follow-up duration, dietary assessment method, method of scoring adherence to plant-based diet, outcome ascertainment, and covariates that were adjusted for in the statistical models. The study quality was assessed using the Quality Assessment Tool for Observational Cohort and Cross-Sectional Studies available on the National Heart, Lung, and Blood Institute website (https://www.nhlbi.nih.gov/health-topics/study-quality-assessment-tools). This tool assigns a maximum score of 14 to each study where 0 indicates that the study does not meet any of the criteria for quality assessments and 14 indicates that the study meets all 14 criteria based on potential areas of bias such as duration of follow-up, assessment of exposure and outcomes, and rates of loss to follow-up. Studies identified with high risk of bias (quality score < 10) were excluded in a sensitivity analysis.

### Data synthesis and analysis

We calculated a pooled relative risk (RR) using random-effects meta-analysis of hazard ratio, relative risk, rate ratio, or odds ratio from each study. The risk estimate from the statistical model with the highest degree of adjustment was included in the meta-analysis. For studies that only reported continuous plant-based dietary indices (per 25 percentile increment, per 5-unit increment, per 10-unit increment) [18, 21, 31–36], we converted the RRs to those comparing the extreme quintiles of dietary scores (*riskconv* package from STATA) [37, 38].

Statistical heterogeneity was assessed with the Cochran Q-test (*P* < 0.10) and *I*^2^ statistic. We performed subgroup analyses to characterize potential sources of heterogeneity, including analyses stratified by age, sex, BMI (< 25 vs. ≥25 kg/m^2^), global region (North America, Europe, Asia/Australia), definitions of plant-based diets (e.g., vegetarian or vegan diet vs. plant-based dietary scores), and follow-up duration (< 15 vs. ≥15 years). In addition, we performed meta-regression analyses using age, sex, BMI, and follow-up duration to explore potential sources of heterogeneity. Sensitivity analyses included those using inverse-variance fixed-effects meta-analysis as well as sequentially excluding individual studies to examine the effect on the overall risk estimate. Publication bias was assessed visually with funnel plots and with the Egger or Begg-Mazumdar regression tests [39]. All analyses were conducted with STATA, version 17.0 (STATA Corp). All *P* values were from 2-sided tests, and the results were statistically significant with a *P* < 0.05 unless stated otherwise.

## Results

### Included studies and baseline characteristics

**Fig. 1:**
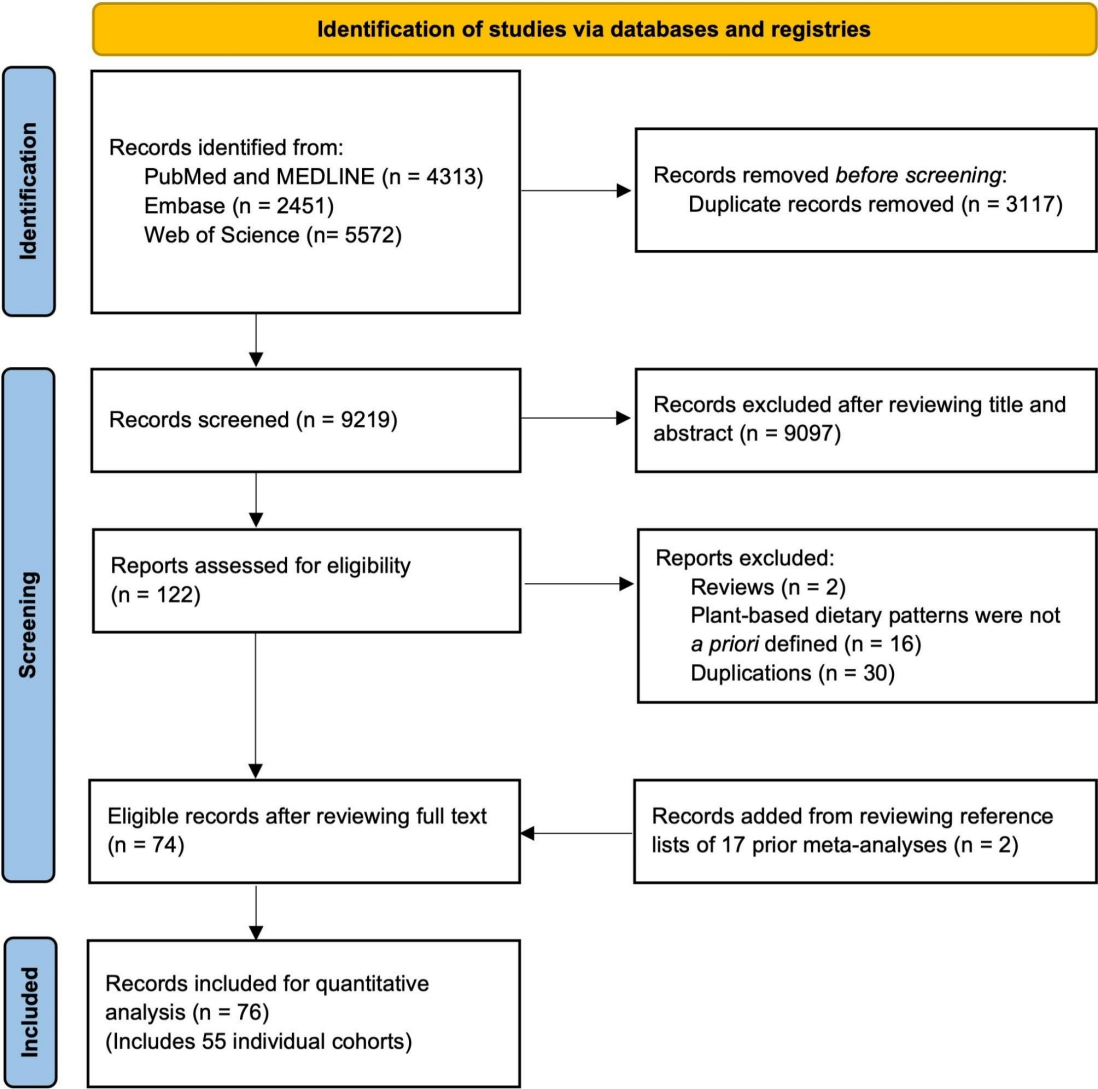
PRISMA Flowchart of Prospective Observational Studies of Plant-Based Dietary Patterns and Incident Type 2 Diabetes, Cardiovascular Disease, Cancer, and Mortality

The PRISMA flow diagram of search and screening results from three databases were presented in Fig. 1. PRISMA checklist was presented in the Additional File 1. Our initial search identified 12,336 citations, of which, after excluding ineligible studies due to various reasons, 76 publications from 55 individual cohorts were included in the current analysis (Fig. 1). Of those, 17 publications examined T2D, 16 for CVD, 22 for cancer, and 27 for mortality. The characteristics of studies included in the meta-analysis were shown in Supplemental Table S3. The meta-analysis included 2,230,443 participants and 60,718 T2D cases, 157,335 CVD cases, 57,759 cancer cases, and 174,435 deaths. Studies had a follow-up duration between 2 and 36 years. All studies used a prospective cohort design except for one study that used a nested case-control study design [40]. The mean age ranged from 24.9 to 86.9 years, and the mean BMI ranged between 20.3 and 30.0 kg/m^2^ at baseline. Majority of studies used food frequency questionnaires (FFQs) to assess diet, while six studies used 24-hour recalls. Twenty-nine publications used repeated measurements of dietary intake, forty-seven publications used one-time dietary measurement at study baseline. A total of 49 publications characterized adherence to plant-based dietary patterns using pre-specified plant-based dietary indices (e.g., overall plant-based dietary index, healthful plant-based dietary index, pro plant-based dietary pattern, provegetarian food pattern, EAT-Lancet diet, Portfolio diet), and 27 publications compared vegetarian or vegan dietary patterns with those who consumed animal-based foods. Six studies examined the change of dietary patterns. Thirty-five publications examined healthful plant-based dietary index, and 29 publications examined unhealthful plant-based index. The study quality ranged between 6 and 14 (low to high), where most publications had high quality (*n* = 67, 88.2%) indicated by a score of 10 or higher.

### Plant-based diet and risk of T2D, CVD, cancer, and mortality

**Fig. 2:**
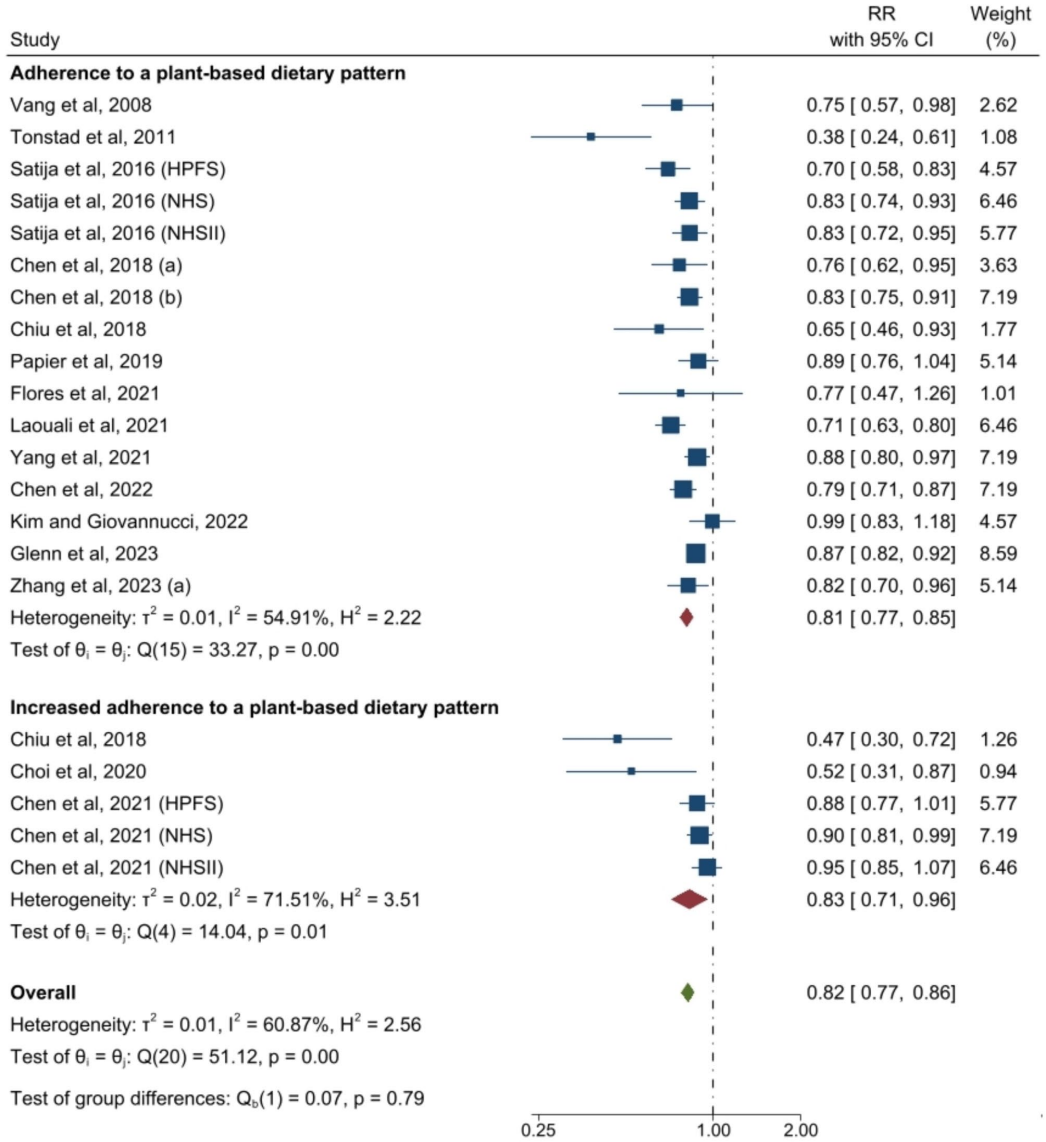
Forest Plot of Studies Examining the Association Between Plant-Based Dietary Patterns and Risks of Type 2 Diabetes using Random-Effects Meta-Analysis **Abbreviations**: HPFS, Health Professionals Follow-up Study; NHS, Nurses’ Health Study; NHSII, Nurses’ Health Study II

**Fig. 3:**
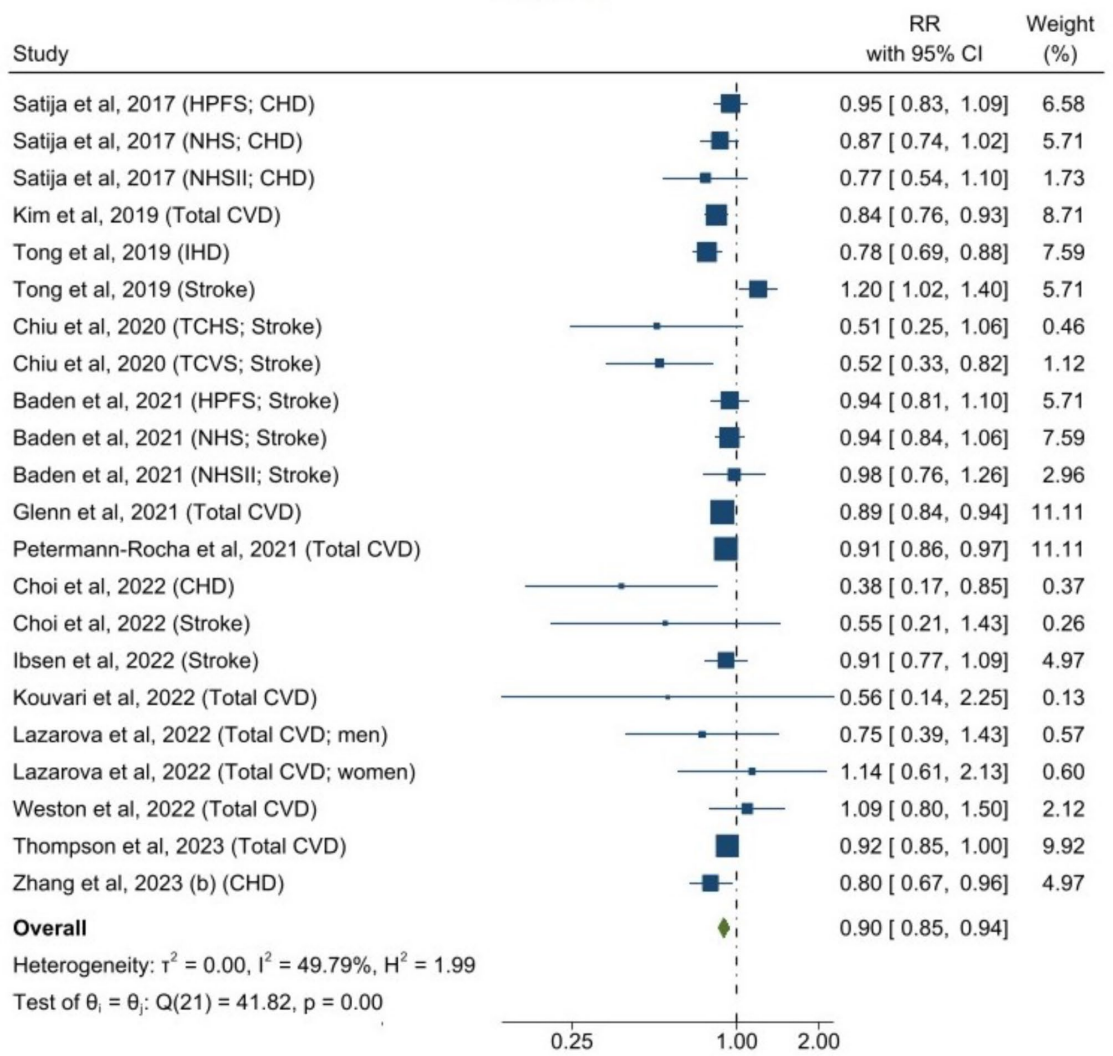
Forest Plot of Studies Examining the Association Between Plant-Based Dietary Patterns and Risks of Cardiovascular Disease using Random-Effects Meta-Analysis **Abbreviations**: CHD, coronary heart disease; CVD, cardiovascular disease; IHD, ischemic heart disease; TCHS, The Tzu Chi Health Study; TCVS, The Tzu Chi Vegetarian Study; HPFS, Health Professionals Follow-up Study; NHS, Nurses’ Health Study; NHSII, Nurses’ Health Study II

**Fig. 4:**
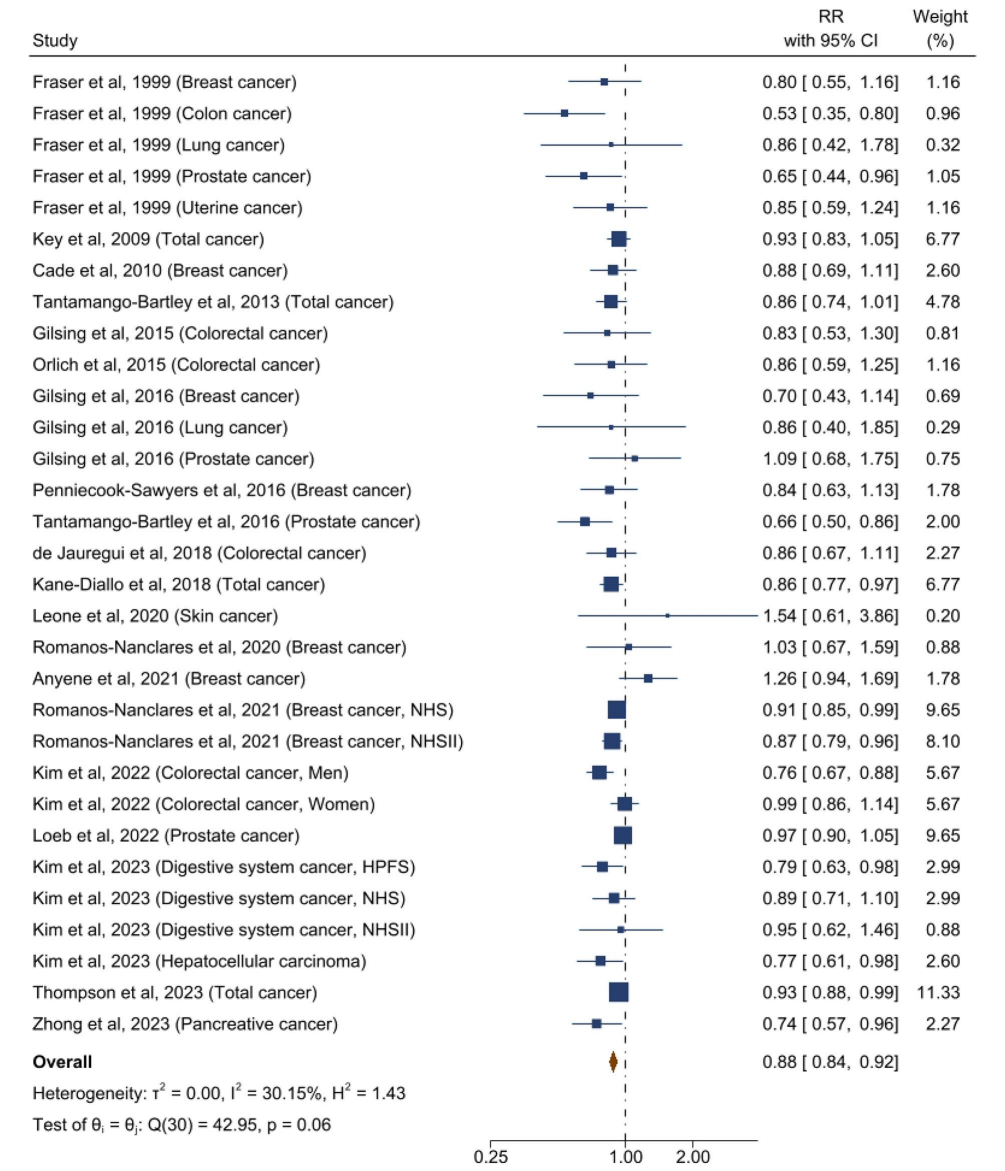
Forest Plot of Studies Examining the Association Between Plant-Based Dietary Patterns and Risks of Cancer using Random-Effects Meta-Analysis **Abbreviations**: NHS, Nurses’ Health Study; NHSII, Nurses’ Health Study II; HPFS, Health Professionals Follow-up Study

**Fig. 5:**
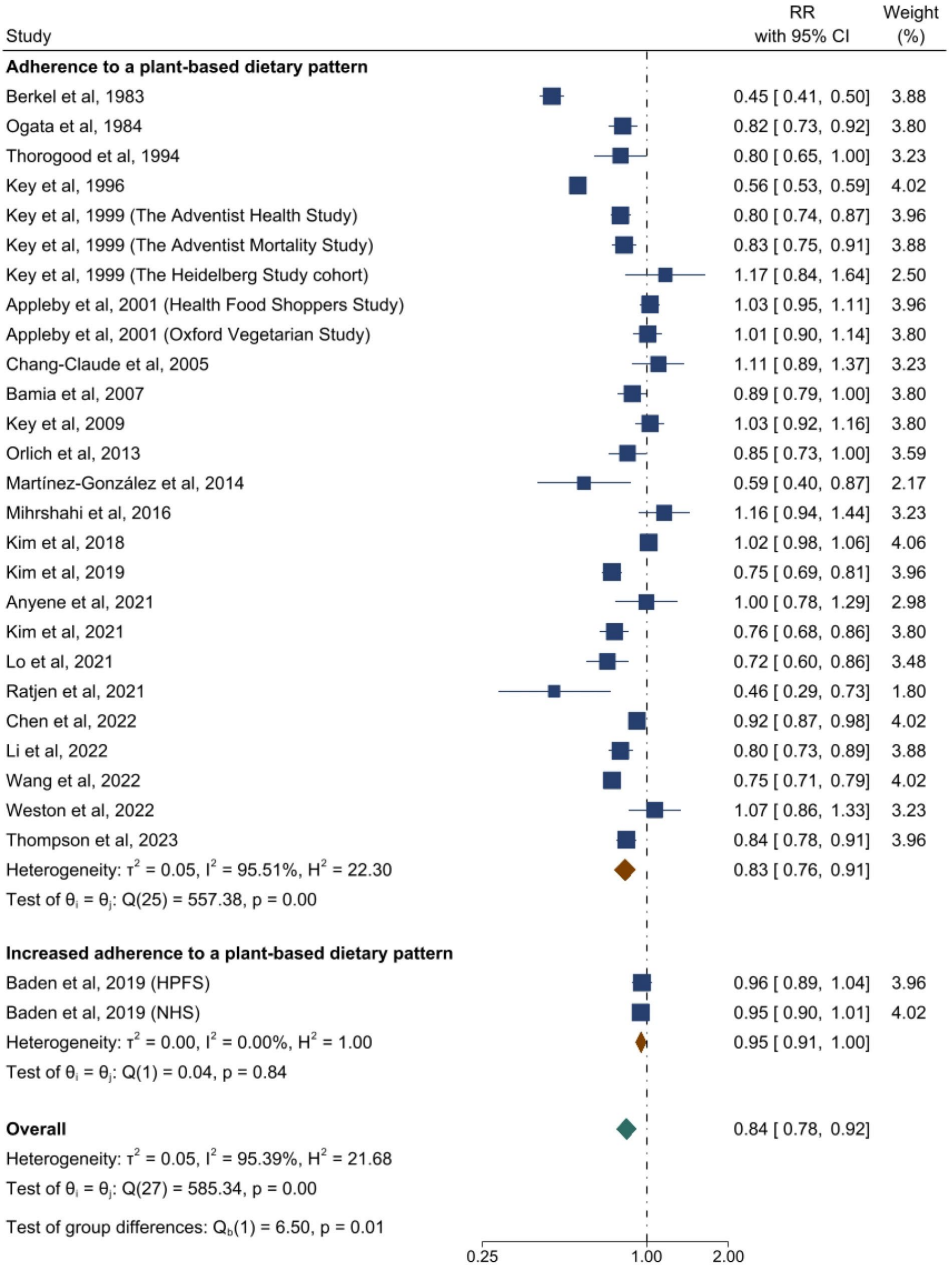
Forest Plot of Studies Examining the Association Between Plant-Based Dietary Patterns and Risks of Mortality using Random-Effects Meta-Analysis **Abbreviations**: HPFS, Health Professionals Follow-up Study; NHS, Nurses’ Health Study

The forest plots for associations between plant-based dietary patterns and T2D, CVD, cancer, and mortality are shown in Figs. 2, 3, 4 and 5. A greater adherence to plant-based dietary patterns was consistently associated with lower risks of T2D, CVD, cancer, and mortality. The random-effects pooled RR was 0.82 (95% CI: 0.77–0.86), 0.90 (95% CI: 0.85–0.94), 0.88 (95% CI: 0.84–0.92), and 0.84 (95% CI: 0.78–0.92), respectively. A moderate heterogeneity was observed among studies for T2D (60.9%), CVD (49.8%), cancer (30.2%), and a high heterogeneity was observed for studies reporting mortality (95.4%).

For individual CVD outcomes, a greater adherence to plant-based dietary pattern was significantly associated with a lower CHD risk (0.86 [95% CI: 0.81–0.91; *I*^2^ = 34.5%]), but not with risks of stroke (0.92 [95% CI: 0.85–1.00; *I*^2^ = 51.6%]) and heart failure (0.89 [95% CI: 0.75–1.06; *I*^2^ = 42.9%]) (Supplemental Figure S1). For various cancer types, plant-based dietary patterns were significantly associated with lower risk of breast cancer (0.91 [95% CI: 0.86–0.95; *I*^2^ = 0%]), digestive system cancer (0.82 [95% CI: 0.72–0.94; *I*^2^ = 0%]), pancreatic cancer (0.68 [95% CI: 0.55–0.84; *I*^2^ = 0%]), and prostate cancer (0.87 [95% CI: 0.77–0.99; I^2^ = 53.3%]), but not with risks of colorectal cancer (0.90 [95% CI: 0.79–1.02; *I*^2^ = 61.8%]), liver cancer (0.51 [95% CI: 0.22–1.21; *I*^2^ = 57.7%]), lung cancer (0.82 [95% CI: 0.54–1.26; *I*^2^ = 36.6%]), or stomach cancer (1.73 [95% CI: 0.90–3.31; *I*^2^ = 0%]) (Supplemental Figure S2). For specific causes of mortality, plant-based dietary pattern was associated with lower risks of CVD mortality (0.79 [95% CI: 0.72–0.87; *I*^2^ = 77.0%]) and cancer mortality (0.82 [95% CI: 0.71–0.93; *I*^2^ = 88.4%]) (Supplemental Figure S3).

The associations between plant-based dietary pattern and T2D, CVD, cancer, and mortality remained similar when using an inverse-variance fixed-effects model (Supplemental Figures S4-S7) and when excluding any single study (Supplemental Figure S8). When using the “healthful plant-based dietary index” instead of the “overall plant-based dietary index”, results were strengthened for T2D (0.79 [95% CI: 0.72–0.87; *I*^2^ = 84.1%]) (Supplemental Figure S9 panel A), CVD (0.85 [95% CI: 0.80–0.92; *I*^2^ = 62.1%]) (Supplemental Figure S9 panel B) and cancer (0.87 [95% CI: 0.82–0.92; *I*^2^ = 53.1%]) (Supplemental Figure S9 panel C); while results were similar for mortality (0.86 [95% CI: 0.80–0.92; *I*^2^ = 91.9%]) (Supplemental Figure S9 panels D). Of note, unlike results summarized for “overall plant-based dietary index”, there were significant associations between the healthful plant-based dietary pattern with stroke (0.89 [95% CI: 0.83–0.95; *I*^2^ = 0%]) (Supplemental Figure S9 panel B) and colorectal cancer (0.85 [95% CI: 0.79–0.92; *I*^2^ = 0%]) (Supplemental Figure S9 panel C). On the contrary, adherence to unhealthful plant-based dietary patterns was positively associated with the risks of major chronic diseases (Supplemental Figure S10). Specifically, higher adherence to an unhealthful plant-based dietary patterns was associated with an increased risk of these outcomes by 8% for T2D (95% CI: 2–13%; *I*^2^ = 15.4%); 14% for CVD (95% CI: 4–26%; *I*^2^ = 71.1%); 7% for cancer (95% CI: 2–12%; *I*^2^ = 46.3%); and 16% for mortality (95% CI: 9–25%; *I*^2^ = 84.8%).

For outcomes of T2D and mortality, data were available to evaluate the impact of change of dietary patterns. Increased adherence to a plant-based dietary pattern was associated with lower risks of T2D (0.83 [95% CI: 0.71–0.96; *I*^2^ = 71.5%]) and a marginally lower risk of mortality (0.95 [95% CI: 0.91–1.00; *I*^2^ = 0%]) (Figs. 2 and 5). Among these studies, only two studies examined changes of hPDI (healthy plant-based diet index) and uPDI (unhealthy plant-based diet index) for T2D and mortality (Supplemental Figure S9–S10) [16, 32].

### Subgroup results and meta-regression

The stratified analyses by study characteristics are shown in Table 1. Overall, we did not observe significant heterogeneity by study region, dietary classification, age, BMI, sex, and follow-up duration for T2D and mortality. The association between plant-based dietary patterns and CVD was stronger in studies conducted in Asia and Australia compared to those conducted in North America and Europe (*P*-heterogeneity = 0.02). The association for T2D was stronger in studies that examined vegan or vegetarian diets compared to studies reporting on a priori defined plant-based diets (*P*-heterogeneity = 0.048). No other sources of heterogeneity were observed for CVD and cancer associations.

**Table 1:** Subgroup Analysis of Plant-Based Dietary Patterns and Risks of Incident Type 2 Diabetes, Cardiovascular Disease, Cancer, and Mortality

Characteristics	Study estimates, No.	Relative Risk (95% CI)			
		Inverse-Variance Fixed-Effects Meta-analysis	Random-Effects Meta-analysis	*I*^*2*^, %	P value for Heterogeneity Between Subgroups
**Type 2 diabetes**					
Main estimate	22	0.84 (0.81–0.86)	0.81 (0.77–0.86)	59.7	
Age, y					0.56
<55	11	0.81 (0.78–0.85)	0.79 (0.73–0.87)	68.7	
≥55	11	0.85 (0.82–0.89)	0.84 (0.79–0.89)	42.0	
Sex					0.48
Studies among males only	2	0.81 (0.72–0.90)	0.79 (0.63–0.99)	75.4	
Studies among females only	6	0.86 (0.82–0.89)	0.85 (0.79–0.91)	64.8	
Studies among males and females	14	0.82 (0.78–0.86)	0.81 (0.77–0.86)	58.3	
BMI, kg/m^2^					0.90
<25	11	0.83 (0.79–0.86)	0.81 (0.75–0.88)	67.8	
≥25	11	0.84 (0.81–0.88)	0.82 (0.76–0.87)	51.8	
Region					0.87
North America	12	0.85 (0.82–0.89)	0.82 (0.76–0.88)	60.1	
Europe	4	0.78 (0.72–0.84)	0.79 (0.71–0.87)	43.5	
Asia/Australia	6	0.83 (0.79–0.88)	0.82 (0.73–0.91)	66.4	
Dietary pattern classification					0.048
Vegan or vegetarian diets	5	0.75 (0.67–0.84)	0.63 (0.47–0.84)	77.4	
A priori-defined PDI	17	0.84 (0.82–0.87)	0.83 (0.80–0.87)	48.3	
Follow-up duration, y					0.22
<15	11	0.81 (0.77–0.86)	0.76 (0.68–0.84)	66.3	
≥15	11	0.84 (0.81–0.86)	0.84 (0.79–0.88)	51.5	
**Cardiovascular disease**					
Main estimate	23	0.90 (0.87–0.92)	0.89 (0.85–0.94)	51.0	
Age, y					0.88
<55	16	0.89 (0.85–0.93)	0.87 (0.80–0.96)	63.8	
≥55	7	0.90 (0.87–0.93)	0.89 (0.85–0.93)	0	
Sex					0.68
Studies among males only	3	0.94 (0.85–1.04)	0.94 (0.85–1.04)	0	
Studies among females only	6	0.90 (0.85–0.94)	0.90 (0.85–0.94)	0	
Studies among males and females	14	0.89 (0.86–0.93)	0.86 (0.79–0.97)	68.1	
BMI, kg/m^2^					0.76
<25	7	0.91 (0.85–0.98)	0.87 (0.72–1.05)	77.9	
≥25	12	0.90 (0.87–0.93)	0.90 (0.87–0.93)	0	
Region					0.02
North America	14	0.89 (0.86–0.93)	0.89 (0.85–0.94)	16.2	
Europe	7	0.91 (0.87–0.95)	0.91 (0.83-1.00)	71.5	
Asia/Australia	2	0.52 (0.35–0.76)	0.52 (0.35–0.76)	0	
Dietary classification					0.99
Vegan or vegetarian diets	5	0.90 (0.86–0.95)	0.85 (0.70–1.03)	85.0	
A priori-defined PDI	18	0.90 (0.87–0.93)	0.90 (0.86–0.93)	6.1	
Follow-up duration, y					0.45
<15	8	0.91 (0.87–0.95)	0.86 (0.77–0.96)	46.0	
≥15	15	0.89 (0.86–0.93)	0.90 (0.85–0.96)	55.9	
**Cancer**					
Main estimate	32	0.90 (0.87–0.92)	0.88 (0.85–0.92)	29.0	
Age, y					0.86
<55	14	0.88 (0.85–0.92)	0.88 (0.85–0.92)	0	
≥55	16	0.91 (0.87–0.94)	0.88 (0.82–0.94)	50.2	
Sex					0.40
Studies among males only	5	0.89 (0.84–0.95)	0.83 (0.70–0.98)	75.3	
Studies among females only	12	0.90 (0.86–0.94)	0.90 (0.86–0.94)	0	
Studies among males and females	15	0.89 (0.86–0.93)	0.87 (0.82–0.92)	18.5	
BMI, kg/m^2^					0.51
<25	12	0.90 (0.87–0.94)	0.90 (0.87–0.94)	0	
≥25	19	0.89 (0.86–0.93)	0.87 (0.81–0.92)	41.0	
Region					0.39
North America	20	0.89 (0.85–0.92)	0.86 (0.81–0.91)	47.7	
Europe	12	0.91 (0.87–0.95)	0.91 (0.86–0.95)	0	
Asia/Australia					
Dietary classification					0.14
Vegan or vegetarian diets	16	0.85 (0.80–0.91)	0.85 (0.80–0.91)	0	
A priori-defined PDI	16	0.91 (0.88–0.93)	0.90 (0.85–0.94)	43.0	
Follow-up duration, y					0.98
<15	15	0.90 (0.86–0.94)	0.87 (0.81–0.94)	38.2	
≥15	17	0.89 (0.86–0.93)	0.88 (0.84–0.93)	23.4	
**Mortality**					
Main estimate	32	0.86 (0.84–0.87)	0.85 (0.79–0.91)	94.6	
Age, y					0.77
<55	17	0.87 (0.85–0.88)	0.88 (0.80–0.96)	95.9	
≥55	12	0.88 (0.85–0.90)	0.86 (0.80–0.94)	83.9	
Sex					0.66
Studies among males only	3	0.94 (0.91–0.97)	0.92 (0.86–0.99)	66.4	
Studies among females only	3	0.85 (0.82–0.88)	0.90 (0.77–1.04)	91.6	
Studies among males and females	26	0.83 (0.82–0.85)	0.83 (0.76–0.91)	95.5	
BMI, kg/m^2^					0.24
<25	12	0.89 (0.87–0.91)	0.90 (0.84–0.95)	86.6	
≥25	11	0.83 (0.80–0.86)	0.84 (0.77–0.92)	77.0	
Region					0.56
North America	13	0.89 (0.87–0.90)	0.87 (0.81–0.94)	92.6	
Europe	14	0.75 (0.73–0.78)	0.80 (0.67–0.96)	96.4	
Asia/Australia	5	0.88 (0.84–0.92)	0.86 (0.76–0.97)	80.9	
Dietary classification					0.90
Vegan or vegetarian diets	14	0.76 (0.74–0.78)	0.86 (0.73–1.01)	96.4	
A priori-defined PDI	18	0.89 (0.87–0.90)	0.85 (0.80–0.91)	90.6	
Follow-up duration, y					0.16
<15	20	0.80 (0.78–0.82)	0.81 (0.74–0.89)	91.2	
≥15	12	0.88 (0.87–0.90)	0.90 (0.80-1.00)	97.1	

### Assessment of publication bias and risk of bias in individual studies

Visual inspection of the funnel plot suggests some degree of publication bias. Four studies lay outside of the pseudo 95% CI of the funnel plot for T2D and CVD, five for cancer, while 16 studies lay outside for mortality (Supplemental Figure S11). Furthermore, Egger regression tests and Begg-Mazumdar regression tests suggested potential publication bias for T2D (*P* = 0.0001 and *P* = 0.01, respectively) and CVD (*P* = 0.04 and *P* = 0.21); but tests did not detect significant publication bias for cancer (*P* = 0.12 and *P* = 0.96) and mortality (*P* = 0.83 and *P* = 0.52). After performing the trim-and-fill analysis to evaluate the robustness of associations after accounting for potential publication bias, our results remained largely unchanged. The random-effects pooled RR was 0.84 (95% CI: 0.80–0.90) for T2D, 0.91 (95% CI: 0.86–0.96) for CVD, 0.91 (95% CI: 0.87–0.95) for cancer and 0.83 (95% CI: 0.76–0.90) for mortality (Supplemental Figure S12).

Common problems affecting the study quality included the lack of sample size justification, the absence of repetitive measurement of exposure measures, and not having information on the loss of follow-up. The detailed study quality assessment is shown in Supplemental Table S4. One publication for T2D risk (5.9%) and 7 publications for mortality (25.9%) had low study quality (< 10). After excluding these studies, the associations remained significant. The random-effects pooled RRs were 0.82 (95% CI: 0.77–0.86) for T2D and 0.87 (95% CI: 0.82–0.92) for mortality. All publications for CVD and cancer were deemed to have high study quality (≥ 10).

## Discussion

In this meta-analysis of 55 prospective cohort studies with 2,230,443 participants, we found that greater adherence to a plant-based dietary pattern was inversely associated with risks of T2D, CVD, cancer and all-cause mortality. Associations for T2D, CVD, and cancer were strengthened when the plant-based diets further emphasized healthful plant-based foods, such as vegetables, fruits, whole grains, and legumes. These findings were largely consistent across subgroups of study characteristics and robust in sensitivity analyses. For specific disease outcomes, the inverse association for CVD was mainly driven by CHD, and for cancer by breast cancer, pancreatic cancer, and prostate cancer. In contrast, the inverse association for mortality was observed for both CVD mortality and cancer mortality. Among studies with measurements of changes in dietary patterns, increased adherence to plant-based dietary patterns was inversely associated with lower risks of T2D and mortality.

The current analysis was consistent with previous systematic reviews and meta-analyses on vegetarian diets and together suggest that plant-based dietary patterns, including the vegan or vegetarian diets, were inversely associated with CVD risk, which was mainly driven by CHD but not stroke [6, 8]. A prospective cohort study in the U.S. found that plant-based diets wre inversely associated with ischemic stroke risk but not with hemorrhagic stroke [41], suggesting that the null association between plant-based diets and total stroke might be due to the heterogenous pathophysiology of stroke subtypes and the relatively smaller number of cases compared to CHD. In terms of heart failure, since only two studies examined the association with plant-based diets and had mixed results [23, 42], the non-significant association is likely to be underpowered and inconclusive and needs further assessment in future studies. For T2D and mortality, our results were consistent with previous meta-analyses that higher adherence to plant-based dietary patterns is associated with lower risks of T2D, all-cause mortality, cancer mortality, and CVD mortality [5, 43], although earlier meta-analyses with fewer studies failed to find significant associations with all-cause mortality or cancer mortality [7, 9, 44]. We further extended the evidence to changes in dietary patterns and showed that increased adherence to plant-based dietary patterns is also associated with lower risks of T2D and mortality. Furthermore, previous meta-analyses on vegetarian diets have also found an inverse association between plant-based dietary patterns with total cancer [7, 9–11]. While we found that the inverse association with cancer is mainly driven by breast, pancreatic, and prostate cancer, but not by colorectal, liver, lung, or stomach cancer. Since the study points for the latter four cancers (3–8 studies) were fewer than those for breast cancer (11 studies), the observed null association could also be due to the limited statistical power, and future studies are needed to investigate associations between plant-based diets and different types of cancer.

In the current study, the inverse association of plant-based diet with T2D, CVD, and cancer were strengthened when we summarized data of studies that examined healthy plant-based patterns. Similarly, previous prospective cohort studies also found stronger associations with healthy plant-based dietary patterns compared to overall plant-based patterns on T2D, CVD and cancer outcomes [5, 25, 45]. In light of existing evidence suggesting that unhealthy plant-based dietary patterns that are rich in refined carbohydrates are detrimental for T2D and CHD risk, the current evidence provides strong evidence to support increased consumption of high-quality plant-based diets, including whole grains, legumes, nuts, fruits, and vegetables.

Several mechanisms for the favorable effect of plant-based dietary patterns on CVD, cancer, and T2D have been proposed previously. As obesity is the common risk factor for CVD, T2D, and certain types of cancer (4), a healthful plant-based diet that emphasizes intakes of fruits, vegetables, whole grains, nuts and legumes, and healthy vegetable oils is likely to be low in energy density due to its low saturated fat and high fiber content, which could help with weight loss and long-term weight maintenance (46). Evidence from clinical trials and observational studies showed that higher intakes of plant-based diets could lead to short-term weight loss or prevention of long-term weight gain (47–50), which may mitigate risks of CVD, cancer, and T2D. In addition, CVD and T2D share some common pathophysiology (51) such as insulin resistance (52), inflammation (53, 54), oxidative stress (55), high blood pressure (56), and dyslipidemia (57, 58). A meta-analysis of randomized clinical trials suggested that higher fiber content has potential cholesterol-lowering effect (59). Similarly, high unsaturated fat and low saturated fat content may also improve the blood lipid profile (60), and replacing saturated fats with unsaturated fat has shown to enhance insulin sensitivity (61) and activate anti-inflammatory pathways (62). Moreover, plant foods are rich in polyphenols (e.g., flavonoids, lignans, phenolic acids), which may lower the risk of CVD (63). Other nutrients such as vitamin C, vitamin E, beta-carotene, potassium and magnesium have shown effects in reducing blood pressure (64), and improving glucose metabolism and insulin sensitivity (65). In addition, recent studies have shed light on the relationship between plant-based dietary patterns and specific circulating metabolites as well as a healthy composition of gut microbiome, which were associated with decreased risks of T2D and CVD. Specifically, Wang and colleagues constructed multi-metabolite profiles for plant-based dietary patterns using data of 10,684 participants from three prospective cohorts (NHS, NHSII, HPFS), where key metabolites in constructing the scores included trigonelline, hippurate, isoleucine and a subset of triacylglycerols (66). They identified that higher scores of multi-metabolite profiles for PDI and hPDI were significantly associated with lower risk of incident T2D (PDI: HR per 1 SD higher = 0.81 [0.75–0.88]), hPDI: (0.77 [0.71–0.84]) (66). The associations between plant-based dietary patterns and metabolic risks might also be modified by gut microbiome. In the Men’s Lifestyle Validation Study (MLVS), individuals with higher hPDI had a distinct gut microbiome signature, which was primarily marked by a higher presence of *Bacteroides cellulosilyticus* and *Eubacterium eligens*, as well as enriched pathways for branched-chain amino acid (BCAA) biosynthesis and depleted pathways for processing animal-derived components [67]. Both the overall microbial profile and individual species play a role in enhancing the favorable associations between hPDI and metabolic health.

In addition, plant-based diets contain no or less consumption of red and processed meat, the intake of which has been associated with higher risks of CVD, T2D, and various cancer types such as breast, colorectal, and lung cancers [5, 68, 69]. Red meat may cause disrupted insulin signaling and increased inflammation and oxidative stress through several components including heme iron [70], nitrates [71], advanced glycation end products and trimethylamine N-oxide [72], and inflammatory mediators [73]. In terms of cancer, plant-based food have well-known anticarcinogenic properties, and thus likely to exert anti-inflammatory and anti-oxidative effects that are against the development of cancer [74].

Our findings have important clinical and public health implications. First, our results suggest that plant-based dietary patterns, especially those based on healthy plant food sources, may be beneficial for the primary prevention of T2D, CVD, cancer, and mortality. A comparative analysis of the health and climate change benefits of global dietary changes projected that moving to diets with fewer animal-sourced foods would result in around 5–8 million avoidable deaths and ~ 200 million life years saved in 2050 [75]. In addition, it would also have environmental benefits by easing pressure on land use and reducing greenhouse gas emissions by 29–70% [75]. Second, our findings corroborated various dietary guidelines, such as those from American Heart Association [76], American Diabetes Association [77], and American Cancer Society [78], which recommend eating an overall healthy dietary pattern that emphasizes a wide variety of fruits and vegetables, whole grains and healthy sources of proteins to prevent CVD, T2D, and cancer. In the current study, we stratified the analysis by dietary classification, and found that people who consumed strict vegetarian patterns and those who consumed plant-based dietary patterns including some animal foods had similar protective effect for CVD, T2D, and mortality. Plant-based diets that do not completely exclude animal products can be easier to adopt than strict vegan or vegetarian diets which are practiced by a small proportion of the total population [79].

The main strength of the current study was the comprehensive assessment of all available prospective studies on various outcomes including CVD, cancer, T2D, and mortality. We evaluated adherence to and changes in plant-based dietary patterns and examined the effect of vegetarian diets and plant-based dietary indices. We estimated effects from both overall and healthy plant-based dietary patterns and evaluated overall disease outcomes and specific disease subtypes. We assessed the robustness of the results by using different statistical methods and observed consistent results across various subgroups.

Nevertheless, some limitations merit consideration. First, since dietary intake was self-reported using food frequency questionnaire or 24-hour recall in included studies, and majority of the studies did not have repeated measurements of the dietary intake, measurement error and misclassification may exist. However, it is likely to be nondifferential among people who developed CVD, cancer, T2D, or mortality and those who did not, which may result in an underestimation of the true effect size of associations between plant-based patterns and chronic diseases. Second, all included studies were prospective cohort studies; therefore, unmeasured or residual confounding factors cannot be ruled out. In addition, included studies adjusted for different covariates in the multivariable models, had different socio-demographic and clinical characteristics, and thus may contribute to the observed moderate-to-high heterogeneity across studies. However, results from random-effects models were similar to those from fix-effects models, and subgroup results were largely similar. Third, study points on some diseases are limited (e.g., heart failure, lung cancer, liver cancer, stomach cancer), and therefore it is unknown whether the observed non-significant association is true or due to the lack of statistical power. Future studies with larger sample sizes are warranted to examine associations of these diseases and plant-based dietary patterns.

## Conclusions

In conclusion, higher adherence to plant-based dietary patterns, especially from healthy sources, may be universally beneficial for the primary prevention of T2D, CVD, cancer, and mortality. The current analysis provides further evidence in support of the current recommendations that emphasize consuming high-quality plant-based foods for achieving an optimal health. Future studies are needed to elucidate associations for certain individual health outcomes, as well as mechanistic pathways linking plant-based diets with multiple disease outcomes.

### Electronic supplementary material

Below is the link to the electronic supplementary material.

Additional file 1

Additional file 2

## Data Availability

Not applicable.

## List of abbreviations

CHD: Coronary heart disease
CI: Confidence interval
CVD: Cardiovascular disease
hPDI: Healthful plant-based diet index
MOOSE: Meta-analysis Of Observational Studies in Epidemiology
PDI: Plant-based diet index
PRISMA: Preferred Reporting Items for Systematic reviews and Meta-Analyses
RR: Relative risk
T2D: Type 2 diabetes
uPDI: Unhealthful plant-based diet index
