# Correction: Associations Between Plant-Based Dietary Patterns and Risks of Type 2 Diabetes, Cardiovascular Disease, Cancer, and Mortality – A Systematic Review and Meta-analysis

**DOI:** 10.1186/s12937-023-00891-4

**Published:** 2024-01-04

**Authors:** Yeli Wang, Binkai Liu, Han Han, Yang Hu, Lu Zhu, Eric B. Rimm, Frank B. Hu, Qi Sun

**Affiliations:** 1 Department of Nutrition, Harvard T.H. Chan School of Public Health, Boston, Massachusetts USA; 2 CAS Key Laboratory of Nutrition, Metabolism and Food Safety, Shanghai Institute of Nutrition and Health, University of Chinese Academy of Sciences, Chinese Academy of Sciences, Shanghai, China; 3 Department of Epidemiology, Harvard T.H. Chan School of Public Health, Boston, Massachusetts USA; 4 Channing Division of Network Medicine, Department of Medicine, Brigham and Women’s Hospital and Harvard Medical School, Boston, Massachusetts USA

**Correction: Nutr J 22, 46 (2023)**

**https://doi.org/10.1186/s12937-023-00877-2**

Following publication of the original article [1], the authors reported an error in data extraction. This error affected some cancer-related results, however the conclusions and key findings remain unchanged after the error was corrected.

Specifically, relative risks extracted from Fraser (1999) [2] were misinterpreted. The title of Table 7 by Fraser is “Incidence and relative risk of common cancers in Seventh-day Adventist vegetarians compared with nonvegetarians”, suggests that the associations are for vegetarians, using the non-vegetarians as the comparison group, but the text in the Results section suggests the opposite. The authors regret not reading the text carefully.

All changes before and after the correction are presented in Table 1. The section of the content of the updated manuscript are also included.

**Table 1:** List of changes in text. Corrected texts are underlined, and original texts are placed in square brackets in italic. The text deleted are marked with strikethrough

**Summary of change**	**Section**	**Text change**
Effect estimates for overall cancer and heterogeneity	Abstract: Results (Page 1)	An inverse association was observed between higher adherence to a plant-based dietary pattern and risks of… cancer (0.88 [0.84–0.92]) [*(0.91 [0.87–0.96])]* … with moderate to high heterogeneity across studies (*I*^*2*^ ranged: 30.2%*[47.8%] *–95.4%,).
	Results: *Plant-Based Diet and Risk of T2D, CVD, cancer, and mortality *(Page 5-6)	A greater adherence to plant-based dietary patterns was consistently associated with lower risks of T2D, CVD, cancer, and mortality. The random-effects pooled RR was 0.82 (95% CI: 0.77–0.86), 0.90 (95% CI: 0.85–0.94), 0.88 (95% CI: 0.84–0.92),*[0.91 (95% CI: 0.87–0.96)]*, and 0.84 (95% CI: 0.78–0.92), respectively. A moderate heterogeneity was observed among studies for T2D (60.9%), CVD (49.8%), cancer (30.2%)*[47.8%],* and a high heterogeneity was observed for studies reporting mortality (95.4%).
Effect estimates for cancer subtypes and heterogeneity	Results: *Plant-Based Diet and Risk of T2D, CVD, cancer, and mortality *(Page 6)	For various cancer types, plant-based dietary patterns were significantly associated with lower risk of breast cancer (0.91 [95% CI: 0.86–0.95; *I*^2^=0%])*[(0.92 [95% CI: 0.87–0.97; I*^*2*^*=11.1%])],* digestive system cancer (0.82 [95% CI: 0.72–0.94; *I*^2^=0%]), pancreatic cancer (0.68 [95% CI: 0.55–0.84; *I*^2^=0%]) and prostate cancer (0.87 [95% CI: 0.77–0.99; *I*^2^=53.3%]), but not with risks of colorectal cancer (0.90 [95% CI: 0.79–1.02; *I*^2^=61.8%])*[(0.98 [95% CI: 0.85–1.12; I*^*2*^*=68.4%])]*, liver cancer (0.51 [95% CI: 0.22–1.21; *I*^2^=57.7%])*[(0.74 [95% CI: 0.52–1.05; I*^*2*^*=48.1%])]*, lung cancer (0.82 [95% CI: 0.54–1.26; *I*^2^=36.6%]) *[(0.88 [95% CI: 0.56–1.38; I*^*2*^*=44.2%])]*, prostate cancer (0.94 [95% CI: 0.81–1.08; I^2^ =60.7%]) or stomach cancer (1.73 [95% CI: 0.90–3.31; *I*^2^=0%]) (Supplemental Figure S2).
Test estimates for Publication bias	Results*: Assessment of Publication Bias and Risk of Bias in Individual Studies *(Page 9)	Furthermore, Egger regression tests and Begg-Mazumdar regression tests … did not detect significant publication bias for cancer (P=0.12 and P=0.96)*[(P=0.07 and P=0.22)]* and mortality (P=0.83 and P=0.52).
Effect estimates after performing the trim-and-fill analysis	Results*: Assessment of Publication Bias and Risk of Bias in Individual Studies *(Page 9)	After performing the trim-and-fill analysis to evaluate the robustness of associations after accounting for potential publication bias, our results remained largely unchanged. The random-effects pooled RR was … 0.91 (95% CI: 0.87–0.95)*[0.89 (95% CI: 0.84–0.94)]* for cancer …
Significancy change of prostate cancer results	Discussion (Page 9)	For specific disease outcomes, the inverse association for CVD was mainly driven by CHD, and for cancer by breast cancer, and pancreatic cancer, and prostate cancer.
	Discussion (Page 12)	While we found that the inverse association with cancer is mainly driven by breast cancer, and pancreatic cancer, and prostate cancer, but not by colorectal, liver, lung, prostate, or stomach cancer.
	Discussion (Page 13)	Third, study points on some diseases are limited (e.g., heart failure, lung cancer, liver cancer, prostate cancer, stomach cancer), and therefore it is unknown whether the observed non-significant association is true or due to the lack of statistical power.

The original Table 1, Figure 4, Supplementary Figure S2, S6, S8, S11–S12 are updated, and the original and corrected table and figures are presented below.

**Original Table 1**

**Table:** 

		Relative Risk (95% CI)			
Characteristics	Study estimates, No.	Inverse-Variance Fixed-Effects Meta-analysis	Random-Effects Meta-analysis	*I*^*2*^, %	P value for Heterogeneity Between Subgroups
**Cancer**					
Main estimate	32	0.91 (0.88-0.93)	0.91 (0.86-0.95)	47.5	-
Age, y					0.08
<55	14	0.92 (0.88-0.96)	0.97 (0.89-1.05)	53.1	
≥55	16	0.91 (0.87-0.94)	0.88 (0.82-0.94)	50.2	
Sex					0.29
Studies among males only	5	0.89 (0.84-0.95)	0.83 (0.70-0.98)	75.3	
Studies among females only	12	0.90 (0.86-0.94)	0.90 (0.86-0.94)	0	
Studies among males and females	15	0.92 (0.88-0.93)	0.95 (0.87-1.04)	55.4	
BMI, kg/m^2^					0.51
<25	12	0.90 (0.87-0.94)	0.90 (0.87-0.94)	0	
≥25	19	0.92 (0.89-0.95)	0.94 (0.87-1.01)	60.0	
Region					0.76
North America	20	0.91 (0.88-0.94)	0.92 (0.85-0.99)	63.5	
Europe	12	0.91 (0.87-0.95)	0.91 (0.86-0.95)	0	
Asia/Australia	-	-	-	-	-
Dietary classification					0.35
Vegan or vegetarian diets	16	0.93 (0.87-0.99)	0.96 (0.85-1.07)	53.2	
A priori-defined PDI	16	0.91 (0.88-0.93)	0.90 (0.85-0.94)	43.0	
Follow-up duration, y					0.08
<15	15	0.93 (0.89-0.97)	0.97 (0.88-1.06)	60.7	
≥15	17	0.89 (0.86-0.93)	0.88 (0.84-0.93)	23.4	
Main estimate	32	0.90 (0.87-0.92)	0.88 (0.85-0.92)	29.0	-
Age, y					0.86
<55	14	0.88 (0.85-0.92)	0.88 (0.85-0.92)	0	
≥55	16	0.91 (0.87-0.94)	0.88 (0.82-0.94)	50.2	
Sex					0.40
Studies among males only	5	0.89 (0.84-0.95)	0.83 (0.70-0.98)	75.3	
Studies among females only	12	0.90 (0.86-0.94)	0.90 (0.86-0.94)	0	
Studies among males and females	15	0.89 (0.86-0.93)	0.87 (0.82-0.92)	18.5	
BMI, kg/m^2^					0.51
<25	12	0.90 (0.87-0.94)	0.90 (0.87-0.94)	0	
≥25	19	0.89 (0.86-0.93)	0.87 (0.81-0.92)	41.0	
Region					0.39
North America	20	0.89 (0.85-0.92)	0.86 (0.81-0.91)	47.7	
Europe	12	0.91 (0.87-0.95)	0.91 (0.86-0.95)	0	
Asia/Australia	-	-	-	-	-
Dietary classification					0.14
Vegan or vegetarian diets	16	0.85 (0.80-0.91)	0.85 (0.80-0.91)	0	
A priori-defined PDI	16	0.91 (0.88-0.93)	0.90 (0.85-0.94)	43.0	
Follow-up duration, y					0.98
<15	15	0.90 (0.86-0.94)	0.87 (0.81-0.94)	38.2	
≥15	17	0.89 (0.86-0.93)	0.88 (0.84-0.93)	23.4	


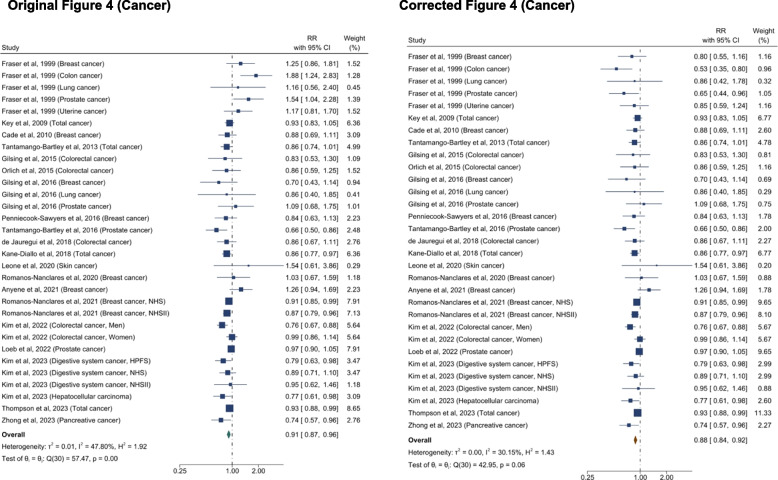



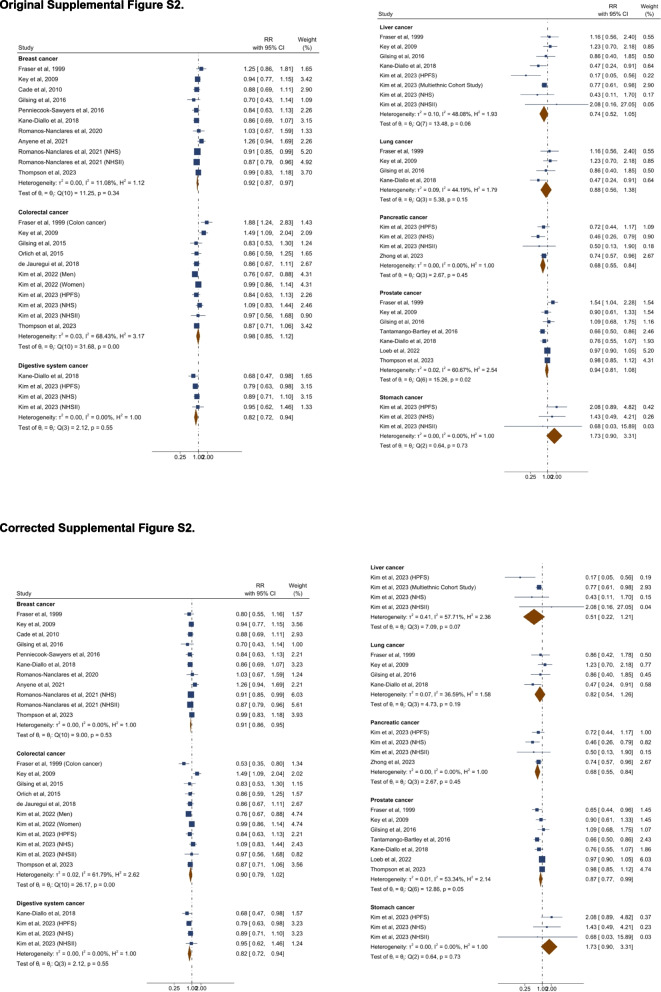



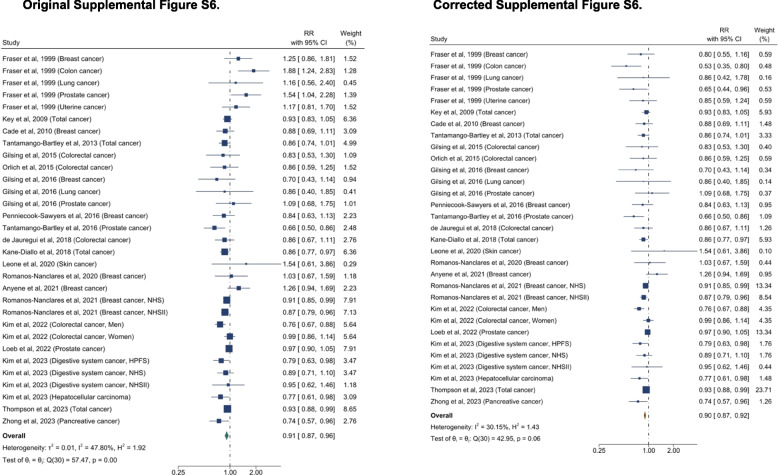



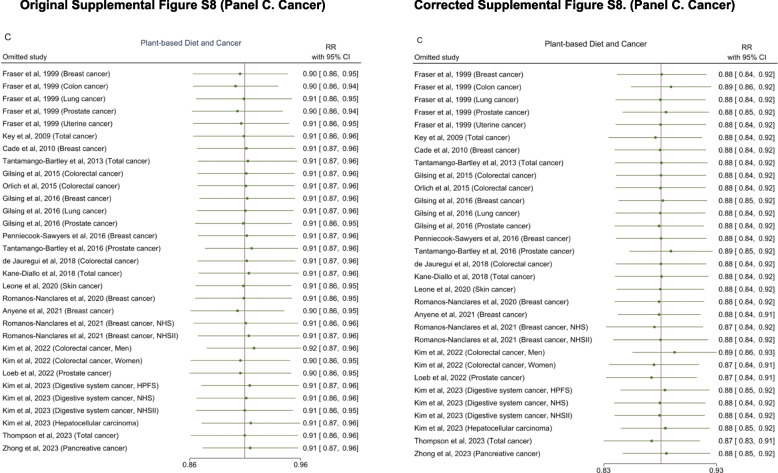



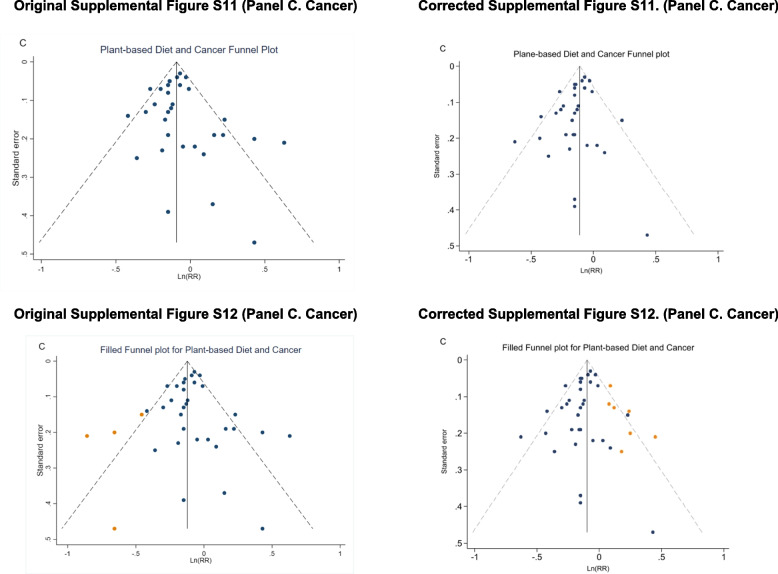


**Acknowledgement**

We thank Ms. Esther Farley for identifying the error which leads to this timely correction.
