# Supplementary material for: Associations between plant-based dietary patterns and risks of type 2 diabetes, cardiovascular disease, cancer, and mortality – a systematic review and meta-analysis
Source: Nutr J. 2023 Oct 4;22:46. doi: 10.1186/s12937-023-00877-2 (PMC10548756; doi:10.1186/s12937-023-00877-2)
Supplement: Supplementary file 2 — Additional file 2 [file 12937_2023_877_MOESM2_ESM.pdf]

Supplemental Table S1. Search Terms and Number of Records.

PubMed

(("Diet, Vegetarian"[Mesh] OR "Diet, Vegan"[Mesh] OR plant-based OR plant-based diet OR vegetarian OR vegan) AND (("Diabetes Mellitus"[Mesh] OR "Diabetes Mellitus, Type 2"[Mesh] OR diabetes OR type 2 diabetes OR type II diabetes OR non-insulin dependent diabetes OR NIDDM) OR ("Cardiovascular Diseases"[Mesh] OR "Stroke"[Mesh] OR "Coronary Disease"[Mesh] OR "Heart Diseases"[Mesh] OR "Coronary Artery Disease"[Mesh] OR "Myocardial Infarction"[Mesh] OR "Angina Pectoris"[Mesh] OR "Heart Failure"[Mesh] OR "Myocardial Ischemia"[Mesh] OR "Ischemic Stroke"[Mesh] OR "Hemorrhagic Stroke"[Mesh] OR "Cerebrovascular Disorders"[Mesh] OR cardiovascular diseases OR stroke OR cardiovascular OR coronary heart disease OR heart disease OR coronary artery disease OR myocardial infarction OR angina pectoris OR heart failure OR CHD OR CVD OR ischemic heart disease OR ischaemic heart disease OR ischaemic stroke OR ischemic stroke OR haemorrhagic stroke OR hemorrhagic stroke OR cerebrovascular disease) OR ("Neoplasms"[Mesh] OR "Carcinoma"[Mesh] OR cancer OR total cancer OR carcinoma OR tumor OR neoplasm) OR ("Mortality"[Mesh] OR "Survival"[Mesh] OR "Death"[Mesh] OR mortality OR all-cause mortality OR total mortality OR survival OR death))) AND (Human)

Records found: 4313

EMBASE

('vegetarian diet'/exp OR 'vegan diet'/exp OR 'plant diet':ti,ab,kw OR 'plant-based diet':ti,ab,kw OR 'vegetarian diet':ti,ab,kw OR 'vegan diet':ti,ab,kw) AND ('diabetes mellitus'/exp OR 'non insulin dependent diabetes mellitus'/exp OR 'diabetes mellitus':ti,ab,kw OR 'non insulin dependent diabetes mellitus':ti,ab,kw OR 'type 2 diabetes':ti,ab,kw OR 'type ii diabetes':ti,ab,kw OR 'cardiovascular disease'/exp OR 'ischemic heart disease'/exp OR 'heart disease'/exp OR 'coronary artery disease'/exp OR 'heart infarction'/exp OR 'angina pectoris'/exp OR 'heart failure'/exp OR 'ischemic stroke'/exp OR 'brain hemorrhage'/exp OR 'cerebrovascular disease'/exp OR 'cardiovascular diseases':ti,ab,kw OR 'stroke':ti,ab,kw OR 'cardiovascular':ti,ab,kw OR 'coronary heart disease':ti,ab,kw OR 'heart disease':ti,ab,kw OR

'coronary artery disease':ti,ab,kw OR 'myocardial infarction':ti,ab,kw OR 'angina pectoris':ti,ab,kw OR 'heart failure':ti,ab,kw OR 'ischemic heart disease':ti,ab,kw OR 'ischaemic heart disease':ti,ab,kw OR 'ischaemic stroke':ti,ab,kw OR 'ischemic stroke':ti,ab,kw OR 'haemorrhagic stroke':ti,ab,kw OR 'hemorrhagic stroke':ti,ab,kw OR 'cerebrovascular disease':ti,ab,kw OR 'malignant neoplasm'/exp OR 'carcinoma'/exp OR 'neoplasm'/exp OR 'cancer':ti,ab,kw OR 'total cancer':ti,ab,kw OR 'carcinoma':ti,ab,kw OR 'tumor':ti,ab,kw OR 'neoplasm':ti,ab,kw OR 'mortality'/exp OR 'all cause mortality'/exp OR 'survival'/exp OR 'death'/exp OR 'mortality':ti,ab,kw OR 'all cause mortality':ti,ab,kw OR 'total mortality':ti,ab,kw OR 'survival':ti,ab,kw OR 'death':ti,ab,kw) AND 'human'/exp AND [embase]/lim

Records found: 2451

Web of Science

TS=(((plant-based OR plant-based diet OR vegetarian OR vegan) AND ((diabetes OR type 2 diabetes OR type II diabetes OR non-insulin dependent diabetes OR NIDDM) OR (cardiovascular diseases OR stroke OR cardiovascular OR coronary heart disease OR heart disease OR coronary artery disease OR myocardial infarction OR angina pectoris OR heart failure OR CHD OR CVD OR ischemic heart disease OR ischaemic heart disease OR ischaemic stroke OR ischamic stroke OR haemorrhagic stroke OR hemorrhagic stroke OR cerebrovascular disease) OR (cancer OR total cancer OR carcinoma OR tumor OR neoplasm) OR (mortality OR all-cause mortality OR total mortality OR survival OR death))))

Records found: 5572

Supplemental Table S2. Inclusion/Exclusion Criteria for Literature Search.

Inclusion criteria

- Prospective cohort studies, prospective case-cohort studies, or nested prospective case-control studies
- Clear definition of dietary exposure (used a priori-defined dietary patterns with emphasis on the plant-based foods and de-emphasis or avoidance on the animal foods) assessed using validated dietary assessment methods
- Multivariate adjusted effect estimates (odds ratio, relative risk, rate ratio, or hazard ratio)
- Human studies

Exclusion criteria

- Retrospective case-control studies, cross-sectional and ecological studies, literature reviews, commentaries, editorials, letters, case reports, and meeting abstracts
- Primary outcome involves conditions that are not type 2 diabetes (such as type 1 diabetes, children with type 2 diabetes, gestational diabetes, prediabetes, or impaired glucose tolerance), cardiovascular disease, cancer, or mortality
- Unclear definitions of dietary exposure or measurements
- Used a posteriori approach (e.g., principal component analysis, factor analysis) to derive dietary patterns
- Crude effect estimates only
- Non-human animal studies
- No full text

Supplemental Table S3. Baseline Characteristics of Published Studies Examining Plant-Based Dietary Patterns and Incident Type 2 Diabetes, Cardiovascular Disease, Cancer, and Mortality.

Source	Study name	Region	Disease outcome	Case/ total number	Mean age (year)	Mean BMI (kg/m^2)	Men (%)	Follow up (year)	Diet assessment	Exposure	Disease ascertainment	Model adjustment
Vang et al (1), 2008	AHS and AMS	United States	T2D	543/ 8,401	64.6	24.5	61.1	Median 17.0	FFQ	Vegetarian, occasional meat intake vs. nonvegetarian	Self-reported	Age, sex, and BMI
Tonstad et al (2), 2013	AHS-2	United States and Canada	T2D	616/ 41,387	58.0	26.7	36.7	Median 2.0	FFQ	Vegan, lacto-ovo-vegetarian, pesco-ovo-vegetarian, semi-vegetarian vs. nonvegetarian	Self-reported with validated questionnaire	Age, BMI, race/ethnicity, sex, educational level, income, television watching, sleep, alcohol intake, physical activity, and smoking
Satija et al (3), 2016	NHS	United States	T2D	7,711/ 69,949	50.0	25.0	0	Maximum 28.0	FFQ	PDI, hPDI, uPDI, comparing extreme deciles	Self-reported with confirmation by a validated supplementary questionnaire, diagnosis criteria per the National Diabetes Data Group	Age, smoking, physical activity, alcohol intake, multivitamin use, family history of diabetes, total energy intake, hypertension, hypercholesterolemia, menopausal status or hormone replacement use, and BMI
Satija et al (3), 2016	NHSII	United States	T2D	5,200/ 90,239	36.0	25.0	0	Maximum 20.0	FFQ	PDI, hPDI, uPDI, comparing extreme deciles	Self-reported with confirmation by a validated supplementary questionnaire, diagnosis criteria per the National Diabetes Data Group	Age, smoking, physical activity, alcohol intake, multivitamin use, family history of diabetes, total energy intake, hypertension, hypercholesterolemia, menopausal status or hormone replacement use, oral contraceptive, and BMI
Satija et al (3), 2016	HPFS	United States	T2D	3,251/ 40,539	53.0	25.2	100	Maximum 24.0	FFQ	PDI, hPDI, uPDI, comparing extreme deciles	Self-reported with confirmation by a validated supplementary questionnaire, diagnosis criteria per the National Diabetes Data Group	Age, smoking, physical activity, alcohol intake, multivitamin use, family history of diabetes, total energy intake, hypertension, hypercholesterolemia, and BMI
Chen et al (4), 2018 (a)	Rotterdam Study I, II, III	The Netherlands	T2D	642/ 6,770	62.0	26.6	41.3	Median 7.3	FFQ	PDI, comparing per 10 units higher score, converted to	Diagnosis information was collected from general practitioners' records, pharmacy	Age, sex, energy intake, Rotterdam Study Sub-cohort, education, smoking, family history of diabetes, physical

Source	Study name	Region	Disease outcome	Case/ total number	Mean age (year)	Mean BMI (kg/m^2)	Men (%)	Follow up (year)	Diet assessment	Exposure	Disease ascertainment	Model adjustment
										comparing extreme quintiles	databases, and follow-up examinations. Confirmation was judged by two physicians, discrepancies were settled by consulting an endocrinologist	activity, food supplement use, and BMI
Chen et al (5), 2018 (b)	Singapore Chinese Health Study	Singapor e	T2D	5,207/ 45,411	55.2	23.0	42.7	Median 11.2	FFQ	PDI, hPDI, comparing extreme quintiles	Self-reported and validated through linkage with nationwide hospital discharge database, supplementary questionnaire, or analysis of blood samples	Age, sex, dialect group, year of interview, energy intake, physical activity, BMI, education, smoking, hypertension, and alcohol use
Chiu et al (6), 2018	The Tzu Chi Health Study	Taiwan	T2D	183/ 2,918	53.2	23.3	81.2	Median 5.0	FFQ	Vegetarian diet, reverted vegetarian, converted vegetarian vs nonvegetarian (Baseline and change in diet)	Self-reported on questionnaires or HbA1c≥6.5%; in cases with uncertain diagnosis, medical record review was performed	Age, sex, education, physical activity, family history of diabetes, follow-up methods (Health examination or questionnaire only), use of lipid medication, and BMI
Papier et al (7), 2019	EPIC-Oxford	United Kingdo m	T2D	1,224/ 65,411	44.5	23.5	76.1	Mean 17.6	FFQ	Vegetarians and vegans, fish eaters, low meat eaters vs. regular meat eaters	Health record linkage to National Health Service Central Registers	Age, education, Townsend deprivation index, ethnicity, smoking, alcohol intake, physical activity, and BMI
Choi et al (8), 2020	The Coronary Artery Risk Development in Young Adults	United States	T2D	206/ 2,534	25.2	24.0	42.6	Mean 9.3	FFQ	20-year change in APDQS comparing extreme quintiles (Change in adherence of plant-based diet)	Fasting glucose concentration ≥126 mg/dL, 2-h post challenge glucose concentration ≥200 mg/dL (Y10, Y20, and Y25), HbA1c ≥6.5% (Y20 and Y25), and/or use of self-reported antidiabetic medications (brought medication bottle)	Age (Y20), sex, race, total energy intake (Y20), parental history of diabetes, physical activity level (Y20), smoking status (Y20), highest grade of education achieved during follow-up, and BMI (Y20).
Chen et al (9), 2021	NHS	United States	T2D	5,993/ 76,530	58.1	25.4	0	Maximu m 26.0	FFQ	Change in PDI, hPDI, uPDI, comparing large	Self-reported with confirmation by a	Age and initial corresponding plant-based diet score, ethnicity, family history of diabetes, initial

Source	Study name	Region	Disease outcome	Case/ total number	Mean age (year)	Mean BMI (kg/m^2)	Men (%)	Follow up (year)	Diet assessment	Exposure	Disease ascertainment	Model adjustment
										increase (>10%) vs. no change (Change in adherence of plant-based diet)	validated supplementary questionnaire, diagnosis criteria per the National Diabetes Data Group	and change in total energy, alcohol intake, margarine intake and physical activity, change in smoking status, initial BMI, history of hypertension, history of hypercholesterolemia, menopausal status, and postmenopausal hormone use
Chen et al (9), 2021	NHSII	United States	T2D	4,190/ 81,569	41.1	24.6	0	Maximum 26.0	FFQ	Change in PDI, hPDI, uPDI, comparing large increase (>10%) vs. no change (Change in adherence of plant-based diet)	Self-reported with confirmation by a validated supplementary questionnaire, diagnosis criteria per the National Diabetes Data Group	Age and initial corresponding plant-based diet score, ethnicity, family history of diabetes, initial and change in total energy, alcohol intake, margarine intake and physical activity, change in smoking status, initial BMI, history of hypertension, history of hypercholesterolemia, menopausal status, postmenopausal hormone use, and oral contraceptive use
Chen et al (9), 2021	HPFS	United States	T2D	2,444/ 34,468	57.5	25.4	100	Maximum 30.0	FFQ	Change in PDI, hPDI, uPDI, comparing large increase (>10%) vs. no change (Change in adherence of plant-based diet)	Self-reported with confirmation by a validated supplementary questionnaire, diagnosis criteria per the National Diabetes Data Group	Age and initial corresponding plant-based diet score, ethnicity, family history of diabetes, initial and change in total energy, alcohol intake, margarine intake and physical activity, change in smoking status, initial BMI, history of hypertension, history of hypercholesterolemia
Flores et al (10), 2021	Boston Puerto Rican Health Study	United States	T2D	134/646	55.5	29.7	28	4.2	FFQ	PDI, hPDI, uPDI, comparing extreme tertiles	Phlebotomist took participants' fasting morning blood draw at their home. Diabetes status was defined as having fasting plasma glucose ≥ 126 mg/dL (7.0 mmol/L), glycated hemoglobin ≥ 6.5% (48 mmol/mol), or use of hypoglycemic agents.	Age, sex, education, marital status, income to poverty ratio, total energy, smoking status, alcohol frequency, physical activity score, psychological acculturation score, depressive symptomatology score, BMI.
Laouali et al (11), 2021	The E3N Prospective Cohort Study	France	T2D	3,292/ 70,991	52.9	22.9	0	~20	FFQ	PDI, hPDI, uPDI, comparing extreme quintiles	Before 2004, T2D cases were identified through self-report and followed up with diabetes-specific questionnaire for	Age, family history of diabetes, educational level, hypercholesterolemia, hypertension, smoking status, physical activity, and energy intake.

Source	Study name	Region	Disease outcome	Case/ total number	Mean age (year)	Mean BMI (kg/m^2)	Men (%)	Follow up (year)	Diet assessment	Exposure	Disease ascertainment	Model adjustment
											validation. After 2004, cases were identified through the drug reimbursement insurance database.	
Yang et al (12), 2021	The Henan Rural Cohort Study	Mainland China	T2D	NA/ 37,985	55.7	24.8	39.3	Maximum 7.0	FFQ	PDI comparing extreme quartiles	Fasting glucose concentration ≥7.0 mmol/L (126 mg/dL) or self-reported T2D diagnosis and/or the use of insulin or blood glucose-lowering drugs in the past 2 weeks	Age, gender, education level, marital status, per capita monthly income, tobacco smoking, alcohol drinking, total energy intake, physical activity, hypertension, family history of diabetes, and BMI
Bhupathiraju et al (13), 2022	Mediators of Atherosclerosis in South Asians Living in America (MASALA) study	United States	T2D	45/735	55.3	26.0	53.0	Mean 5	FFQ	PDI, hPDI, uPDI, per 5 units increment	Defined by the use of a glucose-lowering medication, fasting plasma glucose ⩾7.0 mmol/L, and/or glucose ⩾11.1 mmol/L at 2 hours after the challenge	Age, sex, study site, education, smoking status, alcohol consumption, family history of diabetes, years lived in the United States, physical activity, total energy, diabetes medication use, cholesterol-lowering medication use, hypertension medication use, sum of cultural traditional measures, and BMI
Chen et al (14), 2022 (a)	China Nutrition and Health Survey	China	T2D	720/8,211	46.1	22.9	48.3	Median 10.2	24-hour dietary recall	PDI, hPDI, comparing extreme quintiles	In the 2009 survey, T2D was defined as meeting at least one of the following criteria: (1) fasting blood glucose concentration of ≥7.0 mmol/L (126 mg/dL), (2) HbA1c≥6.5%, or (3) self-reported diagnosis of T2D or on hypoglycemic medication; In 2015, T2D was defined based on self-reported diabetes or taking hypoglycemic medication.	Age, sex, total energy intake, education, physical activity, smoking, alcohol consumption, baseline SBP & DBP, and BMI
Kim and Giovannucci (15), 2022	Korean Genome and Epidemiology Study (KoGES)	Korean	T2D	977/7,393	51.7	24.6	46.4	Maximum 14	FFQ	PDI, hPDI, uPDI, per 10-point increment	Defined by elevated plasma glucose (≥126 mg/dL), self-report of a doctor's diagnosis of	Age, sex, residential area, education, physical activity, smoking, alcohol consumption, baseline BMI, total energy intake,

Source	Study name	Region	Disease outcome	Case/ total number	Mean age (year)	Mean BMI (kg/m^2)	Men (%)	Follow up (year)	Diet assessment	Exposure	Disease ascertainment	Model adjustment
											T2D, or use of oral hypoglycemic drug.	family history of diabetes, and history of hypertension
Glenn et al (16), 2023	WHI	United States	T2D	13,943/145,29 9	63	27.8	0	Median 16	FFQ	Portfolio Diet comparing extreme quintiles.	T2D were defined as a self-report of physician-diagnosed diabetes treated with oral medication or insulin, determined at each annual contact.	Age, region, smoking, study arm, self-identified race and ethnicity, education, marital status, hysterectomy history, physical activity, alcohol intake, energy intake, hypertension status, family history of diabetes, HT use, cholesterol-lowering medication use, BMI.
Zhang et al (17), 2023 (a)	Malmö Diet and Cancer (MDC) study	Sweden	T2D	4,197/24,494	58.1	25.6	38.5	Median 24.3	FFQ	EAT-Lancet diet, comparing extreme levels (≥23 vs. ≤13)	Diabetes cases were retrieved by linking the Swedish personal identification number with eight national and local registers as well as re-examination screenings of the study participants.	Age, sex, dietary assessment version, season, total energy intake, leisure-time physical activity, alcohol consumption, smoking status, educational level, family history of diabetes, lipid-lowering medication, hypertension at baseline, history of cardiovascular disease and cancer, BMI.
Satija et al (18), 2017	NHS	United States	CVD (CHD)	3,233/ 73,710	50.0	25.0	0	Maximu m 28.0	FFQ	PDI, hPDI, uPDI, comparing extreme deciles	Self-reported, confirmed by medical records	Age, smoking status, physical activity, alcohol intake, multivitamin use, aspirin use, family history of CHD, margarine intake, energy intake, baseline hypertension, hypercholesterolemia, and diabetes, updated BMI, and postmenopausal hormone use.
Satija et al (18), 2017	NHSII	United States	CVD (CHD)	667/ 92,329	36.5	25.0	0	Maximu m 22.0	FFQ	PDI, hPDI, uPDI, comparing extreme deciles	Self-reported, confirmed by medical records	Age, smoking status, physical activity, alcohol intake, multivitamin use, aspirin use, family history of CHD, margarine intake, energy intake, baseline hypertension, hypercholesterolemia, and diabetes, updated BMI, postmenopausal hormone use, and oral contraceptive use.
Satija et al (18), 2017	HPFS	United States	CVD (CHD)	4,731/ 43,259	53.5	25.5	100	Maximu m 26.0	FFQ	PDI, hPDI, uPDI, comparing extreme deciles	Self-reported, confirmed by medical records	Age, smoking status, physical activity, alcohol intake, multivitamin use, aspirin use, family history of CHD, margarine intake, energy intake, baseline hypertension,

Source	Study name	Region	Disease outcome	Case/ total number	Mean age (year)	Mean BMI (kg/m^2)	Men (%)	Follow up (year)	Diet assessment	Exposure	Disease ascertainment	Model adjustment
												hypercholesterolemia, and diabetes, and updated BMI.
Kim et al (19), 2019	ARIC	United States	CVD (Total)	4,381/ 12,168	53.8	Obesity (20.7%)	44.1	Median 25	FFQ	PDI, hPDI, uPDI, provegetarian diet index comparing extreme quintiles	Ascertained through annual telephone calls with participants or proxies, active surveillance of local hospital discharge records and state death records, and linkage to the National Death Index	Age, sex, race-center, total energy intake, education, smoking status, physical activity, alcohol consumption, and margarine consumption
Tong et al (20), 2019	EPIC-Oxford	United Kingdo m	CVD (IHD)<br>CVD (Stroke)	2,820/ 48,188<br>1,072/ 48,188	44.7	23.6	22.3	18.1	FFQ	Vegetarians, fish eaters vs. meat eaters	Record linkage to United Kingdom's health service	Age, sex (stratified), method of recruitment (stratified), region (stratified), year of recruitment, education, Townsend deprivation index, smoking, alcohol consumption, physical activity, dietary supplement use, and oral contraceptive and hormone replacement therapy use in women
Chiu et al (21), 2020	The Tzu Chi Health Study	Taiwan	CVD (stroke)	54/ 5,050	52.3	23.6	35.9	Maximu m 7.0	FFQ	Vegetarian vs. nonvegetarian	Record linkage to the National Health Insurance Research Database	Sex, smoking, alcohol drinking, betel nut, leisure time physical activities, education, hypertension, diabetes mellitus, dyslipidemia, ischemic heart disease, and BMI
Chiu et al (21), 2020	The Tzu Chi Vegetarian Study	Taiwan	CVD (stroke)	121/ 8,302	49.5	NA	34.1	Maximu m 9.0	FFQ	Vegetarian vs. nonvegetarian	Record linkage to the National Health Insurance Research Database	Sex, smoking, alcohol drinking, betel nut, leisure time physical activities, education, hypertension, diabetes mellitus, dyslipidemia, ischemic heart disease, and BMI
Shan et al (22), 2020	NHS	United States	CVD (Total)<br>CVD (CHD)<br>CVD (Stroke)	10,562/ 74,930<br>18,092 (3 cohorts)<br>5,687 (3 cohorts)	50.2	24.8	0	Maximu m 32.0	FFQ	hPDI comparing extreme quintiles for total CVD; hPDI per 25 percentile (18 points) increment for CHD and stroke, and converted to comparing extreme quintiles	Self-reported, confirmed by medical records	Age, race/ethnicity, BMI, physical activity, smoking status, alcohol intake, menopausal status, marital status, living alone or with others, family history of myocardial infarction, total energy intake, multivitamin use, and aspirin use
Shan et al (22), 2020	NHSII	United States	CVD (Total)<br>CVD (CHD)	2,029/ 90,864<br>18,092 (3 cohorts)	36.1	24.5	0	Maximu m 26.0	FFQ	hPDI comparing extreme quintiles	Self-reported, confirmed by medical records	Age, race/ethnicity, BMI, physical activity, smoking status, alcohol intake, menopausal status, oral contraceptive use, marital status,

Source	Study name	Region	Disease outcome	Case/ total number	Mean age (year)	Mean BMI (kg/m^2)	Men (%)	Follow up (year)	Diet assessment	Exposure	Disease ascertainment	Model adjustment
			CVD (Stroke)	5,687 (3 cohorts)								living alone or with others, family history of myocardial infarction, total energy intake, multivitamin use, and aspirin use
Shan et al (22), 2020	HPFS	United States	CVD (Total)<br>CVD (CHD)<br>CVD (Stroke)	10,775/ 43,339<br>18,092 (3 cohorts)<br>5,687 (3 cohorts)	53.2	25.4	100	Maximum 26.0	FFQ	hPDI comparing extreme quintiles	Self-reported, confirmed by medical records	Age, BMI, physical activity, smoking status, alcohol intake, marital status, living alone or with others, family history of myocardial infarction, total energy intake, multivitamin use, and aspirin use
Baden et al (23), 2021	NHS	United States	CVD (Stroke)	3,604/ 73,890	50.5	24.9	0	Maximum 32.0	FFQ	PDI, hPDI, uPDI, comparing extreme quintiles	Self-reported with confirmation with additional detail by letter/interview and medical records	Race, physical activity, alcohol consumption, margarine, total energy intake, smoking, aspirin use, multivitamin use, BMI, postmenopausal hormone therapy, hypertension, hypercholesterolemia, diabetes, antihypertensive use, and anticholesterol medication use
Baden et al (23), 2021	NHSII	United States	CVD (Stroke)	740/ 92,352	36.5	24.6	0	Maximum 26.0	FFQ	PDI, hPDI, uPDI, comparing extreme quintiles	Self-reported with confirmation with additional detail by letter/interview and medical records	Race, physical activity, alcohol consumption, margarine, total energy intake, smoking, aspirin use, multivitamin use, BMI, postmenopausal hormone therapy, oral contraceptives, hypertension, hypercholesterolemia, diabetes, antihypertensive use, and anticholesterol medication use
Baden et al (23), 2021	HPFS	United States	CVD (Stroke)	1,897/ 43,266	53.5	25.5	100	Maximum 26.0	FFQ	PDI, hPDI, uPDI, comparing extreme quintiles	Self-reported with confirmation with additional detail by letter/interview and medical records	Race, physical activity, alcohol consumption, margarine, total energy intake, smoking, aspirin use, multivitamin use, BMI, hypertension, hypercholesterolemia, diabetes, antihypertensive use, and anticholesterol medication use
Glenn et al (24), 2021	WHI	United States	CVD (Total)<br>CVD (CHD)<br>CVD (Heart failure)<br>CVD (Stroke)	13,365/ 123,330<br>5,640/ 123,330<br>1,907/ 123,330<br>4,440/ 123,330	62.7	27.8	0	Mean 15.3	FFQ	Plant-based dietary pattern comparing extreme quartiles	Self-reported with confirmation with medical records	Age, region, smoking, and study arm, race/ethnicity, education, marital status, hysterectomy history, BMI, physical activity, alcohol intake, energy intake, cancer status, hypertension status, diabetes mellitus status, sodium intake, family history of CVD, family history of diabetes mellitus,

Source	Study name	Region	Disease outcome	Case/ total number	Mean age (year)	Mean BMI (kg/m^2)	Men (%)	Follow up (year)	Diet assessment	Exposure	Disease ascertainment	Model adjustment
												hormone therapy use, and cholesterol-lowering medication use
Petermann-Rocha et al (25), 2021	UK Biobank	United Kingdom	CVD (Total)<br>CVD (Heart failure)<br>CVD (IHD)<br>CVD (MI)<br>CVD (Stroke)	106,690/ 422,791<br>7,685/ 422,791<br>24,794/ 422,791<br>6,770/ 422,791<br>5,946/ 422,791	56.4	27.3	44.6	Median 8.5	24-hour dietary recall	Vegetarians, fish eaters, fish, and poultry eaters vs. meat-eaters	Record linkage to registries	Age, sex, deprivation, ethnicity, comorbidities, smoking, alcohol intake, total sedentary time, physical activity, and BMI
Chen et al (26), 2022 (b)	Hispanic Community Health Study/Study of Latinos (HCHS/SOL)	United States	CVD	232/10,293	40.9	29.3	41.5	Mean 6	24-hour dietary recall	hPDI, comparing extreme tertiles	Self-reported	Age, sex, field center, Hispanic/Latino background, generational status, education, smoking, alcohol consumption, total energy intake, physical activity, BMI, and use of antidiabetic drugs, antihypertensive drugs, or lipid-lowering drugs
Choi et al (27), 2022	The Coronary Artery Risk Development in Young Adults (CARDIA) cohort	United States	CVD (CHD)<br>CVD (Stroke)	116/4,701<br>80/4,701	24.9	24.4	44.8	Median 32 years	FFQ	A Priori Diet Quality Score (APDQS) comparing extreme quintiles	Annual follow-ups and medical record reviews	Age, sex, race, total energy intake, maximal educational attainment, parental history of CVD, pack-years of smoking, physical activity, use of lipid-lowering medications, and BMI.
Ibsen et al (28), 2022	Danish Diet, Cancer and Health cohort	Denmark	CVD (Stroke)	2,253/55,016	56	25.5	48	Median 15	FFQ	EAT-Lancet diet, comparing extreme levels (11-14 vs. 0-7)	Incident cases of stroke were identified by linkage of each participant's civil registration number to the Danish National Patient Registry	Age, sex, date of inclusion and age at inclusion, education, smoking status, physical activity, alcohol intake, and hormone replacement therapy.
Kouvari et al (29), 2022	ATTICA	Greece	CVD (Total)	317/2,020	40	25.7	55	Median 8.4	FFQ	PDI, hPDI, uPDI, comparing extreme tertiles	A CVD event was defined according to the ICD-10 criteria, as the development of acute myocardial infarction, or unstable angina, or other identified forms of ischemia (410–414.9, 427.2, 427.6), or heart failure of different types and chronic	Age, sex, educational level, smoking habits, physical activity, body mass index, family history of CVD, personal history of diabetes mellitus, hypercholesterolemia and hypertension, alcohol consumption, energy intake.

Source	Study name	Region	Disease outcome	Case/ total number	Mean age (year)	Mean BMI (kg/m^2)	Men (%)	Follow up (year)	Diet assessment	Exposure	Disease ascertainment	Model adjustment
											arrhythmias (400.0–404.9, 427.0–427.5, 427.9-) or stroke (430–438).	
Lazarova et al (30), 2022	Canadian Community Health Survey (CCHS) - Nutrition	Canada	CVD	748/6,771	Rranges between 45-80	NA	NA	Maximum 13	24-hour dietary recall	Revised PDI, hPDI, uPDI, comparing extreme quintiles	Record linkage to Canadian Vital Statistics-Death Database and Discharge Abstract Database, classified according to ICD-10	Age, day of the week on which 24-h dietary recall was collected, sequence of dietary recall, education, smoking, misreporting, physical activity, marital status, immigrant, and alcohol consumption
Zhang et al (31), 2023 (b)	Malmö Diet and Cancer (MDC) study	Sweden	CVD (CHD)	3,031/32,877	57.9	25.6	37.5	Median 24.9	FFQ	EAT-Lancet diet, comparing extreme levels (≥23 vs. ≤13)	Coronary events (including fatal and nonfatal myocardial infarction or death due to ischemic heart disease) were extracted from the Hospital Discharge Registers and cause of death register, using the ICD-9, of 410–414.	Age, sex, dietary assessment version, season, total energy intake, leisure-time physical activity, alcohol consumption, smoking status, educational level, BMI.
Thompson et al (32), 2023	UK Biobank	UK	Mortality (Total)<br>Mortality (CVD)<br>Mortality (Cancer) CVD (Total) CVD (MI)<br>CVD (Ischemic stroke)<br>CVD (Hemorrhagic stroke)<br>Cancer (Total)<br>Cancer (Prostate)<br>Cancer (Colorectal)<br>Cancer (Breast)	5627/126,217<br>698/126,217<br>3275/126,217<br>6,890/126,217<br>3,253/126,217<br>1,151/126,217<br>469/126,217<br>8,939/126,217<br>2,137/126,217<br>959/126,217<br>1,083/126,217	56.1	26.7	44.1	10.6-12.2	FFQ	hPDI, uPDI, comparing extreme quartiles	Data on mortality were available from the National Health Service death registries. CVD end points data were available from the Hospital Episode Statistics for England, Scottish Morbidity Records, and the Patient Episode Database for Wales. Cancer diagnosis data were provided through record linkage to National Cancer Registries in England, Wales, and Scotland.	Age, sex, body mass index, race and ethnicity, physical activity level, smoking status, alcohol intake, education level, energy intake, polypharmacy index, multimorbidity index, and aspirin use, stratified by region, prevalent CVD and prevalent cancer
Weston et al (33), 2022	Jackson Heart Study (JHS)	United States	CVD<br>Mortality (Total)	293/3,635<br>597/3,635	54.5	31.8	36	Median 13-15 years	FFQ	PDI, hPDI, uPDI, comparing extreme tertiles	Phone interview, hospitalizations surveillance, and death certificates reviewed	Age, sex, total energy intake, educational attainment, smoking status, alcohol intake, margarine intake, physical activity, BMI, total cholesterol, hypertension, diabetes,

Source	Study name	Region	Disease outcome	Case/ total number	Mean age (year)	Mean BMI (kg/m^2)	Men (%)	Follow up (year)	Diet assessment	Exposure	Disease ascertainment	Model adjustment
											by medical professionals	estimated glomerular filtration rate, hormone replacement therapy medication use, and statin use.
Berkel and de Waard (34), 1983	Seventh-Day Adventists in the Netherlands	The Netherlands	Mortality (Total)<br>Mortality (Cancer)<br>Mortality (CVD)	482/ 3,217<br>227/ 3,217<br>113/ 3,217	NA	NA	33.0	10	NA	Vegetarian vs. general Dutch population	Church records and linkage to the Central Bureau of Statistics	Age
Ogata et al (35), 1984	Japanese male Zen priests study	Japan	Mortality (Total)	1,396/ 4,352	≥20	NA	100	Maximum 23.0	NA	Vegetarians vs. general Japanese male	Asking offices of municipalities whether they are still alive	Age, sex, calendar year (5-year intervals) specific person-years at risk
Thorogood et al (36), 1994	Non-meat eaters and meat eaters in the United Kingdom	United Kingdom	Mortality (Total)<br>Mortality (Cancer)<br>Mortality (IHD)	404/ 11,130<br>164/ 11,130<br>94/ 11,130	39.0	BMI ≥24.1 20%	39.0	12.0	FFQ	Non-meat eaters vs. meat eaters	Record linkage with National Health Service central register, death certificates for those who subsequently died were obtained	Social class, smoking, and BMI
Key et al (37), 1996	Vegetarian and health-conscious people in the United Kingdom	United Kingdom	Mortality (Total)	1,343/ 10,771	45.8	NA	40.3	Mean 16.8	FFQ	Vegetarians vs. general United Kingdom population	Obtaining death certificates during follow-up	Age
Key et al (38), 1999	AMS	United States	Mortality (Total)<br>Mortality (IHD)<br>Mortality (Cancer)	1,635/ 24,538<br>598/ 24,538<br>118/ 24,538	51.0	24.9	36.7	Mean 5.6	FFQ	Vegetarian vs. nonvegetarian	Record linkage and personal contact	Age, sex, and smoking status
Key et al (38), 1999	AHS	United States	Mortality (Total)<br>Mortality (IHD)<br>Mortality (Cancer)	3,564/ 28,952<br>921/ 28,952<br>298/ 28,952	52.2	24.6	42.2	Mean 11.1	FFQ	Vegetarian vs. nonvegetarian	Record linkage with the California death certificate file, the National Death Index, and church records	Age, sex, and smoking status
Key et al (38), 1999	The Heidelberg Study cohort	Germany	Mortality (Total)<br>Mortality (IHD)<br>Mortality (Cancer)	185/ 1,757<br>29/ 1,757<br>23/ 1,757	48.0	21.3	44.6	Mean 9.9	FFQ	Vegetarian vs. nonvegetarian	Registrar's office of the last place of residence	Age, sex, and smoking status
Appleby et al (39), 2001	Health Food Shoppers Study	United Kingdom	Mortality (Total)<br>Mortality (Cancer)	2,346/ 10,736<br>637/	45.4	NA	40.2	Mean 18.7	FFQ	Vegetarian vs. nonvegetarian	Record linkage with the National Health	Age, sex, smoking

Source	Study name	Region	Disease outcome	Case/ total number	Mean age (year)	Mean BMI (kg/m^2)	Men (%)	Follow up (year)	Diet assessment	Exposure	Disease ascertainment	Model adjustment
			Mortality (IHD)	10,736<br>562/<br>10,736							Service Central Register	
Appleby et al (39), 2001	Oxford Vegetarian Study	United Kingdom	Mortality (Total)<br>Mortality (Cancer)<br>Mortality (IHD)	1,131/<br>11,045<br>367/<br>11,045<br>250/<br>11,045	33.2	21.7	37.4	Mean 17.6	FFQ	Vegetarian vs. nonvegetarian	Record linkage with the National Health Service Central Register	Age, sex, smoking
Chang-Claude et al (40), 2005	The German Vegetarian Study	Germany	Mortality (Total)<br>Mortality (Cancer)<br>Mortality (IHD)	456/<br>1,904<br>107/<br>1,904<br>60/<br>1,904	~45	20.9	45.1	21.0	FFQ	Vegetarian vs. nonvegetarian	The vital status of the study participants was requested from the Registrar's Office at the last documented place of residence	Age, gender, smoking, activity, alcohol consumption, BMI, and education
Bamia et al (41), 2007	EPIC-Elderly Study	10 European countries	Mortality (Total)	4,047/<br>74,607	≥60	NA	32.9	Maximum 11	FFQ	Plant-based dietary score comparing extreme tertiles	Record linkage with population mortality registries and active follow-up	Age, sex, diagnosis of diabetes mellitus at baseline, waist-to-hip ratio, BMI, educational achievement, smoking status, physical activity at current work, physical activity score at leisure time, ethanol intake and total energy intake
Key et al (42), 2009	EPIC-Oxford	United Kingdom	Mortality (Total)	1,513/<br>47,254	42.6	22.9	24.0	Maximum 14.0	FFQ	Vegetarian vs. meat eater	Record linkage with the United Kingdom's National Health Service Central Register	Age, sex, smoking, and alcohol consumption
Key et al (42), 2009	EPIC-Oxford	United Kingdom	Mortality (IHD)	213/<br>47,254	42.6	22.9	24.0	Maximum 14.0	FFQ	Vegetarian vs. meat eater	Record linkage with the United Kingdom's National Health Service Central Register	Age, sex, smoking, and alcohol consumption
Orlich et al (43), 2013	AHS-2	United States and Canada	Mortality (Total)<br>Mortality (Cancer)<br>Mortality (CVD)	2,570/<br>73,308<br>706/<br>73,308<br>987/<br>73,308	56.9	27.1	33.6	Mean 5.79	FFQ	Vegan, lacto-ovo-vegetarian, pesco-vegetarian, semi-vegetarian vs. nonvegetarian	Record linkage to National Death Index	Age, race, smoking, exercise, personal income, educational level, marital status, alcohol, region, and sleep
Martínez-González et al (44), 2014	Prevención con Dieta Mediterránea Study (PREDIMED)	Spain	Mortality (Total)<br>Mortality (Cancer)<br>Mortality (CVD)	323/<br>7,216<br>130/<br>7,216<br>76/<br>7,216	67.0	30.0	43.0	Median 4.8	FFQ	Provegetarian food pattern comparing extreme categories	Five physicians and one epidemiologist ascertained deaths from clinical registers on the basis of clinical records and death certificates	Sex, age, intervention group, smoking, leisure-time physical activity, total energy intake, educational level, and alcohol consumption

Source	Study name	Region	Disease outcome	Case/ total number	Mean age (year)	Mean BMI (kg/m^2)	Men (%)	Follow up (year)	Diet assessment	Exposure	Disease ascertainment	Model adjustment
Mihrshahi et al (45), 2016	The 45 and Up Study	Australia	Mortality (Total)	16,836/ 243,096	62.3	BMI≥30 kg/m^2; 22.2%	46.7	Mean 6.1	FFQ	Vegetarian, pesco-vegetarian, semi-vegetarian vs. regular meat eater	Record linkage to the New South Wales Registry of Births, Deaths, and Marriages	Age, sex, education level, marital status, remoteness, country of birth and Socio-Economic Indexes for Areas, smoking status, physical activity and alcohol, cancer, hypertension, and cardiovascular and metabolic disease
Kim et al (46), 2018	NHANES III	United States	Mortality (Total)<br>Mortality (CVD)	2,228/ 11,879<br>543/ 11,879	40.9	BMI ≥30 kg/m^2; 18.5%	47.3	Median 19.0	FFQ	PDI, hPDI, uPDI per 10-unit increment, converted to comparing extreme quintiles	The National Center for Health Statistics tracked survey participants' vital status and cause of death with the use of probabilistic matching and by matching their records with the National Death Index records	Race, sex, age, total energy intake, education, federal poverty level, marital status, smoking status, physical activity, alcohol consumption, margarine intake, BMI, baseline hypertension, serum cholesterol, eGFR, and menopause (for women)
Baden et al (47), 2019	NHS	United States	Mortality (Total)<br>Mortality (CVD)<br>Mortality (Cancer)	10,686/ 49,407<br>2,046/ 49,407<br>3,091/ 49,407	63.5	24.1	0	Maximum 16	FFQ	12-year change in PDI, hPDI, uPDI, comparing large increase (>10%) vs. no change for total mortality; 12-year change in PDI, hPDI, uPDI per 10 point increment for CVD mortality and cancer mortality and converted to comparing extreme quintiles (Change in adherence of plant-based diet)	Linkage with state vital statistics records and the National Death Index, or were reported by the participants' families and the U.S. postal system	Age, initial plant-based diet index score, race, family history of myocardial infarction, diabetes, or cancer, aspirin use, multivitamins use, initial BMI, menopausal status and hormone use, smoking status, smoking, physical activity, total energy intake, alcohol consumption, margarine intake, weight change, history of hypertension, hypercholesterolemia, or type 2 diabetes, antihypertensive medication use, and cholesterol-lowering medication use.
Baden et al (47), 2019	HPFS	United States	Mortality (Total)<br>Mortality (CVD)<br>Mortality (Cancer)	6,490/ 25,907<br>1,872/ 25,907<br>1,772/ 25,907	62.5	25.1	100	Maximum 16	FFQ	12-year change in PDI, hPDI, uPDI, comparing large increase (>10%) vs. no change (Change in adherence of plant-based diet)	Linkage with state vital statistics records and the National Death Index, or were reported by the participants' families and the U.S. postal system	Age, initial plant-based diet index score, race, family history of myocardial infarction, diabetes, or cancer, aspirin use, multivitamins use, initial BMI, smoking status, smoking, physical activity, total energy intake, alcohol consumption, margarine intake,

Source	Study name	Region	Disease outcome	Case/ total number	Mean age (year)	Mean BMI (kg/m^2)	Men (%)	Follow up (year)	Diet assessment	Exposure	Disease ascertainment	Model adjustment
												weight change, history of hypertension, hypercholesterolemia, or type 2 diabetes, antihypertensive medication use, and cholesterol-lowering medication use.
Kim et al (19), 2019	ARIC	United States	Mortality (Total) Mortality (CVD)	5,436/ 12,168 1,565/ 12,168	53.8	Obesity (20.7%)	44.1	Median 25	FFQ	PDI, hPDI, uPDI, provegetarian diet index comparing extreme quintiles	Ascertained through annual telephone calls with participants or proxies, active surveillance of local hospital discharge records and state death records, and linkage to the National Death Index	Age, sex, race-center, total energy intake, education, smoking status, physical activity, alcohol consumption, and margarine consumption
Anyene et al (48), 2021	The Pathways Study	United States	Mortality (Total) Mortality (Breast cancer)	653/ 3,646 323/ 3,646	60.0	28.0	0	Median 9.51	FFQ	PDI, hPDI, uPDI per 10-unit increment, and converted to comparing extreme quintiles	A combination of follow-up health status questionnaires and Kaiser Permanente Northern California electronic medical record searches	Age at diagnosis, total energy intake, physical activity, race/ethnicity, education, menopausal status, and smoking status
Kim et al (49), 2021	Korean Genome and Epidemiology Study_Health Examinees	South Korea	Mortality (Total) Mortality (CVD) Mortality (Cancer)	3,074/118,577 (Total) 447/118,577 (CVD) 1,515/118,577 (Cancer)	52.7	23.9	34.9	Maximu m 12	FFQ	PDI, hPDI, uPDI, comparing extreme quintiles	Deaths were ascertained through the death certificate database of the National Statistical Office from baseline to December 31, 2019, classified using ICD-10.	Age, sex, education, smoking status, alcohol consumption, energy intake, physical activity, body mass index, and disease history
Lo et al (50), 2021	Mr. OS and Ms. OS Study	Hong Kong	Mortality (Total) Mortality (CVD) Mortality (Cancer)	1,370/3,991 (Total) 314/3,991 (CVD) 469/3,991 (Cancer)	72.5	23.7	50	Median 11.1	FFQ	Portfolio Diet comparing extreme quartiles	Death Registry of the Department of Health of HK, classified according to ICD-10	Sex, age, dietary energy, body mass index, physical activity, systolic blood pressure, medical history (diabetes, hypertension, stroke, heart attack, angina, congestive heart failure or cancer), smoking habit, alcohol drinking, education level.
Ratjen et al (51), 2021	The biobank popgen	German y	Mortality (Total)	204/ 1,404	69.0	26.2	56	Median 7.0	FFQ	PDI, hPDI, uPDI, comparing extreme quartiles	Record linkage with the population registries	Sex, age at diet assessment, BMI, physical activity, survival time from colorectal cancer diagnosis until diet assessment, tumor location, metastases, other cancer, type of therapy, smoking status,

Source	Study name	Region	Disease outcome	Case/ total number	Mean age (year)	Mean BMI (kg/m^2)	Men (%)	Follow up (year)	Diet assessment	Exposure	Disease ascertainment	Model adjustment
												alcohol intake, total energy intake, time × age, time × BMI, and time × metastases
Petermann-Rocha et al (25), 2021	UK Biobank	United Kingdo m	Mortality (CVD)	6,580/ 422,791	56.4	27.3	44.6	Median 9.3	24-hour dietary recall	Vegetarians, fish eaters, fish, and poultry eaters vs. meat-eaters	Record linkage to registries	Age, sex, deprivation, ethnicity, comorbidities, smoking, alcohol intake, total sedentary time, physical activity, and BMI
Chen et al (52), 2022 (c)	Chinese Longitudinal Healthy Longevity Survey (CLHLS)	China	Mortality (Total)	8,937/13,154	86.9	20.3	42.6	5.7	FFQ	PDI, hPDI, uPDI, comparing extreme quintiles	Information on the death status and indicators of the predeath health status of participants was collected via interviews with their close family members. Date of death was documented according to official death certificate if available, or otherwise, from the close relatives of the participant or residents committee.	Age, sex, ethnicity, residential area, marital status, household income, education, smoking, alcohol intake, physical activity, and BMI
Delgado-Velandia et al (53), 2022	Nutrition and Cardiovascular Risk in Spain (ENRICA)	Spain	Mortality (Total) Mortality (CVD)	699/11,825 (Total) 157/11,825 (CVD)	46.9	26.9	49.6	Median 10.9 (Total), Median 9.8 (CVD)	Electronic diet history	hPDI, uPDI, comparing extreme quintiles	Record linkage to the National Death Index of Spain, classified according to ICD-10	Age, sex, education, smoking, BMI, energy intake, alcohol consumption, physical activity, number of chronic diseases, and number of medications taken
Li et al (54), 2022	NHANES	United States	Mortality (Total) Mortality (Cancer) Mortality (CVD)	4,904/ 40,074 1,068/ 40,074 1,029/ 40,074	47.3	28.5	48.0	Median 7.8	24-hour dietary recall	PDI, hPDI, uPDI, comparing extreme quintiles	Record linkage to the National Death Index	Sex, age, total energy intake, race/ethnicity, education, marital status, ratio of family income to poverty, physical activity, smoking, drinking, BMI, diabetes, hypertension, other CVDs, and cancer
Wang et al (55), 2022	The VA Million Veteran Program	United States	Mortality (Total) Mortality (CVD) Mortality (Cancer)	31,136/315,91 9 (Total) 9,751/315,919 (CVD) 9,510/315,919 (Cancer)	65.5	28.7	65.7	Mean 4	SFFQ	PDI, hPDI, uPDI, comparing extreme deciles	Record linkage to the National Death Index, classified according to ICD-10-CM	Age, sex, race, education, income, marriage, smoking, alcohol consumption, frequency of exercise vigorously, total energy intake, BMI, histories of diabetes, hypertension, hypercholesterolemia, cancer and CVD at baseline

Source	Study name	Region	Disease outcome	Case/ total number	Mean age (year)	Mean BMI (kg/m^2)	Men (%)	Follow up (year)	Diet assessment	Exposure	Disease ascertainment	Model adjustment
Shan et al (56), 2023	NHS	United States	Mortality (Total)<br>Mortality (CVD)<br>Mortality (Heart disease)<br>Mortality (Stroke)<br>Mortality (Cancer)<br>Mortality (Respiratory disease)<br>Mortality (Neurodegenerative disease)	Total: 31,263/75,230<br>CVD: 6,128/75,230<br>Heart disease: 4,330/75,230<br>Stroke: 1,798/75,230<br>Cancer: 8,733/75,230<br>Respiratory disease: 2,491/75,230<br>Neurodegenerative disease: 5,004/75,230	50.2	24.9	0	Maximum 36	FFQ	hPDI, comparing extreme quintiles	Self-reported, confirmed by medical records	Age, calendar year, race, marriage status, living status, family history of MI, family history of diabetes, family history of cancer, menopaUnited Statesl status (in women), multivitamin use, aspirin use, total energy intake, smoking, alcohol consumption, history of hypertension, history of hypercholesterolemia, and BMI
Shan et al (56), 2023	HPFS	United States	Mortality (Total)<br>Mortality (CVD)<br>Mortality (Heart disease)<br>Mortality (Stroke)<br>Mortality (Cancer)<br>Mortality (Respiratory disease)<br>Mortality (Neurodegenerative disease)	Total: 22,900/44,085<br>CVD: 6,641/44,085<br>Heart disease: 5,386/44,085<br>Stroke: 1,255/44,085<br>Cancer: 5,710/44,085<br>Respiratory disease: 1,738/44,085<br>Neurodegenerative disease: 2,101/44,085	53.3	24.0	100	Maximum 36	FFQ	hPDI, comparing extreme quintiles	Self-reported, confirmed by medical records	Age, calendar year, race, marriage status, living status, family history of MI, family history of diabetes, family history of cancer, multivitamin use, aspirin use, total energy intake, smoking, alcohol consumption, history of hypertension, history of hypercholesterolemia, and BMI
Fraser et al (57), 1999	AHS	United States	Cancer (Breast)<br>Cancer (Colon)<br>Cancer (Lung)<br>Cancer (Prostate)<br>Cancer (Uterine)	128/ 34,198<br>107/ 34,198<br>45/ 34,198<br>127/ 34,198<br>116/ 34,198	54.0	25.0	40.5	6	FFQ	Vegetarian vs. Nonvegetarian	Record linkage to population-based tumor registries, state death tapes and the National Death Index	Age, sex, and smoking (for lung cancer)
Key et al (58), 2009	EPIC-Oxford	United Kingdom	Cancer (Total)<br>Cancer (Breast)<br>Cancer (Colorectal)	2,179/ 63,550<br>734/ 63,550	43.4	23.0	23.2	Maximum 12.0	FFQ	Vegetarian vs. Nonvegetarian	Record linkage with the United Kingdom's National Health	Smoking

Source	Study name	Region	Disease outcome	Case/ total number	Mean age (year)	Mean BMI (kg/m^2)	Men (%)	Follow up (year)	Diet assessment	Exposure	Disease ascertainment	Model adjustment
			Cancer (Lung)<br>Cancer (Ovarian)<br>Cancer (Prostate)	228/ 63,550<br>88/ 63,550<br>92/ 63,550<br>183/ 63,550							Service Central Register	
Cade et al (59), 2010	UKWCS	United Kingdo m	Cancer (Breast)	783/ 35,372	52.6	24.5	0	Mean 9.0	FFQ	Vegetarian, fish eater, poultry eater, vs. red meat eater	Subjects were flagged with the National Health Service Central Register for cancer and death notification	Age, energy intake, menopausal status, calorie adjusted fat, BMI, physical activity, oral contraception pill use, hormone replacement therapy use, smoking status, parity, age at menarche, ethanol, total days breast feeding, socioeconomic class, and level of education
Tantamango-Bartley et al (60), 2013	AHS-2	United States and Canada	Cancer (Total)	2,939/ 69,120	≥30	27.2	36.3	4.14	FFQ	Vegan, lactoovo-vegetarian, pesco-vegetarian, semi-vegetarian and non-vegetarian	Record linkage with state tumor registries	Race, family history of cancer, BMI, education, smoking, alcohol, age at menarche, pregnancies, breastfeeding, oral contraceptives, hormone replacement therapy, and menopause status
Gilsing et al (61), 2015	NLCS-MIC	The Netherla nds	Cancer (Colorectal)	437/ 10,210	61.3	24.7	53.5	20.3	FFQ	Vegetarians, pescetarians, 1 day/week meat consumers, 2-5 day/week meat consumers vs. 6-7 day/week meat consumers	Repeated record linkage to the Netherlands Cancer Registry, the Dutch Pathology Registry, and the cause of death registry (Statistics Netherlands)	Age, sex, total energy intake, cigarette smoking, alcohol consumption, BMI, non-occupational physical activity, and level of education
Orlich et al (62), 2015	AHS-2	United States and Canada	Cancer (Colorectal)	490/ 77,659	57.1	27.2	34.5	Mean 7.3	FFQ	Vegan, lacto-ovo-vegetarian, pesco-vegetarian, semi-vegetarian vs. Nonvegetarian	Record linkage with state cancer registries	Age, race, sex, education, moderate or vigorous exercise, smoking, alcohol use, family history of colorectal cancer, history of peptic ulcer, history of inflammatory bowel disease, treatment for diabetes mellitus within the past year, used aspirin at least weekly at least 2 of the past 5 years, used statins at least 2 of the past 5 years, prior colonoscopy or flexible sigmoidoscopy, supplemental calcium use, supplemental vitamin D, dietary energy, and hormone therapy among menopausal women, BMI, and fiber intake

Source	Study name	Region	Disease outcome	Case/ total number	Mean age (year)	Mean BMI (kg/m^2)	Men (%)	Follow up (year)	Diet assessment	Exposure	Disease ascertainment	Model adjustment
Gilsing et al (63), 2016	NLCS-MIC	The Netherlands	Cancer (Breast)	312/ 5,218	61.3	24.7	0	20.3	FFQ	Vegetarian vs. nonvegetarian	Repeated record linkage to the Netherlands Cancer Registry, the Dutch Pathology Registry, and the cause of death registry (Statistics Netherlands)	Age, total energy intake, cigarette smoking, frequency of smoking, duration of smoking, alcohol consumption, BMI, non-occupational physical activity, and level of education
Gilsing et al (63), 2016	NLCS-MIC	The Netherlands	Cancer (Lung)	279/ 9,773	61.3	24.7	45.0	20.3	FFQ	Vegetarian vs. nonvegetarian	Repeated record linkage to the Netherlands Cancer Registry, the Dutch Pathology Registry, and the cause of death registry (Statistics Netherlands)	Age, total energy intake, cigarette smoking, frequency of smoking, duration of smoking, alcohol consumption, BMI, non-occupational physical activity, and level of education
Gilsing et al (63), 2016	NLCS-MIC	The Netherlands	Cancer (Prostate)	399/ 4,864	61.3	24.7	100	20.3	FFQ	Vegetarian vs. nonvegetarian	Repeated record linkage to the Netherlands Cancer Registry, the Dutch Pathology Registry, and the cause of death registry (Statistics Netherlands)	Age, total energy intake, cigarette smoking, frequency of smoking, duration of smoking, alcohol consumption, BMI, non-occupational physical activity, and level of education
Tantamango-Bartley et al (64), 2016	AHS-2	United States and Canada	Cancer (Prostate)	1,079/ 27,188	66.0	BMI>30 kg/m^2; 20%	100	Mean 7.8	FFQ	Vegan, lacto-ovo-vegetarian, pesco-vegetarian, semi-vegetarian vs. nonvegetarian	Linkage to state cancer registries	Age, race, family history of prostate cancer, education, screening for prostate cancer, total calorie, and BMI
Penniecook-Sawyers et al (65), 2016	AHS-2	United States and Canada	Cancer (Breast)	892/ 50,404	35-110	27.5	0	Mean 7.8	FFQ	Vegan, lactoovo-vegetarian, pesco-vegetarian, semi-vegetarian and non-vegetarian	Record linkage with forty-eight state cancer registries	Race, height, physical activity, family history of cancer, mammography in the last 2 years after age 42 years, age at menopause, age at menarche, birth control pills, hormone replacement therapy, age at first child, number of children, breastfeeding, educational level, smoking, alcohol, and BMI
Rada-Fernandez de Jauregui et al (66), 2018	UKWCS	United Kingdom	Cancer (Colorectal)	462/ 32,147	52.0	24.4	0	Mean 17.2	FFQ	Red meat free eaters vs. red meat eaters	Record linkage of cancer identification codes from the central register of National Health Service	Age, BMI, energy intake, physical activity, smoking status, family history of CRC in a first degree relative and socio-economic status

Source	Study name	Region	Disease outcome	Case/ total number	Mean age (year)	Mean BMI (kg/m^2)	Men (%)	Follow up (year)	Diet assessment	Exposure	Disease ascertainment	Model adjustment
Kane-Diallo et al (67), 2018	The NutriNet-Santé study	France	Cancer (Breast)<br>Cancer (Digestive)<br>Cancer (Lung)<br>Cancer (Prostate)<br>Cancer (Total)	487/ 42,544<br>198/ 42,544<br>68/ 42,544<br>243/ 42,544<br>1,591/ 42,544	56.9	24.7	27.3	Median 4.3	24-hour dietary recall	Pro plant-based dietary score comparing extreme tertiles	Self-reported, confirmed by medical records	Age, sex, energy intake without alcohol, number of 24-hr dietary records, smoking status, educational level, physical activity, height, BMI, alcohol intake, family history of cancer, lipids intake, and for breast cancer analyses, hormone replacement therapy, number of children, and contraception use
Leone et al (68), 2020	SUN	Spain	Cancer (Skin, basal cell carcinoma)	101/ 505	48.0	44.6	24.0	Maximum 20.0	FFQ	Pro-vegetarian dietary pattern comparing extreme quintiles	Self-reported confirmation of medical records	Age, height, smoking, physical activity, recruitment year, total energy intake, family history of melanoma, use of sunscreen during sun exposure, sunburns during childhood and adolescence, number of sunburns during adolescence, and presence of freckles
Romanos-Nanclares et al (69), 2020	SUN	Spain	Cancer (Breast)	101/ 10,812	34.6	22.2	0	Median 11.5	FFQ	Provegetarian food pattern, healthful provegetarian food pattern, and unhealthful provegetarian food pattern comparing extreme tertiles	Self-reported with confirmation of follow-up questionnaire and medical records	Height, family history of breast cancer, smoking status, physical activity, alcohol intake, BMI, age at the time of menarche, menopause, number of pregnancies >6 months, pregnancy before the age of 30 years, months of breastfeeding, use of hormone replacement therapy and its duration, years at university, and total energy intake
Anyene et al (48), 2021	The Pathways Study	United States	Breast cancer recurrence	461/ 3,646	60.0	28.0	0	Median 9.2	FFQ	PDI, hPDI, uPDI per 10-unit increment	A combination of follow-up health status questionnaires and Kaiser Permanente Northern California electronic medical record searches	Age at diagnosis, total energy intake, physical activity, race/ethnicity, education, menopausal status, and smoking status
Romanos-Nanclares et al (70), 2021	NHS	United States	Cancer (Breast)	8,220/ 76,690	50.9	25.1	0	Maximum 32.0	FFQ	PDI, hPDI, uPDI, comparing extreme quintiles	Self-reported, confirmed by medical records	Race, age at menarche, age at menopause, postmenopausal hormone use, oral contraceptive use history, parity and age at first birth, breastfeeding history, family history of breast cancer and benign breast disease, height, alcohol intake, total caloric intake, physical activity, BMI at age 18 years and socioeconomic status

Source	Study name	Region	Disease outcome	Case/ total number	Mean age (year)	Mean BMI (kg/m^2)	Men (%)	Follow up (year)	Diet assessment	Exposure	Disease ascertainment	Model adjustment
Romanos-Nanclares et al (70), 2021	NHSII	United States	Cancer (Breast)	4,262/ 93,295	36.7	24.4	0	Maximum 26.0	FFQ	PDI, hPDI, uPDI, comparing extreme quintiles	Self-reported, confirmed by medical records	Race, age at menarche, age at menopause, postmenopausal hormone use, oral contraceptive use history, parity and age at first birth, breastfeeding history, family history of breast cancer and benign breast disease, height, alcohol intake, total caloric intake, physical activity, BMI at age 18 years and socioeconomic status
Kim et al (71), 2022	Multiethnic Cohort Study	United States	Cancer (Colorectal)	2,582/79,952 (Men) 2,394/93,475 (Women)	60.0 (Men) 59.3 (Women)	26.6 (Men) 26.4 (Women)	46.1	Mean 19.2	QFFQ	PDI, hPDI, uPDI, comparing extreme quintiles	Incident colorectal cancer cases were identified by linkage to the statewide Surveillance, Epidemiology, and End Results Program tumor registries in Hawaii and California. Deaths were identified by linkage to death certificate files in both states and the National Death Index.	Age at cohort entry, family history of colorectal cancer, history of colorectal polyp, BMI, smoking, multivitamin use, nonsteroidal anti-inflammatory drug use, physical activity, menopausal hormone therapy use for women only, alcohol consumption, and total energy intake
Loeb et al (72), 2022	HPFS	United States	Cancer (Prostate)	6,655/47,239	65	22	100	Median 20.7	FFQ	PDI, hPDI, comparing extreme quintiles	Biennial questionnaires, medical records and pathology reports.	Age and time period, race, height, BMI, BMI at age 21, smoking status, family history of prostate cancer, PSA test in previous cycle, PSA testing in >50% of previous cycles, multivitamin use, vitamin E supplement use, alcohol intake, physical activity, aspirin use, anti-cholesterol medication, diabetes, total energy intake.
Kim et al (73), 2023 (a)	Multiethnic Cohort Study	United States	Cancer (Hepatocellular carcinoma)	772/170,321	59.5	26.5	46.3	Mean 19.6	FFQ	PDI, hPDI, uPDI, comparing extreme quintiles	Incident HCC cases were ascertained by linkage to the statewide Surveillance, Epidemiology and End Results Program tumor registries in Hawaii and California.	Race and ethnicity, sex, age at cohort entry, family history of liver cancer, history of diabetes, BMI, cigarette smoking, alcohol consumption, and total energy intake.
Kim et al (74), 2023 (b)	NHS	United States	Cancer (Digestive system)	Digestive system 3,178/74,496	65.0	25.5	0	Maximum 34	FFQ	PDI, hPDI, uPDI, per 10 points increment	Self-reported, confirmed by medical records	Age, calendar year, cohort, race, BMI, physical activity, smoking, alcohol consumption, family history of cancer, personal history

Source	Study name	Region	Disease outcome	Case/ total number	Mean age (year)	Mean BMI (kg/m^2)	Men (%)	Follow up (year)	Diet assessment	Exposure	Disease ascertainment	Model adjustment
			Cancer (Colorectal) Cancer (Pancreatic) Cancer (Liver) Cancer (Stomach)	Colorectal: 1,883/74,496 Pancreatic: 554/74,496 Liver: 86/74,496 Stomach: 136/74,496								of diabetes, current multivitamin use, regular aspirin use, regular NSAID use, menopaUnited Statesl status (in women), total energy intake, and calcium supplement intake. For liver and stomach cancer, the models were adjusted for age only.
Kim et al (74), 2023 (b)	NHSII	United States	Cancer (Digestive system) Cancer (Colorectal) Cancer (Pancreatic) Cancer (Liver) Cancer (Stomach)	Digestive system: 714/91,705 Colorectal: 464/91,705 Pancreatic: 78/91,705 Liver: 15/91,705 Stomach: 14/91,705	49.3	25.5	0	Maximum 26	FFQ	PDI, hPDI, uPDI, per 10 points increment	Self-reported, confirmed by medical records	Age, calendar year, cohort, race, BMI, physical activity, smoking, alcohol consumption, family history of cancer, personal history of diabetes, current multivitamin use, regular aspirin use, regular NSAID use, menopausal status (in women), total energy intake, and calcium supplement intake. For liver and stomach cancer, the models were adjusted for age only.
Kim et al (74), 2023 (b)	HPFS	United States	Cancer (Digestive system) Cancer (Colorectal) Cancer (Pancreatic) Cancer (Liver) Cancer (Stomach)	Digestive system: 2,626/45,472 Colorectal: 1,447/45,472 Pancreatic: 494/45,472 Liver: 74/45,472 Stomach: 169/45,472	65.4	25.5	100	Maximum 30	FFQ	PDI, hPDI, uPDI, per 10 points increment	Self-reported, confirmed by medical records	Age, calendar year, cohort, race, BMI, physical activity, smoking, alcohol consumption, family history of cancer, personal history of diabetes, current multivitamin use, regular aspirin use, regular NSAID use, total energy intake, and calcium supplement intake. For liver and stomach cancer, the models were adjusted for age only.
Shah et al (75), 2022	E3N	France	Cancer (Breast)	3,968/65,574	52.9	22.9	0	Mean 21	FFQ	PDI, hPDI, uPDI, comparing extreme quintiles	Self-reported, confirmed through pathological reports	Age, birth cohort, education, physical activity, smoking, history of breast cancer, breastfeeding, age at menarche, age at first full-term birth, past history of benign breast disease, ever use of the contraceptive pill, ever use of menopausal hormone therapy, mammography in the last follow-up cycle, BMI, energy intake, and alcohol consumption
Zhong et al (76), 2023	Prostate, Lung, Colorectal, and Ovarian (PLCO) Cancer Screening Trial	United States	Cancer (Pancreatic)	421/101,748	65.5	27.2	48.6	Mean 8.9	Diet history questionnaire	PDI, hPDI, uPDI, comparing extreme quartiles	Self-reported, confirmed by medical records	Age, sex, race, BMI, alcohol consumption, smoking, family history of pancreatic cancer, and history of diabetes; energy intake wasdjusted for food consumption

Source	Study name	Region	Disease outcome	Case/ total number	Mean age (year)	Mean BMI (kg/m^2)	Men (%)	Follow up (year)	Diet assessment	Exposure	Disease ascertainment	Model adjustment
												and nutrient intakes before formal analyses

Abbreviations: AHS, Adventist Health Study; AHS-2, Adventist Health Study-2; AMS, Adventist Mortality Study; APDQS, A Priori Diet Quality Score; ARIC, Atherosclerosis Risk in Communities; BMI, body mass index; CHD, coronary heart disease; CRC, colorectal cancer; CVD, cardiovascular disease; eGFR, estimated glomerular filtration rate; EPIC, European Prospective Investigation into Cancer and Nutrition; FFQ, food frequency questionnaire; HbA1c, hemoglobin A1c; hPDI, Healthful Plant-Based Diet Index; HPFS, Health Professionals Follow-up Study; IHD, ischemic heart disease; MI, myocardial infarction; NHANES, National Health and Nutrition Examination Survey; NHS, Nurses' Health Study; NHSII, Nurses' Health Study II; NLCS-MIC, Netherlands Cohort Study-Meat Investigation Cohort; PDI, Plant-Based Diet Index; SUN, Seguimiento Universidad de Navarra cohort; T2D, type 2 diabetes; UKWCS, United Kingdom Women's Cohort Study; uPDI, Unhealthful Plant-Based Diet Index; Y, year; WHI, Women's Health Initiative Prospective Cohort Study; UK, United Kingdom.

Supplemental Table S4. Assessment of Individual Study Bias and Study Quality.

Source	Criteria	Yes	No	Cannot determine, not applicable
Vang et al (1), 2008	1. Was the research question or objective in this paper clearly stated?	X		
	2. Was the study population clearly specified and defined?	X		
	3. Was the participation rate of eligible persons at least 50%?		X	
	4. Were all the subjects selected or recruited from the same or similar populations (including the same time period)? Were inclusion and exclusion criteria for being in the study prespecified and applied uniformly to all participants?	X		
	5. Was a sample size justification, power description, or variance and effect estimates provided?		X	
	6. For the analyses in this paper, were the exposure(s) of interest measured prior to the outcome(s) being measured?	X		
	7. Was the timeframe sufficient so that one could reasonably expect to see an association between exposure and outcome if it existed?	X		
	8. For exposures that can vary in amount or level, did the study examine different levels of the exposure as related to the outcome (e.g., categories of exposure, or exposure measured as continuous variable)?	X X		
	9. Were the exposure measures (independent variables) clearly defined, valid, reliable, and implemented consistently across all study participants?	X		
	10. Was the exposure(s) assessed more than once over time?		X	
	11. Were the outcome measures (dependent variables) clearly defined, valid, reliable, and implemented consistently across all study participants?		X	
	12. Were the outcome assessors blinded to the exposure status of participants?	X		
	13. Was loss to follow-up after baseline 20% or less?			X
	14. Were key potential confounding variables measured and adjusted statistically for their impact on the relationship between exposure(s) and outcome(s)?		X	
	**Total**	8		
Tonstad et al (2), 2013	1. Was the research question or objective in this paper clearly stated?	X		
	2. Was the study population clearly specified and defined?	X		
	3. Was the participation rate of eligible persons at least 50%?		X	
	4. Were all the subjects selected or recruited from the same or similar populations (including the same time period)? Were inclusion and exclusion criteria for being in the study prespecified and applied uniformly to all participants?	X		
	5. Was a sample size justification, power description, or variance and effect estimates provided?		X	
	6. For the analyses in this paper, were the exposure(s) of interest measured prior to the outcome(s) being measured?	X		
	7. Was the timeframe sufficient so that one could reasonably expect to see an association between exposure and outcome if it existed?	X		
	8. For exposures that can vary in amount or level, did the study examine different levels of the exposure as related to the outcome (e.g., categories of exposure, or exposure measured as continuous variable)?	X		
	9. Were the exposure measures (independent variables) clearly defined, valid, reliable, and implemented consistently across all study participants?	X		
	10. Was the exposure(s) assessed more than once over time?		X	
	11. Were the outcome measures (dependent variables) clearly defined, valid, reliable, and implemented consistently across all study participants?	X		
	12. Were the outcome assessors blinded to the exposure status of participants?	X		
	13. Was loss to follow-up after baseline 20% or less?			X
	14. Were key potential confounding variables measured and adjusted statistically for their impact on the relationship between exposure(s) and outcome(s)?	X		
	**Total**	10		

Source	Criteria	Yes	No	Cannot determine, not applicable
Satija et al (3), 2016 (NHS, NHSII, HPFS)	1. Was the research question or objective in this paper clearly stated?	X		
	2. Was the study population clearly specified and defined?	X		
	3. Was the participation rate of eligible persons at least 50%?	X		
	4. Were all the subjects selected or recruited from the same or similar populations (including the same time period)? Were inclusion and exclusion criteria for being in the study prespecified and applied uniformly to all participants?	X		
	5. Was a sample size justification, power description, or variance and effect estimates provided?		X	
	6. For the analyses in this paper, were the exposure(s) of interest measured prior to the outcome(s) being measured?	X		
	7. Was the timeframe sufficient so that one could reasonably expect to see an association between exposure and outcome if it existed?	X		
	8. For exposures that can vary in amount or level, did the study examine different levels of the exposure as related to the outcome (e.g., categories of exposure, or exposure measured as continuous variable)?	X		
	9. Were the exposure measures (independent variables) clearly defined, valid, reliable, and implemented consistently across all study participants?	X		
	10. Was the exposure(s) assessed more than once over time?	X		
	11. Were the outcome measures (dependent variables) clearly defined, valid, reliable, and implemented consistently across all study participants?	X		
	12. Were the outcome assessors blinded to the exposure status of participants?	X		
	13. Was loss to follow-up after baseline 20% or less?	X		
	14. Were key potential confounding variables measured and adjusted statistically for their impact on the relationship between exposure(s) and outcome(s)?	X		
	**Total**	13		
Chen et al (4), 2018 (Rotterdam Study I, II, III)	1. Was the research question or objective in this paper clearly stated?	X		
	2. Was the study population clearly specified and defined?	X		
	3. Was the participation rate of eligible persons at least 50%?		X	
	4. Were all the subjects selected or recruited from the same or similar populations (including the same time period)? Were inclusion and exclusion criteria for being in the study prespecified and applied uniformly to all participants?	X		
	5. Was a sample size justification, power description, or variance and effect estimates provided?		X	
	6. For the analyses in this paper, were the exposure(s) of interest measured prior to the outcome(s) being measured?	X		
	7. Was the timeframe sufficient so that one could reasonably expect to see an association between exposure and outcome if it existed?	X		
	8. For exposures that can vary in amount or level, did the study examine different levels of the exposure as related to the outcome (e.g., categories of exposure, or exposure measured as continuous variable)?		X	
	9. Were the exposure measures (independent variables) clearly defined, valid, reliable, and implemented consistently across all study participants?	X		
	10. Was the exposure(s) assessed more than once over time?		X	
	11. Were the outcome measures (dependent variables) clearly defined, valid, reliable, and implemented consistently across all study participants?	X		
	12. Were the outcome assessors blinded to the exposure status of participants?	X		
	13. Was loss to follow-up after baseline 20% or less?	X		
	14. Were key potential confounding variables measured and adjusted statistically for their impact on the relationship between exposure(s) and outcome(s)?	X		
	**Total**	10		
Chen et al (5), 2018	1. Was the research question or objective in this paper clearly stated?	X		
	2. Was the study population clearly specified and defined?	X		

Source	Criteria	Yes	No	Cannot determine, not applicable
(Singapore Chinese Health Study)	3. Was the participation rate of eligible persons at least 50%?	X		
	4. Were all the subjects selected or recruited from the same or similar populations (including the same time period)? Were inclusion and exclusion criteria for being in the study prespecified and applied uniformly to all participants?	X		
	5. Was a sample size justification, power description, or variance and effect estimates provided?		X	
	6. For the analyses in this paper, were the exposure(s) of interest measured prior to the outcome(s) being measured?	X		
	7. Was the timeframe sufficient so that one could reasonably expect to see an association between exposure and outcome if it existed?	X		
	8. For exposures that can vary in amount or level, did the study examine different levels of the exposure as related to the outcome (e.g., categories of exposure, or exposure measured as continuous variable)?	X		
	9. Were the exposure measures (independent variables) clearly defined, valid, reliable, and implemented consistently across all study participants?	X		
	10. Was the exposure(s) assessed more than once over time?		X	
	11. Were the outcome measures (dependent variables) clearly defined, valid, reliable, and implemented consistently across all study participants?	X		
	12. Were the outcome assessors blinded to the exposure status of participants?	X		
	13. Was loss to follow-up after baseline 20% or less?	X		
	14. Were key potential confounding variables measured and adjusted statistically for their impact on the relationship between exposure(s) and outcome(s)?	X		
	**Total**	12		
Chiu et al (6), 2018	1. Was the research question or objective in this paper clearly stated?	X		
	2. Was the study population clearly specified and defined?	X		
	3. Was the participation rate of eligible persons at least 50%?	X		
	4. Were all the subjects selected or recruited from the same or similar populations (including the same time period)? Were inclusion and exclusion criteria for being in the study prespecified and applied uniformly to all participants?	X		
	5. Was a sample size justification, power description, or variance and effect estimates provided?		X	
	6. For the analyses in this paper, were the exposure(s) of interest measured prior to the outcome(s) being measured?	X		
	7. Was the timeframe sufficient so that one could reasonably expect to see an association between exposure and outcome if it existed?	X		
	8. For exposures that can vary in amount or level, did the study examine different levels of the exposure as related to the outcome (e.g., categories of exposure, or exposure measured as continuous variable)?			X
	9. Were the exposure measures (independent variables) clearly defined, valid, reliable, and implemented consistently across all study participants?	X		
	10. Was the exposure(s) assessed more than once over time?	X		
	11. Were the outcome measures (dependent variables) clearly defined, valid, reliable, and implemented consistently across all study participants?	X		
	12. Were the outcome assessors blinded to the exposure status of participants?	X		
	13. Was loss to follow-up after baseline 20% or less?			X
	14. Were key potential confounding variables measured and adjusted statistically for their impact on the relationship between exposure(s) and outcome(s)?	X		
	**Total**	11		
Papier et al (7), 2019	1. Was the research question or objective in this paper clearly stated?	X		
	2. Was the study population clearly specified and defined?	X		
	3. Was the participation rate of eligible persons at least 50%?	X		

Source	Criteria	Yes	No	Cannot determine, not applicable
	4. Were all the subjects selected or recruited from the same or similar populations (including the same time period)? Were inclusion and exclusion criteria for being in the study prespecified and applied uniformly to all participants?	X		
	5. Was a sample size justification, power description, or variance and effect estimates provided?		X	
	6. For the analyses in this paper, were the exposure(s) of interest measured prior to the outcome(s) being measured?	X		
	7. Was the timeframe sufficient so that one could reasonably expect to see an association between exposure and outcome if it existed?	X		
	8. For exposures that can vary in amount or level, did the study examine different levels of the exposure as related to the outcome (e.g., categories of exposure, or exposure measured as continuous variable)?			X
	9. Were the exposure measures (independent variables) clearly defined, valid, reliable, and implemented consistently across all study participants?	X		
	10. Was the exposure(s) assessed more than once over time?		X	
	11. Were the outcome measures (dependent variables) clearly defined, valid, reliable, and implemented consistently across all study participants?	X		
	12. Were the outcome assessors blinded to the exposure status of participants?	X		
	13. Was loss to follow-up after baseline 20% or less?	X		
	14. Were key potential confounding variables measured and adjusted statistically for their impact on the relationship between exposure(s) and outcome(s)?	X		
	**Total**	11		
Choi et al (8), 2020	1. Was the research question or objective in this paper clearly stated?	X		
	2. Was the study population clearly specified and defined?	X		
	3. Was the participation rate of eligible persons at least 50%?		X	
	4. Were all the subjects selected or recruited from the same or similar populations (including the same time period)? Were inclusion and exclusion criteria for being in the study prespecified and applied uniformly to all participants?	X		
	5. Was a sample size justification, power description, or variance and effect estimates provided?		X	
	6. For the analyses in this paper, were the exposure(s) of interest measured prior to the outcome(s) being measured?	X		
	7. Was the timeframe sufficient so that one could reasonably expect to see an association between exposure and outcome if it existed?	X		
	8. For exposures that can vary in amount or level, did the study examine different levels of the exposure as related to the outcome (e.g., categories of exposure, or exposure measured as continuous variable)?	X		
	9. Were the exposure measures (independent variables) clearly defined, valid, reliable, and implemented consistently across all study participants?	X		
	10. Was the exposure(s) assessed more than once over time?	X		
	11. Were the outcome measures (dependent variables) clearly defined, valid, reliable, and implemented consistently across all study participants?	X		
	12. Were the outcome assessors blinded to the exposure status of participants?	X		
	13. Was loss to follow-up after baseline 20% or less?		X	
	14. Were key potential confounding variables measured and adjusted statistically for their impact on the relationship between exposure(s) and outcome(s)?	X		
	**Total**	11		
Chen et al (9), 2021 (NHS, NHSII, HPFS)	1. Was the research question or objective in this paper clearly stated?	X		
	2. Was the study population clearly specified and defined?	X		
	3. Was the participation rate of eligible persons at least 50%?	X		
	4. Were all the subjects selected or recruited from the same or similar populations (including the same time period)? Were inclusion and exclusion criteria for being in the study prespecified and applied uniformly to all participants?	X		

Source	Criteria	Yes	No	Cannot determine, not applicable
	5. Was a sample size justification, power description, or variance and effect estimates provided?		X	
	6. For the analyses in this paper, were the exposure(s) of interest measured prior to the outcome(s) being measured?	X		
	7. Was the timeframe sufficient so that one could reasonably expect to see an association between exposure and outcome if it existed?	X		
	8. For exposures that can vary in amount or level, did the study examine different levels of the exposure as related to the outcome (e.g., categories of exposure, or exposure measured as continuous variable)?	X		
	9. Were the exposure measures (independent variables) clearly defined, valid, reliable, and implemented consistently across all study participants?	X		
	10. Was the exposure(s) assessed more than once over time?	X		
	11. Were the outcome measures (dependent variables) clearly defined, valid, reliable, and implemented consistently across all study participants?	X		
	12. Were the outcome assessors blinded to the exposure status of participants?	X		
	13. Was loss to follow-up after baseline 20% or less?	X		
	14. Were key potential confounding variables measured and adjusted statistically for their impact on the relationship between exposure(s) and outcome(s)?	X		
	**Total**	13		
Flores et al (10), 2021	1. Was the research question or objective in this paper clearly stated?	X		
	2. Was the study population clearly specified and defined?	X		
	3. Was the participation rate of eligible persons at least 50%?	X		
	4. Were all the subjects selected or recruited from the same or similar populations (including the same time period)? Were inclusion and exclusion criteria for being in the study prespecified and applied uniformly to all participants?	X		
	5. Was a sample size justification, power description, or variance and effect estimates provided?		X	
	6. For the analyses in this paper, were the exposure(s) of interest measured prior to the outcome(s) being measured?	X		
	7. Was the timeframe sufficient so that one could reasonably expect to see an association between exposure and outcome if it existed?	X		
	8. For exposures that can vary in amount or level, did the study examine different levels of the exposure as related to the outcome (e.g., categories of exposure, or exposure measured as continuous variable)?	X		
	9. Were the exposure measures (independent variables) clearly defined, valid, reliable, and implemented consistently across all study participants?	X		
	10. Was the exposure(s) assessed more than once over time?		X	
	11. Were the outcome measures (dependent variables) clearly defined, valid, reliable, and implemented consistently across all study participants?	X		
	12. Were the outcome assessors blinded to the exposure status of participants?	X		
	13. Was loss to follow-up after baseline 20% or less?	X		
	14. Were key potential confounding variables measured and adjusted statistically for their impact on the relationship between exposure(s) and outcome(s)?	X		
	**Total**	12		
Laouali et al (11), 2021	1. Was the research question or objective in this paper clearly stated?	X		
	2. Was the study population clearly specified and defined?	X		
	3. Was the participation rate of eligible persons at least 50%?	X		
	4. Were all the subjects selected or recruited from the same or similar populations (including the same time period)? Were inclusion and exclusion criteria for being in the study prespecified and applied uniformly to all participants?	X		
	5. Was a sample size justification, power description, or variance and effect estimates provided?		X	
	6. For the analyses in this paper, were the exposure(s) of interest measured prior to the outcome(s) being measured?	X		

Source	Criteria	Yes	No	Cannot determine, not applicable
	7. Was the timeframe sufficient so that one could reasonably expect to see an association between exposure and outcome if it existed?	X		
	8. For exposures that can vary in amount or level, did the study examine different levels of the exposure as related to the outcome (e.g., categories of exposure, or exposure measured as continuous variable)?	X		
	9. Were the exposure measures (independent variables) clearly defined, valid, reliable, and implemented consistently across all study participants?	X		
	10. Was the exposure(s) assessed more than once over time?		X	
	11. Were the outcome measures (dependent variables) clearly defined, valid, reliable, and implemented consistently across all study participants?	X		
	12. Were the outcome assessors blinded to the exposure status of participants?	X		
	13. Was loss to follow-up after baseline 20% or less?	X		
	14. Were key potential confounding variables measured and adjusted statistically for their impact on the relationship between exposure(s) and outcome(s)?	X		
	**Total**	12		
Yang et al (12), 2021	1. Was the research question or objective in this paper clearly stated?	X		
	2. Was the study population clearly specified and defined?	X		
	3. Was the participation rate of eligible persons at least 50%?	X		
	4. Were all the subjects selected or recruited from the same or similar populations (including the same time period)? Were inclusion and exclusion criteria for being in the study prespecified and applied uniformly to all participants?	X		
	5. Was a sample size justification, power description, or variance and effect estimates provided?		X	
	6. For the analyses in this paper, were the exposure(s) of interest measured prior to the outcome(s) being measured?	X		
	7. Was the timeframe sufficient so that one could reasonably expect to see an association between exposure and outcome if it existed?	X		
	8. For exposures that can vary in amount or level, did the study examine different levels of the exposure as related to the outcome (e.g., categories of exposure, or exposure measured as continuous variable)?	X		
	9. Were the exposure measures (independent variables) clearly defined, valid, reliable, and implemented consistently across all study participants?	X		
	10. Was the exposure(s) assessed more than once over time?		X	
	11. Were the outcome measures (dependent variables) clearly defined, valid, reliable, and implemented consistently across all study participants?	X		
	12. Were the outcome assessors blinded to the exposure status of participants?	X		
	13. Was loss to follow-up after baseline 20% or less?			X
	14. Were key potential confounding variables measured and adjusted statistically for their impact on the relationship between exposure(s) and outcome(s)?	X		
	**Total**	11		
Bhupathiraju et al (13), 2022	1. Was the research question or objective in this paper clearly stated?	X		
	2. Was the study population clearly specified and defined?	X		
	3. Was the participation rate of eligible persons at least 50%?	X		
	4. Were all the subjects selected or recruited from the same or similar populations (including the same time period)? Were inclusion and exclusion criteria for being in the study prespecified and applied uniformly to all participants?	X		
	5. Was a sample size justification, power description, or variance and effect estimates provided?		X	
	6. For the analyses in this paper, were the exposure(s) of interest measured prior to the outcome(s) being measured?	X		
	7. Was the timeframe sufficient so that one could reasonably expect to see an association between exposure and outcome if it existed?	X		

Source	Criteria	Yes	No	Cannot determine, not applicable
	8. For exposures that can vary in amount or level, did the study examine different levels of the exposure as related to the outcome (e.g., categories of exposure, or exposure measured as continuous variable)?	X		
	9. Were the exposure measures (independent variables) clearly defined, valid, reliable, and implemented consistently across all study participants?	X	X	
	10. Was the exposure(s) assessed more than once over time?			
	11. Were the outcome measures (dependent variables) clearly defined, valid, reliable, and implemented consistently across all study participants?	X		
	12. Were the outcome assessors blinded to the exposure status of participants?	X		
	13. Was loss to follow-up after baseline 20% or less?	X		
	14. Were key potential confounding variables measured and adjusted statistically for their impact on the relationship between exposure(s) and outcome(s)?	X		
	**Total**	12		
Chen et al (14), 2022 (China Nutrition and Health Survey)	1. Was the research question or objective in this paper clearly stated?	X		
	2. Was the study population clearly specified and defined?	X		
	3. Was the participation rate of eligible persons at least 50%?	X		
	4. Were all the subjects selected or recruited from the same or similar populations (including the same time period)? Were inclusion and exclusion criteria for being in the study prespecified and applied uniformly to all participants?	X		
	5. Was a sample size justification, power description, or variance and effect estimates provided?		X	
	6. For the analyses in this paper, were the exposure(s) of interest measured prior to the outcome(s) being measured?	X		
	7. Was the timeframe sufficient so that one could reasonably expect to see an association between exposure and outcome if it existed?	X		
	8. For exposures that can vary in amount or level, did the study examine different levels of the exposure as related to the outcome (e.g., categories of exposure, or exposure measured as continuous variable)?	X		
	9. Were the exposure measures (independent variables) clearly defined, valid, reliable, and implemented consistently across all study participants?	X		
	10. Was the exposure(s) assessed more than once over time?	X		
	11. Were the outcome measures (dependent variables) clearly defined, valid, reliable, and implemented consistently across all study participants?		X	
	12. Were the outcome assessors blinded to the exposure status of participants?	X		
	13. Was loss to follow-up after baseline 20% or less?		X	
	14. Were key potential confounding variables measured and adjusted statistically for their impact on the relationship between exposure(s) and outcome(s)?	X		
	**Total**	11		
Kim and Giovannucci (15), 2022	1. Was the research question or objective in this paper clearly stated?	X		
	2. Was the study population clearly specified and defined?	X		
	3. Was the participation rate of eligible persons at least 50%?	X		
	4. Were all the subjects selected or recruited from the same or similar populations (including the same time period)? Were inclusion and exclusion criteria for being in the study prespecified and applied uniformly to all participants?	X		
	5. Was a sample size justification, power description, or variance and effect estimates provided?		X	
	6. For the analyses in this paper, were the exposure(s) of interest measured prior to the outcome(s) being measured?	X		
	7. Was the timeframe sufficient so that one could reasonably expect to see an association between exposure and outcome if it existed?	X		
	8. For exposures that can vary in amount or level, did the study examine different levels of the exposure as related to the outcome (e.g., categories of exposure, or exposure measured as continuous variable)?	X		

Source	Criteria	Yes	No	Cannot determine, not applicable
	9. Were the exposure measures (independent variables) clearly defined, valid, reliable, and implemented consistently across all study participants?	X		
	10. Was the exposure(s) assessed more than once over time?	X		
	11. Were the outcome measures (dependent variables) clearly defined, valid, reliable, and implemented consistently across all study participants?	X		
	12. Were the outcome assessors blinded to the exposure status of participants?	X		
	13. Was loss to follow-up after baseline 20% or less?	X		
	14. Were key potential confounding variables measured and adjusted statistically for their impact on the relationship between exposure(s) and outcome(s)?	X		
	**Total**	13		
Glenn et al (16), 2023	1. Was the research question or objective in this paper clearly stated?	X		
	2. Was the study population clearly specified and defined?	X		
	3. Was the participation rate of eligible persons at least 50%?	X		
	4. Were all the subjects selected or recruited from the same or similar populations (including the same time period)? Were inclusion and exclusion criteria for being in the study prespecified and applied uniformly to all participants?	X		
	5. Was a sample size justification, power description, or variance and effect estimates provided?		X	
	6. For the analyses in this paper, were the exposure(s) of interest measured prior to the outcome(s) being measured?	X		
	7. Was the timeframe sufficient so that one could reasonably expect to see an association between exposure and outcome if it existed?	X		
	8. For exposures that can vary in amount or level, did the study examine different levels of the exposure as related to the outcome (e.g., categories of exposure, or exposure measured as continuous variable)?	X		
	9. Were the exposure measures (independent variables) clearly defined, valid, reliable, and implemented consistently across all study participants?	X		
	10. Was the exposure(s) assessed more than once over time?	X		
	11. Were the outcome measures (dependent variables) clearly defined, valid, reliable, and implemented consistently across all study participants?	X		
	12. Were the outcome assessors blinded to the exposure status of participants?	X		
	13. Was loss to follow-up after baseline 20% or less?	X		
	14. Were key potential confounding variables measured and adjusted statistically for their impact on the relationship between exposure(s) and outcome(s)?	X		
	**Total**	13		
Zhang et al (17), 2023 (a)	1. Was the research question or objective in this paper clearly stated?	X		
	2. Was the study population clearly specified and defined?	X		
	3. Was the participation rate of eligible persons at least 50%?	X		
	4. Were all the subjects selected or recruited from the same or similar populations (including the same time period)? Were inclusion and exclusion criteria for being in the study prespecified and applied uniformly to all participants?	X		
	5. Was a sample size justification, power description, or variance and effect estimates provided?		X	
	6. For the analyses in this paper, were the exposure(s) of interest measured prior to the outcome(s) being measured?	X		
	7. Was the timeframe sufficient so that one could reasonably expect to see an association between exposure and outcome if it existed?	X		
	8. For exposures that can vary in amount or level, did the study examine different levels of the exposure as related to the outcome (e.g., categories of exposure, or exposure measured as continuous variable)?	X		
	9. Were the exposure measures (independent variables) clearly defined, valid, reliable, and implemented consistently across all study participants?	X		
	10. Was the exposure(s) assessed more than once over time?		X	

Source	Criteria	Yes	No	Cannot determine, not applicable
	11. Were the outcome measures (dependent variables) clearly defined, valid, reliable, and implemented consistently across all study participants?	X		
	12. Were the outcome assessors blinded to the exposure status of participants?	X		
	13. Was loss to follow-up after baseline 20% or less?	X		
	14. Were key potential confounding variables measured and adjusted statistically for their impact on the relationship between exposure(s) and outcome(s)?	X		
	**Total**	12		
Satija et al (18), 2017 (NHS, NHSII, HPFS)	1. Was the research question or objective in this paper clearly stated?	X		
	2. Was the study population clearly specified and defined?	X		
	3. Was the participation rate of eligible persons at least 50%?	X		
	4. Were all the subjects selected or recruited from the same or similar populations (including the same time period)? Were inclusion and exclusion criteria for being in the study prespecified and applied uniformly to all participants?	X		
	5. Was a sample size justification, power description, or variance and effect estimates provided?		X	
	6. For the analyses in this paper, were the exposure(s) of interest measured prior to the outcome(s) being measured?	X		
	7. Was the timeframe sufficient so that one could reasonably expect to see an association between exposure and outcome if it existed?	X		
	8. For exposures that can vary in amount or level, did the study examine different levels of the exposure as related to the outcome (e.g., categories of exposure, or exposure measured as continuous variable)?	X		
	9. Were the exposure measures (independent variables) clearly defined, valid, reliable, and implemented consistently across all study participants?	X		
	10. Was the exposure(s) assessed more than once over time?	X		
	11. Were the outcome measures (dependent variables) clearly defined, valid, reliable, and implemented consistently across all study participants?	X		
	12. Were the outcome assessors blinded to the exposure status of participants?	X		
	13. Was loss to follow-up after baseline 20% or less?	X		
	14. Were key potential confounding variables measured and adjusted statistically for their impact on the relationship between exposure(s) and outcome(s)?	X		
	**Total**	13		
Kim et al (19), 2019	1. Was the research question or objective in this paper clearly stated?	X		
	2. Was the study population clearly specified and defined?	X		
	3. Was the participation rate of eligible persons at least 50%?	X		
	4. Were all the subjects selected or recruited from the same or similar populations (including the same time period)? Were inclusion and exclusion criteria for being in the study prespecified and applied uniformly to all participants?	X		
	5. Was a sample size justification, power description, or variance and effect estimates provided?		X	
	6. For the analyses in this paper, were the exposure(s) of interest measured prior to the outcome(s) being measured?	X		
	7. Was the timeframe sufficient so that one could reasonably expect to see an association between exposure and outcome if it existed?	X		
	8. For exposures that can vary in amount or level, did the study examine different levels of the exposure as related to the outcome (e.g., categories of exposure, or exposure measured as continuous variable)?	X		
	9. Were the exposure measures (independent variables) clearly defined, valid, reliable, and implemented consistently across all study participants?	X		
	10. Was the exposure(s) assessed more than once over time?	X		
	11. Were the outcome measures (dependent variables) clearly defined, valid, reliable, and implemented consistently across all study participants?	X		
	12. Were the outcome assessors blinded to the exposure status of participants?	X		

Source	Criteria	Yes	No	Cannot determine, not applicable
	13. Was loss to follow-up after baseline 20% or less?			X
	14. Were key potential confounding variables measured and adjusted statistically for their impact on the relationship between exposure(s) and outcome(s)?	X		
	**Total**	12		
Tong et al (20), 2019	1. Was the research question or objective in this paper clearly stated?	X		
	2. Was the study population clearly specified and defined?	X		
	3. Was the participation rate of eligible persons at least 50%?	X		
	4. Were all the subjects selected or recruited from the same or similar populations (including the same time period)? Were inclusion and exclusion criteria for being in the study prespecified and applied uniformly to all participants?	X		
	5. Was a sample size justification, power description, or variance and effect estimates provided?		X	
	6. For the analyses in this paper, were the exposure(s) of interest measured prior to the outcome(s) being measured?	X		
	7. Was the timeframe sufficient so that one could reasonably expect to see an association between exposure and outcome if it existed?	X		
	8. For exposures that can vary in amount or level, did the study examine different levels of the exposure as related to the outcome (e.g., categories of exposure, or exposure measured as continuous variable)?	X		
	9. Were the exposure measures (independent variables) clearly defined, valid, reliable, and implemented consistently across all study participants?	X		
	10. Was the exposure(s) assessed more than once over time?	X		
	11. Were the outcome measures (dependent variables) clearly defined, valid, reliable, and implemented consistently across all study participants?	X		
	12. Were the outcome assessors blinded to the exposure status of participants?	X		
	13. Was loss to follow-up after baseline 20% or less?	X		
	14. Were key potential confounding variables measured and adjusted statistically for their impact on the relationship between exposure(s) and outcome(s)?	X		
	**Total**	13		
Chiu et al (21), 2020	1. Was the research question or objective in this paper clearly stated?	X		
	2. Was the study population clearly specified and defined?	X		
	3. Was the participation rate of eligible persons at least 50%?	X		
	4. Were all the subjects selected or recruited from the same or similar populations (including the same time period)? Were inclusion and exclusion criteria for being in the study prespecified and applied uniformly to all participants?	X		
	5. Was a sample size justification, power description, or variance and effect estimates provided?		X	
	6. For the analyses in this paper, were the exposure(s) of interest measured prior to the outcome(s) being measured?	X		
	7. Was the timeframe sufficient so that one could reasonably expect to see an association between exposure and outcome if it existed?	X		
	8. For exposures that can vary in amount or level, did the study examine different levels of the exposure as related to the outcome (e.g., categories of exposure, or exposure measured as continuous variable)?			X
	9. Were the exposure measures (independent variables) clearly defined, valid, reliable, and implemented consistently across all study participants?	X		
	10. Was the exposure(s) assessed more than once over time?		X	
	11. Were the outcome measures (dependent variables) clearly defined, valid, reliable, and implemented consistently across all study participants?	X		
	12. Were the outcome assessors blinded to the exposure status of participants?	X		
	13. Was loss to follow-up after baseline 20% or less?			X
	14. Were key potential confounding variables measured and adjusted statistically for their impact on the relationship between exposure(s) and outcome(s)?	X		

Source	Criteria	Yes	No	Cannot determine, not applicable
	**Total**	10		
Shan et al (22), 2020 (NHS, NHSII, HPFS)	1. Was the research question or objective in this paper clearly stated?	X		
	2. Was the study population clearly specified and defined?	X		
	3. Was the participation rate of eligible persons at least 50%?	X		
	4. Were all the subjects selected or recruited from the same or similar populations (including the same time period)? Were inclusion and exclusion criteria for being in the study prespecified and applied uniformly to all participants?	X		
	5. Was a sample size justification, power description, or variance and effect estimates provided?	X		
	6. For the analyses in this paper, were the exposure(s) of interest measured prior to the outcome(s) being measured?	X		
	7. Was the timeframe sufficient so that one could reasonably expect to see an association between exposure and outcome if it existed?	X		
	8. For exposures that can vary in amount or level, did the study examine different levels of the exposure as related to the outcome (e.g., categories of exposure, or exposure measured as continuous variable)?	X		
	9. Were the exposure measures (independent variables) clearly defined, valid, reliable, and implemented consistently across all study participants?	X		
	10. Was the exposure(s) assessed more than once over time?	X		
	11. Were the outcome measures (dependent variables) clearly defined, valid, reliable, and implemented consistently across all study participants?	X		
	12. Were the outcome assessors blinded to the exposure status of participants?	X		
	13. Was loss to follow-up after baseline 20% or less?	X		
	14. Were key potential confounding variables measured and adjusted statistically for their impact on the relationship between exposure(s) and outcome(s)?	X		
	**Total**	14		
Baden et al (23), 2021 (NHS, NHSII, HPFS)	1. Was the research question or objective in this paper clearly stated?	X		
	2. Was the study population clearly specified and defined?	X		
	3. Was the participation rate of eligible persons at least 50%?	X		
	4. Were all the subjects selected or recruited from the same or similar populations (including the same time period)? Were inclusion and exclusion criteria for being in the study prespecified and applied uniformly to all participants?	X		
	5. Was a sample size justification, power description, or variance and effect estimates provided?		X	
	6. For the analyses in this paper, were the exposure(s) of interest measured prior to the outcome(s) being measured?	X		
	7. Was the timeframe sufficient so that one could reasonably expect to see an association between exposure and outcome if it existed?	X		
	8. For exposures that can vary in amount or level, did the study examine different levels of the exposure as related to the outcome (e.g., categories of exposure, or exposure measured as continuous variable)?	X		
	9. Were the exposure measures (independent variables) clearly defined, valid, reliable, and implemented consistently across all study participants?	X		
	10. Was the exposure(s) assessed more than once over time?	X		
	11. Were the outcome measures (dependent variables) clearly defined, valid, reliable, and implemented consistently across all study participants?	X		
	12. Were the outcome assessors blinded to the exposure status of participants?	X		
	13. Was loss to follow-up after baseline 20% or less?	X		
	14. Were key potential confounding variables measured and adjusted statistically for their impact on the relationship between exposure(s) and outcome(s)?	X		
	**Total**	13		

Source	Criteria	Yes	No	Cannot determine, not applicable
Glenn et al (24), 2021	1. Was the research question or objective in this paper clearly stated?	X		
	2. Was the study population clearly specified and defined?	X		
	3. Was the participation rate of eligible persons at least 50%?	X		
	4. Were all the subjects selected or recruited from the same or similar populations (including the same time period)? Were inclusion and exclusion criteria for being in the study prespecified and applied uniformly to all participants?	X		
	5. Was a sample size justification, power description, or variance and effect estimates provided?		X	
	6. For the analyses in this paper, were the exposure(s) of interest measured prior to the outcome(s) being measured?	X		
	7. Was the timeframe sufficient so that one could reasonably expect to see an association between exposure and outcome if it existed?	X		
	8. For exposures that can vary in amount or level, did the study examine different levels of the exposure as related to the outcome (e.g., categories of exposure, or exposure measured as continuous variable)?	X		
	9. Were the exposure measures (independent variables) clearly defined, valid, reliable, and implemented consistently across all study participants?	X		
	10. Was the exposure(s) assessed more than once over time?	X		
	11. Were the outcome measures (dependent variables) clearly defined, valid, reliable, and implemented consistently across all study participants?	X		
	12. Were the outcome assessors blinded to the exposure status of participants?	X		
	13. Was loss to follow-up after baseline 20% or less?			X
	14. Were key potential confounding variables measured and adjusted statistically for their impact on the relationship between exposure(s) and outcome(s)?	X		
	**Total**	12		
Petermann-Rocha et al (25), 2021	1. Was the research question or objective in this paper clearly stated?	X		
	2. Was the study population clearly specified and defined?	X		
	3. Was the participation rate of eligible persons at least 50%?	X		
	4. Were all the subjects selected or recruited from the same or similar populations (including the same time period)? Were inclusion and exclusion criteria for being in the study prespecified and applied uniformly to all participants?	X		
	5. Was a sample size justification, power description, or variance and effect estimates provided?		X	
	6. For the analyses in this paper, were the exposure(s) of interest measured prior to the outcome(s) being measured?	X		
	7. Was the timeframe sufficient so that one could reasonably expect to see an association between exposure and outcome if it existed?	X		
	8. For exposures that can vary in amount or level, did the study examine different levels of the exposure as related to the outcome (e.g., categories of exposure, or exposure measured as continuous variable)?			X
	9. Were the exposure measures (independent variables) clearly defined, valid, reliable, and implemented consistently across all study participants?	X		
	10. Was the exposure(s) assessed more than once over time?		X	
	11. Were the outcome measures (dependent variables) clearly defined, valid, reliable, and implemented consistently across all study participants?	X		
	12. Were the outcome assessors blinded to the exposure status of participants?	X		
	13. Was loss to follow-up after baseline 20% or less?			X
	14. Were key potential confounding variables measured and adjusted statistically for their impact on the relationship between exposure(s) and outcome(s)?	X		
	**Total**	10		
Chen et al (26), 2022	1. Was the research question or objective in this paper clearly stated?	X		
	2. Was the study population clearly specified and defined?	X		

Source	Criteria	Yes	No	Cannot determine, not applicable
(HCHS/SOL)	3. Was the participation rate of eligible persons at least 50%?	X		
	4. Were all the subjects selected or recruited from the same or similar populations (including the same time period)? Were inclusion and exclusion criteria			
	for being in the study prespecified and applied uniformly to all participants?	X		
	5. Was a sample size justification, power description, or variance and effect estimates provided?		X	
	6. For the analyses in this paper, were the exposure(s) of interest measured prior to the outcome(s) being measured?			
	7. Was the timeframe sufficient so that one could reasonably expect to see an association between exposure and outcome if it existed?	X		
	8. For exposures that can vary in amount or level, did the study examine different levels of the exposure as related to the outcome (e.g., categories of	X		
	exposure, or exposure measured as continuous variable)?	X		
	9. Were the exposure measures (independent variables) clearly defined, valid, reliable, and implemented consistently across all study participants?			
	10. Was the exposure(s) assessed more than once over time?	X		
	11. Were the outcome measures (dependent variables) clearly defined, valid, reliable, and implemented consistently across all study participants?	X		
	12. Were the outcome assessors blinded to the exposure status of participants?	X		
	13. Was loss to follow-up after baseline 20% or less?	X	X	
	14. Were key potential confounding variables measured and adjusted statistically for their impact on the relationship between exposure(s) and outcome(s)?			
	**Total**	X		
		12		
Choi et al (27), 2022	1. Was the research question or objective in this paper clearly stated?	X		
	2. Was the study population clearly specified and defined?	X		
	3. Was the participation rate of eligible persons at least 50%?	X		
	4. Were all the subjects selected or recruited from the same or similar populations (including the same time period)? Were inclusion and exclusion criteria for being in the study prespecified and applied uniformly to all participants?	X		
	5. Was a sample size justification, power description, or variance and effect estimates provided?		X	
	6. For the analyses in this paper, were the exposure(s) of interest measured prior to the outcome(s) being measured?	X		
	7. Was the timeframe sufficient so that one could reasonably expect to see an association between exposure and outcome if it existed?	X		
	8. For exposures that can vary in amount or level, did the study examine different levels of the exposure as related to the outcome (e.g., categories of exposure, or exposure measured as continuous variable)?	X		
	9. Were the exposure measures (independent variables) clearly defined, valid, reliable, and implemented consistently across all study participants?	X		
	10. Was the exposure(s) assessed more than once over time?	X		
	11. Were the outcome measures (dependent variables) clearly defined, valid, reliable, and implemented consistently across all study participants?	X		
	12. Were the outcome assessors blinded to the exposure status of participants?	X		
	13. Was loss to follow-up after baseline 20% or less?	X		
	14. Were key potential confounding variables measured and adjusted statistically for their impact on the relationship between exposure(s) and outcome(s)?	X		
	**Total**	13		
Ibsen et al (28), 2022	1. Was the research question or objective in this paper clearly stated?	X		
	2. Was the study population clearly specified and defined?	X		
	3. Was the participation rate of eligible persons at least 50%?		X	

Source	Criteria	Yes	No	Cannot determine, not applicable
	4. Were all the subjects selected or recruited from the same or similar populations (including the same time period)? Were inclusion and exclusion criteria for being in the study prespecified and applied uniformly to all participants?	X		
	5. Was a sample size justification, power description, or variance and effect estimates provided?		X	
	6. For the analyses in this paper, were the exposure(s) of interest measured prior to the outcome(s) being measured?	X		
	7. Was the timeframe sufficient so that one could reasonably expect to see an association between exposure and outcome if it existed?	X		
	8. For exposures that can vary in amount or level, did the study examine different levels of the exposure as related to the outcome (e.g., categories of exposure, or exposure measured as continuous variable)?	X		
	9. Were the exposure measures (independent variables) clearly defined, valid, reliable, and implemented consistently across all study participants?	X		
	10. Was the exposure(s) assessed more than once over time?		X	
	11. Were the outcome measures (dependent variables) clearly defined, valid, reliable, and implemented consistently across all study participants?	X		
	12. Were the outcome assessors blinded to the exposure status of participants?	X		
	13. Was loss to follow-up after baseline 20% or less?	X		
	14. Were key potential confounding variables measured and adjusted statistically for their impact on the relationship between exposure(s) and outcome(s)?	X		
	**Total**	11		
Kouvari et al (29), 2022	1. Was the research question or objective in this paper clearly stated?	X		
	2. Was the study population clearly specified and defined?	X		
	3. Was the participation rate of eligible persons at least 50%?	X		
	4. Were all the subjects selected or recruited from the same or similar populations (including the same time period)? Were inclusion and exclusion criteria for being in the study prespecified and applied uniformly to all participants?	X		
	5. Was a sample size justification, power description, or variance and effect estimates provided?	X		
	6. For the analyses in this paper, were the exposure(s) of interest measured prior to the outcome(s) being measured?	X		
	7. Was the timeframe sufficient so that one could reasonably expect to see an association between exposure and outcome if it existed?	X		
	8. For exposures that can vary in amount or level, did the study examine different levels of the exposure as related to the outcome (e.g., categories of exposure, or exposure measured as continuous variable)?	X		
	9. Were the exposure measures (independent variables) clearly defined, valid, reliable, and implemented consistently across all study participants?	X		
	10. Was the exposure(s) assessed more than once over time?		X	
	11. Were the outcome measures (dependent variables) clearly defined, valid, reliable, and implemented consistently across all study participants?	X		
	12. Were the outcome assessors blinded to the exposure status of participants?	X		
	13. Was loss to follow-up after baseline 20% or less?	X		
	14. Were key potential confounding variables measured and adjusted statistically for their impact on the relationship between exposure(s) and outcome(s)?	X		
	**Total**	13		
Lazarova et al (30), 2022	1. Was the research question or objective in this paper clearly stated?	X		
	2. Was the study population clearly specified and defined?		X	
	3. Was the participation rate of eligible persons at least 50%?		X	
	4. Were all the subjects selected or recruited from the same or similar populations (including the same time period)? Were inclusion and exclusion criteria for being in the study prespecified and applied uniformly to all participants?		X	

Source	Criteria	Yes	No	Cannot determine, not applicable
	5. Was a sample size justification, power description, or variance and effect estimates provided?		X	
	6. For the analyses in this paper, were the exposure(s) of interest measured prior to the outcome(s) being measured?	X		
	7. Was the timeframe sufficient so that one could reasonably expect to see an association between exposure and outcome if it existed?	X		
	8. For exposures that can vary in amount or level, did the study examine different levels of the exposure as related to the outcome (e.g., categories of exposure, or exposure measured as continuous variable)?	X		
	9. Were the exposure measures (independent variables) clearly defined, valid, reliable, and implemented consistently across all study participants?	X		
	10. Was the exposure(s) assessed more than once over time?	X		
	11. Were the outcome measures (dependent variables) clearly defined, valid, reliable, and implemented consistently across all study participants?	X		
	12. Were the outcome assessors blinded to the exposure status of participants?	X		
	13. Was loss to follow-up after baseline 20% or less?	X		
	14. Were key potential confounding variables measured and adjusted statistically for their impact on the relationship between exposure(s) and outcome(s)?	X		
	**Total**	10		
Zhang et al (31), 2023 (b)	1. Was the research question or objective in this paper clearly stated?	X		
	2. Was the study population clearly specified and defined?	X		
	3. Was the participation rate of eligible persons at least 50%?	X		
	4. Were all the subjects selected or recruited from the same or similar populations (including the same time period)? Were inclusion and exclusion criteria for being in the study prespecified and applied uniformly to all participants?	X		
	5. Was a sample size justification, power description, or variance and effect estimates provided?		X	
	6. For the analyses in this paper, were the exposure(s) of interest measured prior to the outcome(s) being measured?	X		
	7. Was the timeframe sufficient so that one could reasonably expect to see an association between exposure and outcome if it existed?	X		
	8. For exposures that can vary in amount or level, did the study examine different levels of the exposure as related to the outcome (e.g., categories of exposure, or exposure measured as continuous variable)?	X		
	9. Were the exposure measures (independent variables) clearly defined, valid, reliable, and implemented consistently across all study participants?	X		
	10. Was the exposure(s) assessed more than once over time?		X	
	11. Were the outcome measures (dependent variables) clearly defined, valid, reliable, and implemented consistently across all study participants?	X		
	12. Were the outcome assessors blinded to the exposure status of participants?	X		
	13. Was loss to follow-up after baseline 20% or less?	X		
	14. Were key potential confounding variables measured and adjusted statistically for their impact on the relationship between exposure(s) and outcome(s)?	X		
	**Total**	12		
Thompson et al (32), 2023	1. Was the research question or objective in this paper clearly stated?	X		
	2. Was the study population clearly specified and defined?	X		
	3. Was the participation rate of eligible persons at least 50%?	X		
	4. Were all the subjects selected or recruited from the same or similar populations (including the same time period)? Were inclusion and exclusion criteria for being in the study prespecified and applied uniformly to all participants?	X		
	5. Was a sample size justification, power description, or variance and effect estimates provided?		X	
	6. For the analyses in this paper, were the exposure(s) of interest measured prior to the outcome(s) being measured?	X		

Source	Criteria	Yes	No	Cannot determine, not applicable
	7. Was the timeframe sufficient so that one could reasonably expect to see an association between exposure and outcome if it existed?	X		
	8. For exposures that can vary in amount or level, did the study examine different levels of the exposure as related to the outcome (e.g., categories of exposure, or exposure measured as continuous variable)?	X		
	9. Were the exposure measures (independent variables) clearly defined, valid, reliable, and implemented consistently across all study participants?			
	10. Was the exposure(s) assessed more than once over time?	X	X	
	11. Were the outcome measures (dependent variables) clearly defined, valid, reliable, and implemented consistently across all study participants?			
	12. Were the outcome assessors blinded to the exposure status of participants?	X		
	13. Was loss to follow-up after baseline 20% or less?	X	X	
	14. Were key potential confounding variables measured and adjusted statistically for their impact on the relationship between exposure(s) and outcome(s)?			
	**Total**	X<br>11		
Weston et al (33), 2022	1. Was the research question or objective in this paper clearly stated?	X		
	2. Was the study population clearly specified and defined?	X		
	3. Was the participation rate of eligible persons at least 50%?	X		
	4. Were all the subjects selected or recruited from the same or similar populations (including the same time period)? Were inclusion and exclusion criteria for being in the study prespecified and applied uniformly to all participants?	X		
	5. Was a sample size justification, power description, or variance and effect estimates provided?		X	
	6. For the analyses in this paper, were the exposure(s) of interest measured prior to the outcome(s) being measured?	X		
	7. Was the timeframe sufficient so that one could reasonably expect to see an association between exposure and outcome if it existed?	X		
	8. For exposures that can vary in amount or level, did the study examine different levels of the exposure as related to the outcome (e.g., categories of exposure, or exposure measured as continuous variable)?	X		
	9. Were the exposure measures (independent variables) clearly defined, valid, reliable, and implemented consistently across all study participants?	X		
	10. Was the exposure(s) assessed more than once over time?		X	
	11. Were the outcome measures (dependent variables) clearly defined, valid, reliable, and implemented consistently across all study participants?	X		
	12. Were the outcome assessors blinded to the exposure status of participants?	X		
	13. Was loss to follow-up after baseline 20% or less?	X		
	14. Were key potential confounding variables measured and adjusted statistically for their impact on the relationship between exposure(s) and outcome(s)?	X		
	**Total**	12		
Berkel and de Waard (34), 1983	1. Was the research question or objective in this paper clearly stated?	X		
	2. Was the study population clearly specified and defined?	X		
	3. Was the participation rate of eligible persons at least 50%?	X		
	4. Were all the subjects selected or recruited from the same or similar populations (including the same time period)? Were inclusion and exclusion criteria for being in the study prespecified and applied uniformly to all participants?		X	
	5. Was a sample size justification, power description, or variance and effect estimates provided?		X	
	6. For the analyses in this paper, were the exposure(s) of interest measured prior to the outcome(s) being measured?	X		
	7. Was the timeframe sufficient so that one could reasonably expect to see an association between exposure and outcome if it existed?	X		

Source	Criteria	Yes	No	Cannot determine, not applicable
	8. For exposures that can vary in amount or level, did the study examine different levels of the exposure as related to the outcome (e.g., categories of exposure, or exposure measured as continuous variable)?			X
	9. Were the exposure measures (independent variables) clearly defined, valid, reliable, and implemented consistently across all study participants?		X	
	10. Was the exposure(s) assessed more than once over time?		X	
	11. Were the outcome measures (dependent variables) clearly defined, valid, reliable, and implemented consistently across all study participants?		X	
	12. Were the outcome assessors blinded to the exposure status of participants?	X		
	13. Was loss to follow-up after baseline 20% or less?			X
	14. Were key potential confounding variables measured and adjusted statistically for their impact on the relationship between exposure(s) and outcome(s)?			X
	**Total**	6		
Ogata et al (35), 1984	1. Was the research question or objective in this paper clearly stated?	X		
	2. Was the study population clearly specified and defined?	X		
	3. Was the participation rate of eligible persons at least 50%?	X		
	4. Were all the subjects selected or recruited from the same or similar populations (including the same time period)? Were inclusion and exclusion criteria for being in the study prespecified and applied uniformly to all participants?		X	
	5. Was a sample size justification, power description, or variance and effect estimates provided?		X	
	6. For the analyses in this paper, were the exposure(s) of interest measured prior to the outcome(s) being measured?	X		
	7. Was the timeframe sufficient so that one could reasonably expect to see an association between exposure and outcome if it existed?	X		
	8. For exposures that can vary in amount or level, did the study examine different levels of the exposure as related to the outcome (e.g., categories of exposure, or exposure measured as continuous variable)?			X
	9. Were the exposure measures (independent variables) clearly defined, valid, reliable, and implemented consistently across all study participants?		X	
	10. Was the exposure(s) assessed more than once over time?		X	
	11. Were the outcome measures (dependent variables) clearly defined, valid, reliable, and implemented consistently across all study participants?		X	
	12. Were the outcome assessors blinded to the exposure status of participants?	X		
	13. Was loss to follow-up after baseline 20% or less?			X
	14. Were key potential confounding variables measured and adjusted statistically for their impact on the relationship between exposure(s) and outcome(s)?			X
	**Total**	6		
Thorogood et al (36), 1994	1. Was the research question or objective in this paper clearly stated?	X		
	2. Was the study population clearly specified and defined?	X		
	3. Was the participation rate of eligible persons at least 50%?			X
	4. Were all the subjects selected or recruited from the same or similar populations (including the same time period)? Were inclusion and exclusion criteria for being in the study prespecified and applied uniformly to all participants?		X	
	5. Was a sample size justification, power description, or variance and effect estimates provided?		X	
	6. For the analyses in this paper, were the exposure(s) of interest measured prior to the outcome(s) being measured?	X		
	7. Was the timeframe sufficient so that one could reasonably expect to see an association between exposure and outcome if it existed?	X		
	8. For exposures that can vary in amount or level, did the study examine different levels of the exposure as related to the outcome (e.g., categories of exposure, or exposure measured as continuous variable)?			X

Source	Criteria	Yes	No	Cannot determine, not applicable
	9. Were the exposure measures (independent variables) clearly defined, valid, reliable, and implemented consistently across all study participants?	X		
	10. Was the exposure(s) assessed more than once over time?		X	
	11. Were the outcome measures (dependent variables) clearly defined, valid, reliable, and implemented consistently across all study participants?	X		
	12. Were the outcome assessors blinded to the exposure status of participants?	X		
	13. Was loss to follow-up after baseline 20% or less?			X
	14. Were key potential confounding variables measured and adjusted statistically for their impact on the relationship between exposure(s) and outcome(s)?		X	
	**Total**	7		
Key et al (37), 1996	1. Was the research question or objective in this paper clearly stated?	X		
	2. Was the study population clearly specified and defined?	X		
	3. Was the participation rate of eligible persons at least 50%?			X
	4. Were all the subjects selected or recruited from the same or similar populations (including the same time period)? Were inclusion and exclusion criteria for being in the study prespecified and applied uniformly to all participants?		X	
	5. Was a sample size justification, power description, or variance and effect estimates provided?		X	
	6. For the analyses in this paper, were the exposure(s) of interest measured prior to the outcome(s) being measured?	X		
	7. Was the timeframe sufficient so that one could reasonably expect to see an association between exposure and outcome if it existed?	X		
	8. For exposures that can vary in amount or level, did the study examine different levels of the exposure as related to the outcome (e.g., categories of exposure, or exposure measured as continuous variable)?			X
	9. Were the exposure measures (independent variables) clearly defined, valid, reliable, and implemented consistently across all study participants?	X		
	10. Was the exposure(s) assessed more than once over time?		X	
	11. Were the outcome measures (dependent variables) clearly defined, valid, reliable, and implemented consistently across all study participants?	X		
	12. Were the outcome assessors blinded to the exposure status of participants?	X		
	13. Was loss to follow-up after baseline 20% or less?	X		
	14. Were key potential confounding variables measured and adjusted statistically for their impact on the relationship between exposure(s) and outcome(s)?		X	
	**Total**	8		
Key et al (38), 1999	1. Was the research question or objective in this paper clearly stated?	X		
	2. Was the study population clearly specified and defined?	X		
	3. Was the participation rate of eligible persons at least 50%?			X
	4. Were all the subjects selected or recruited from the same or similar populations (including the same time period)? Were inclusion and exclusion criteria for being in the study prespecified and applied uniformly to all participants?	X		
	5. Was a sample size justification, power description, or variance and effect estimates provided?		X	
	6. For the analyses in this paper, were the exposure(s) of interest measured prior to the outcome(s) being measured?	X		
	7. Was the timeframe sufficient so that one could reasonably expect to see an association between exposure and outcome if it existed?	X		
	8. For exposures that can vary in amount or level, did the study examine different levels of the exposure as related to the outcome (e.g., categories of exposure, or exposure measured as continuous variable)?			X
	9. Were the exposure measures (independent variables) clearly defined, valid, reliable, and implemented consistently across all study participants?	X		
	10. Was the exposure(s) assessed more than once over time?		X	

Source	Criteria	Yes	No	Cannot determine, not applicable
	11. Were the outcome measures (dependent variables) clearly defined, valid, reliable, and implemented consistently across all study participants?	X		
	12. Were the outcome assessors blinded to the exposure status of participants?	X		
	13. Was loss to follow-up after baseline 20% or less?			X
	14. Were key potential confounding variables measured and adjusted statistically for their impact on the relationship between exposure(s) and outcome(s)?		X	
	**Total**	8		
Appleby et al (39), 2001	1. Was the research question or objective in this paper clearly stated?	X		
	2. Was the study population clearly specified and defined?	X		
	3. Was the participation rate of eligible persons at least 50%?			X
	4. Were all the subjects selected or recruited from the same or similar populations (including the same time period)? Were inclusion and exclusion criteria for being in the study prespecified and applied uniformly to all participants?	X		
	5. Was a sample size justification, power description, or variance and effect estimates provided?		X	
	6. For the analyses in this paper, were the exposure(s) of interest measured prior to the outcome(s) being measured?	X		
	7. Was the timeframe sufficient so that one could reasonably expect to see an association between exposure and outcome if it existed?	X		
	8. For exposures that can vary in amount or level, did the study examine different levels of the exposure as related to the outcome (e.g., categories of exposure, or exposure measured as continuous variable)?			X
	9. Were the exposure measures (independent variables) clearly defined, valid, reliable, and implemented consistently across all study participants?	X		
	10. Was the exposure(s) assessed more than once over time?		X	
	11. Were the outcome measures (dependent variables) clearly defined, valid, reliable, and implemented consistently across all study participants?	X		
	12. Were the outcome assessors blinded to the exposure status of participants?	X		
	13. Was loss to follow-up after baseline 20% or less?			X
	14. Were key potential confounding variables measured and adjusted statistically for their impact on the relationship between exposure(s) and outcome(s)?		X	
	**Total**	8		
Chang-Claude et al (40), 2005	1. Was the research question or objective in this paper clearly stated?	X		
	2. Was the study population clearly specified and defined?	X		
	3. Was the participation rate of eligible persons at least 50%?	X		
	4. Were all the subjects selected or recruited from the same or similar populations (including the same time period)? Were inclusion and exclusion criteria for being in the study prespecified and applied uniformly to all participants?	X		
	5. Was a sample size justification, power description, or variance and effect estimates provided?		X	
	6. For the analyses in this paper, were the exposure(s) of interest measured prior to the outcome(s) being measured?	X		
	7. Was the timeframe sufficient so that one could reasonably expect to see an association between exposure and outcome if it existed?	X		
	8. For exposures that can vary in amount or level, did the study examine different levels of the exposure as related to the outcome (e.g., categories of exposure, or exposure measured as continuous variable)?			X
	9. Were the exposure measures (independent variables) clearly defined, valid, reliable, and implemented consistently across all study participants?	X		
	10. Was the exposure(s) assessed more than once over time?		X	
	11. Were the outcome measures (dependent variables) clearly defined, valid, reliable, and implemented consistently across all study participants?	X		
	12. Were the outcome assessors blinded to the exposure status of participants?	X		

Source	Criteria	Yes	No	Cannot determine, not applicable
	13. Was loss to follow-up after baseline 20% or less?	X		
	14. Were key potential confounding variables measured and adjusted statistically for their impact on the relationship between exposure(s) and outcome(s)?	X		
	**Total**	11		
Bamia et al (41), 2007	1. Was the research question or objective in this paper clearly stated?	X		
	2. Was the study population clearly specified and defined?	X		
	3. Was the participation rate of eligible persons at least 50%?	X		
	4. Were all the subjects selected or recruited from the same or similar populations (including the same time period)? Were inclusion and exclusion criteria for being in the study prespecified and applied uniformly to all participants?	X		
	5. Was a sample size justification, power description, or variance and effect estimates provided?		X	
	6. For the analyses in this paper, were the exposure(s) of interest measured prior to the outcome(s) being measured?	X		
	7. Was the timeframe sufficient so that one could reasonably expect to see an association between exposure and outcome if it existed?	X		
	8. For exposures that can vary in amount or level, did the study examine different levels of the exposure as related to the outcome (e.g., categories of exposure, or exposure measured as continuous variable)?	X		
	9. Were the exposure measures (independent variables) clearly defined, valid, reliable, and implemented consistently across all study participants?	X		
	10. Was the exposure(s) assessed more than once over time?		X	
	11. Were the outcome measures (dependent variables) clearly defined, valid, reliable, and implemented consistently across all study participants?	X		
	12. Were the outcome assessors blinded to the exposure status of participants?	X		
	13. Was loss to follow-up after baseline 20% or less?			X
	14. Were key potential confounding variables measured and adjusted statistically for their impact on the relationship between exposure(s) and outcome(s)?	X		
	**Total**	11		
Key et al (42), 2009	1. Was the research question or objective in this paper clearly stated?	X		
	2. Was the study population clearly specified and defined?	X		
	3. Was the participation rate of eligible persons at least 50%?	X		
	4. Were all the subjects selected or recruited from the same or similar populations (including the same time period)? Were inclusion and exclusion criteria for being in the study prespecified and applied uniformly to all participants?	X		
	5. Was a sample size justification, power description, or variance and effect estimates provided?		X	
	6. For the analyses in this paper, were the exposure(s) of interest measured prior to the outcome(s) being measured?	X		
	7. Was the timeframe sufficient so that one could reasonably expect to see an association between exposure and outcome if it existed?	X		
	8. For exposures that can vary in amount or level, did the study examine different levels of the exposure as related to the outcome (e.g., categories of exposure, or exposure measured as continuous variable)?	X		
	9. Were the exposure measures (independent variables) clearly defined, valid, reliable, and implemented consistently across all study participants?	X		
	10. Was the exposure(s) assessed more than once over time?		X	
	11. Were the outcome measures (dependent variables) clearly defined, valid, reliable, and implemented consistently across all study participants?	X		
	12. Were the outcome assessors blinded to the exposure status of participants?	X		
	13. Was loss to follow-up after baseline 20% or less?			X
	14. Were key potential confounding variables measured and adjusted statistically for their impact on the relationship between exposure(s) and outcome(s)?	X		

Source	Criteria	Yes	No	Cannot determine, not applicable
	**Total**	11		
Orlich et al (43), 2013	1. Was the research question or objective in this paper clearly stated?	X		
	2. Was the study population clearly specified and defined?	X		
	3. Was the participation rate of eligible persons at least 50%?	X		
	4. Were all the subjects selected or recruited from the same or similar populations (including the same time period)? Were inclusion and exclusion criteria for being in the study prespecified and applied uniformly to all participants?	X		
	5. Was a sample size justification, power description, or variance and effect estimates provided?		X	
	6. For the analyses in this paper, were the exposure(s) of interest measured prior to the outcome(s) being measured?	X		
	7. Was the timeframe sufficient so that one could reasonably expect to see an association between exposure and outcome if it existed?	X		
	8. For exposures that can vary in amount or level, did the study examine different levels of the exposure as related to the outcome (e.g., categories of exposure, or exposure measured as continuous variable)?	X		
	9. Were the exposure measures (independent variables) clearly defined, valid, reliable, and implemented consistently across all study participants?	X		
	10. Was the exposure(s) assessed more than once over time?		X	
	11. Were the outcome measures (dependent variables) clearly defined, valid, reliable, and implemented consistently across all study participants?	X		
	12. Were the outcome assessors blinded to the exposure status of participants?	X		
	13. Was loss to follow-up after baseline 20% or less?			X
	14. Were key potential confounding variables measured and adjusted statistically for their impact on the relationship between exposure(s) and outcome(s)?	X		
	**Total**	11		
Martínez-González et al (44), 2014	1. Was the research question or objective in this paper clearly stated?	X		
	2. Was the study population clearly specified and defined?	X		
	3. Was the participation rate of eligible persons at least 50%?	X		
	4. Were all the subjects selected or recruited from the same or similar populations (including the same time period)? Were inclusion and exclusion criteria			
	for being in the study prespecified and applied uniformly to all participants?	X		
	5. Was a sample size justification, power description, or variance and effect estimates provided?		X	
	6. For the analyses in this paper, were the exposure(s) of interest measured prior to the outcome(s) being measured?			
	7. Was the timeframe sufficient so that one could reasonably expect to see an association between exposure and outcome if it existed?	X		
	8. For exposures that can vary in amount or level, did the study examine different levels of the exposure as related to the outcome (e.g., categories of	X		
	exposure, or exposure measured as continuous variable)?	X		
	9. Were the exposure measures (independent variables) clearly defined, valid, reliable, and implemented consistently across all study participants?			
	10. Was the exposure(s) assessed more than once over time?	X		
	11. Were the outcome measures (dependent variables) clearly defined, valid, reliable, and implemented consistently across all study participants?	X		
	12. Were the outcome assessors blinded to the exposure status of participants?	X		
	13. Was loss to follow-up after baseline 20% or less?	X		X
	14. Were key potential confounding variables measured and adjusted statistically for their impact on the relationship between exposure(s) and outcome(s)?			
	**Total**	X		
		12		

Source	Criteria	Yes	No	Cannot determine, not applicable
Mihrshahi et al (45), 2016	1. Was the research question or objective in this paper clearly stated?	X		
	2. Was the study population clearly specified and defined?	X		
	3. Was the participation rate of eligible persons at least 50%?	X		
	4. Were all the subjects selected or recruited from the same or similar populations (including the same time period)? Were inclusion and exclusion criteria for being in the study prespecified and applied uniformly to all participants?	X		
	5. Was a sample size justification, power description, or variance and effect estimates provided?		X	
	6. For the analyses in this paper, were the exposure(s) of interest measured prior to the outcome(s) being measured?	X		
	7. Was the timeframe sufficient so that one could reasonably expect to see an association between exposure and outcome if it existed?	X		
	8. For exposures that can vary in amount or level, did the study examine different levels of the exposure as related to the outcome (e.g., categories of exposure, or exposure measured as continuous variable)?	X		
	9. Were the exposure measures (independent variables) clearly defined, valid, reliable, and implemented consistently across all study participants?	X		
	10. Was the exposure(s) assessed more than once over time?		X	
	11. Were the outcome measures (dependent variables) clearly defined, valid, reliable, and implemented consistently across all study participants?	X		
	12. Were the outcome assessors blinded to the exposure status of participants?	X		
	13. Was loss to follow-up after baseline 20% or less?			X
	14. Were key potential confounding variables measured and adjusted statistically for their impact on the relationship between exposure(s) and outcome(s)?	X		
	**Total**	11		
Kim et al (46), 2018	1. Was the research question or objective in this paper clearly stated?	X		
	2. Was the study population clearly specified and defined?	X		
	3. Was the participation rate of eligible persons at least 50%?	X		
	4. Were all the subjects selected or recruited from the same or similar populations (including the same time period)? Were inclusion and exclusion criteria for being in the study prespecified and applied uniformly to all participants?	X		
	5. Was a sample size justification, power description, or variance and effect estimates provided?		X	
	6. For the analyses in this paper, were the exposure(s) of interest measured prior to the outcome(s) being measured?	X		
	7. Was the timeframe sufficient so that one could reasonably expect to see an association between exposure and outcome if it existed?	X		
	8. For exposures that can vary in amount or level, did the study examine different levels of the exposure as related to the outcome (e.g., categories of exposure, or exposure measured as continuous variable)?	X		
	9. Were the exposure measures (independent variables) clearly defined, valid, reliable, and implemented consistently across all study participants?	X		
	10. Was the exposure(s) assessed more than once over time?		X	
	11. Were the outcome measures (dependent variables) clearly defined, valid, reliable, and implemented consistently across all study participants?	X		
	12. Were the outcome assessors blinded to the exposure status of participants?	X		
	13. Was loss to follow-up after baseline 20% or less?	X		
	14. Were key potential confounding variables measured and adjusted statistically for their impact on the relationship between exposure(s) and outcome(s)?	X		
	**Total**	12		
Baden et al (47), 2019	1. Was the research question or objective in this paper clearly stated?	X		
	2. Was the study population clearly specified and defined?	X		

Source	Criteria	Yes	No	Cannot determine, not applicable
	3. Was the participation rate of eligible persons at least 50%?	X		
	4. Were all the subjects selected or recruited from the same or similar populations (including the same time period)? Were inclusion and exclusion criteria for being in the study prespecified and applied uniformly to all participants?	X		
	5. Was a sample size justification, power description, or variance and effect estimates provided?		X	
	6. For the analyses in this paper, were the exposure(s) of interest measured prior to the outcome(s) being measured?	X		
	7. Was the timeframe sufficient so that one could reasonably expect to see an association between exposure and outcome if it existed?	X		
	8. For exposures that can vary in amount or level, did the study examine different levels of the exposure as related to the outcome (e.g., categories of exposure, or exposure measured as continuous variable)?	X		
	9. Were the exposure measures (independent variables) clearly defined, valid, reliable, and implemented consistently across all study participants?	X		
	10. Was the exposure(s) assessed more than once over time?	X		
	11. Were the outcome measures (dependent variables) clearly defined, valid, reliable, and implemented consistently across all study participants?	X		
	12. Were the outcome assessors blinded to the exposure status of participants?	X		
	13. Was loss to follow-up after baseline 20% or less?	X		
	14. Were key potential confounding variables measured and adjusted statistically for their impact on the relationship between exposure(s) and outcome(s)?	X		
	**Total**	13		
Anyene et al (48), 2021	1. Was the research question or objective in this paper clearly stated?	X		
	2. Was the study population clearly specified and defined?	X		
	3. Was the participation rate of eligible persons at least 50%?	X		
	4. Were all the subjects selected or recruited from the same or similar populations (including the same time period)? Were inclusion and exclusion criteria			
	for being in the study prespecified and applied uniformly to all participants?	X		
	5. Was a sample size justification, power description, or variance and effect estimates provided?		X	
	6. For the analyses in this paper, were the exposure(s) of interest measured prior to the outcome(s) being measured?			
	7. Was the timeframe sufficient so that one could reasonably expect to see an association between exposure and outcome if it existed?	X		
	8. For exposures that can vary in amount or level, did the study examine different levels of the exposure as related to the outcome (e.g., categories of	X		
	exposure, or exposure measured as continuous variable)?	X		
	9. Were the exposure measures (independent variables) clearly defined, valid, reliable, and implemented consistently across all study participants?			
	10. Was the exposure(s) assessed more than once over time?	X		
	11. Were the outcome measures (dependent variables) clearly defined, valid, reliable, and implemented consistently across all study participants?	X		
	12. Were the outcome assessors blinded to the exposure status of participants?	X		
	13. Was loss to follow-up after baseline 20% or less?	X		X
	14. Were key potential confounding variables measured and adjusted statistically for their impact on the relationship between exposure(s) and outcome(s)?			
	**Total**	X		
		12		
Kim et al (49), 2021	1. Was the research question or objective in this paper clearly stated?	X		
	2. Was the study population clearly specified and defined?	X		
	3. Was the participation rate of eligible persons at least 50%?	X		

Source	Criteria	Yes	No	Cannot determine, not applicable
	4. Were all the subjects selected or recruited from the same or similar populations (including the same time period)? Were inclusion and exclusion criteria for being in the study prespecified and applied uniformly to all participants?	X		
	5. Was a sample size justification, power description, or variance and effect estimates provided?		X	
	6. For the analyses in this paper, were the exposure(s) of interest measured prior to the outcome(s) being measured?	X		
	7. Was the timeframe sufficient so that one could reasonably expect to see an association between exposure and outcome if it existed?	X		
	8. For exposures that can vary in amount or level, did the study examine different levels of the exposure as related to the outcome (e.g., categories of exposure, or exposure measured as continuous variable)?	X		
	9. Were the exposure measures (independent variables) clearly defined, valid, reliable, and implemented consistently across all study participants?	X		
	10. Was the exposure(s) assessed more than once over time?	X		
	11. Were the outcome measures (dependent variables) clearly defined, valid, reliable, and implemented consistently across all study participants?	X		
	12. Were the outcome assessors blinded to the exposure status of participants?	X		
	13. Was loss to follow-up after baseline 20% or less?	X		
	14. Were key potential confounding variables measured and adjusted statistically for their impact on the relationship between exposure(s) and outcome(s)?	X		
	**Total**	13		
Lo et al (50), 2021	1. Was the research question or objective in this paper clearly stated?	X		
	2. Was the study population clearly specified and defined?	X		
	3. Was the participation rate of eligible persons at least 50%?			X
	4. Were all the subjects selected or recruited from the same or similar populations (including the same time period)? Were inclusion and exclusion criteria for being in the study prespecified and applied uniformly to all participants?	X		
	5. Was a sample size justification, power description, or variance and effect estimates provided?		X	
	6. For the analyses in this paper, were the exposure(s) of interest measured prior to the outcome(s) being measured?	X		
	7. Was the timeframe sufficient so that one could reasonably expect to see an association between exposure and outcome if it existed?	X		
	8. For exposures that can vary in amount or level, did the study examine different levels of the exposure as related to the outcome (e.g., categories of exposure, or exposure measured as continuous variable)?	X		
	9. Were the exposure measures (independent variables) clearly defined, valid, reliable, and implemented consistently across all study participants?	X		
	10. Was the exposure(s) assessed more than once over time?		X	
	11. Were the outcome measures (dependent variables) clearly defined, valid, reliable, and implemented consistently across all study participants?	X		
	12. Were the outcome assessors blinded to the exposure status of participants?	X		
	13. Was loss to follow-up after baseline 20% or less?			X
	14. Were key potential confounding variables measured and adjusted statistically for their impact on the relationship between exposure(s) and outcome(s)?	X		
	**Total**	10		
Ratjen et al (51), 2021	1. Was the research question or objective in this paper clearly stated?	X		
	2. Was the study population clearly specified and defined?	X		
	3. Was the participation rate of eligible persons at least 50%?	X		
	4. Were all the subjects selected or recruited from the same or similar populations (including the same time period)? Were inclusion and exclusion criteria for being in the study prespecified and applied uniformly to all participants?	X		

Source	Criteria	Yes	No	Cannot determine, not applicable
	5. Was a sample size justification, power description, or variance and effect estimates provided?		X	
	6. For the analyses in this paper, were the exposure(s) of interest measured prior to the outcome(s) being measured?	X		
	7. Was the timeframe sufficient so that one could reasonably expect to see an association between exposure and outcome if it existed?	X		
	8. For exposures that can vary in amount or level, did the study examine different levels of the exposure as related to the outcome (e.g., categories of exposure, or exposure measured as continuous variable)?	X		
	9. Were the exposure measures (independent variables) clearly defined, valid, reliable, and implemented consistently across all study participants?	X		
	10. Was the exposure(s) assessed more than once over time?	X		
	11. Were the outcome measures (dependent variables) clearly defined, valid, reliable, and implemented consistently across all study participants?	X		
	12. Were the outcome assessors blinded to the exposure status of participants?	X		
	13. Was loss to follow-up after baseline 20% or less?	X		
	14. Were key potential confounding variables measured and adjusted statistically for their impact on the relationship between exposure(s) and outcome(s)?	X		
	**Total**	13		
Chen et al (52), 2022 (CLHLS)	1. Was the research question or objective in this paper clearly stated?	X		
	2. Was the study population clearly specified and defined?	X		
	3. Was the participation rate of eligible persons at least 50%?	X		
	4. Were all the subjects selected or recruited from the same or similar populations (including the same time period)? Were inclusion and exclusion criteria for being in the study prespecified and applied uniformly to all participants?	X		
	5. Was a sample size justification, power description, or variance and effect estimates provided?	X		
	6. For the analyses in this paper, were the exposure(s) of interest measured prior to the outcome(s) being measured?	X		
	7. Was the timeframe sufficient so that one could reasonably expect to see an association between exposure and outcome if it existed?	X		
	8. For exposures that can vary in amount or level, did the study examine different levels of the exposure as related to the outcome (e.g., categories of exposure, or exposure measured as continuous variable)?	X		
	9. Were the exposure measures (independent variables) clearly defined, valid, reliable, and implemented consistently across all study participants?	X		
	10. Was the exposure(s) assessed more than once over time?		X	
	11. Were the outcome measures (dependent variables) clearly defined, valid, reliable, and implemented consistently across all study participants?	X		
	12. Were the outcome assessors blinded to the exposure status of participants?	X		
	13. Was loss to follow-up after baseline 20% or less?	X		
	14. Were key potential confounding variables measured and adjusted statistically for their impact on the relationship between exposure(s) and outcome(s)?		X	
	**Total**	12		
Delgado-Velandia et al (53), 2022	1. Was the research question or objective in this paper clearly stated?	X		
	2. Was the study population clearly specified and defined?	X		
	3. Was the participation rate of eligible persons at least 50%?	X		
	4. Were all the subjects selected or recruited from the same or similar populations (including the same time period)? Were inclusion and exclusion criteria for being in the study prespecified and applied uniformly to all participants?	X		
	5. Was a sample size justification, power description, or variance and effect estimates provided?		X	
	6. For the analyses in this paper, were the exposure(s) of interest measured prior to the outcome(s) being measured?	X		

Source	Criteria	Yes	No	Cannot determine, not applicable
	7. Was the timeframe sufficient so that one could reasonably expect to see an association between exposure and outcome if it existed?	X		
	8. For exposures that can vary in amount or level, did the study examine different levels of the exposure as related to the outcome (e.g., categories of exposure, or exposure measured as continuous variable)?	X		
	9. Were the exposure measures (independent variables) clearly defined, valid, reliable, and implemented consistently across all study participants?	X		
	10. Was the exposure(s) assessed more than once over time?		X	
	11. Were the outcome measures (dependent variables) clearly defined, valid, reliable, and implemented consistently across all study participants?	X		
	12. Were the outcome assessors blinded to the exposure status of participants?	X		
	13. Was loss to follow-up after baseline 20% or less?	X		
	14. Were key potential confounding variables measured and adjusted statistically for their impact on the relationship between exposure(s) and outcome(s)?	X		
	**Total**	12		
Li et al (54), 2022	1. Was the research question or objective in this paper clearly stated?	X		
	2. Was the study population clearly specified and defined?	X		
	3. Was the participation rate of eligible persons at least 50%?	X		
	4. Were all the subjects selected or recruited from the same or similar populations (including the same time period)? Were inclusion and exclusion criteria for being in the study prespecified and applied uniformly to all participants?	X		
	5. Was a sample size justification, power description, or variance and effect estimates provided?		X	
	6. For the analyses in this paper, were the exposure(s) of interest measured prior to the outcome(s) being measured?	X		
	7. Was the timeframe sufficient so that one could reasonably expect to see an association between exposure and outcome if it existed?	X		
	8. For exposures that can vary in amount or level, did the study examine different levels of the exposure as related to the outcome (e.g., categories of exposure, or exposure measured as continuous variable)?	X		
	9. Were the exposure measures (independent variables) clearly defined, valid, reliable, and implemented consistently across all study participants?	X		
	10. Was the exposure(s) assessed more than once over time?			X
	11. Were the outcome measures (dependent variables) clearly defined, valid, reliable, and implemented consistently across all study participants?	X		
	12. Were the outcome assessors blinded to the exposure status of participants?	X		
	13. Was loss to follow-up after baseline 20% or less?			X
	14. Were key potential confounding variables measured and adjusted statistically for their impact on the relationship between exposure(s) and outcome(s)?	X		
	**Total**	11		
Shan et al (56), 2023	1. Was the research question or objective in this paper clearly stated?	X		
	2. Was the study population clearly specified and defined?	X		
	3. Was the participation rate of eligible persons at least 50%?	X		
	4. Were all the subjects selected or recruited from the same or similar populations (including the same time period)? Were inclusion and exclusion criteria for being in the study prespecified and applied uniformly to all participants?	X		
	5. Was a sample size justification, power description, or variance and effect estimates provided?		X	
	6. For the analyses in this paper, were the exposure(s) of interest measured prior to the outcome(s) being measured?	X		
	7. Was the timeframe sufficient so that one could reasonably expect to see an association between exposure and outcome if it existed?	X		

Source	Criteria	Yes	No	Cannot determine, not applicable
	8. For exposures that can vary in amount or level, did the study examine different levels of the exposure as related to the outcome (e.g., categories of exposure, or exposure measured as continuous variable)?	X		
	9. Were the exposure measures (independent variables) clearly defined, valid, reliable, and implemented consistently across all study participants?	X		
	10. Was the exposure(s) assessed more than once over time?	X		
	11. Were the outcome measures (dependent variables) clearly defined, valid, reliable, and implemented consistently across all study participants?	X		
	12. Were the outcome assessors blinded to the exposure status of participants?	X		
	13. Was loss to follow-up after baseline 20% or less?	X		
	14. Were key potential confounding variables measured and adjusted statistically for their impact on the relationship between exposure(s) and outcome(s)?	X		
	**Total**	13		
Fraser et al (57), 1999	1. Was the research question or objective in this paper clearly stated?	X		
	2. Was the study population clearly specified and defined?	X		
	3. Was the participation rate of eligible persons at least 50%?	X		
	4. Were all the subjects selected or recruited from the same or similar populations (including the same time period)? Were inclusion and exclusion criteria for being in the study prespecified and applied uniformly to all participants?	X		
	5. Was a sample size justification, power description, or variance and effect estimates provided?		X	
	6. For the analyses in this paper, were the exposure(s) of interest measured prior to the outcome(s) being measured?	X		
	7. Was the timeframe sufficient so that one could reasonably expect to see an association between exposure and outcome if it existed?	X		
	8. For exposures that can vary in amount or level, did the study examine different levels of the exposure as related to the outcome (e.g., categories of exposure, or exposure measured as continuous variable)?	X		
	9. Were the exposure measures (independent variables) clearly defined, valid, reliable, and implemented consistently across all study participants?	X		
	10. Was the exposure(s) assessed more than once over time?		X	
	11. Were the outcome measures (dependent variables) clearly defined, valid, reliable, and implemented consistently across all study participants?	X		
	12. Were the outcome assessors blinded to the exposure status of participants?	X		
	13. Was loss to follow-up after baseline 20% or less?	X		
	14. Were key potential confounding variables measured and adjusted statistically for their impact on the relationship between exposure(s) and outcome(s)?		X	
	**Total**	11		
Key et al (58), 2009	1. Was the research question or objective in this paper clearly stated?	X		
	2. Was the study population clearly specified and defined?	X		
	3. Was the participation rate of eligible persons at least 50%?	X		
	4. Were all the subjects selected or recruited from the same or similar populations (including the same time period)? Were inclusion and exclusion criteria for being in the study prespecified and applied uniformly to all participants?	X		
	5. Was a sample size justification, power description, or variance and effect estimates provided?		X	
	6. For the analyses in this paper, were the exposure(s) of interest measured prior to the outcome(s) being measured?	X		
	7. Was the timeframe sufficient so that one could reasonably expect to see an association between exposure and outcome if it existed?	X		
	8. For exposures that can vary in amount or level, did the study examine different levels of the exposure as related to the outcome (e.g., categories of exposure, or exposure measured as continuous variable)?			X

Source	Criteria	Yes	No	Cannot determine, not applicable
	9. Were the exposure measures (independent variables) clearly defined, valid, reliable, and implemented consistently across all study participants?	X		
	10. Was the exposure(s) assessed more than once over time?		X	
	11. Were the outcome measures (dependent variables) clearly defined, valid, reliable, and implemented consistently across all study participants?	X		
	12. Were the outcome assessors blinded to the exposure status of participants?	X		
	13. Was loss to follow-up after baseline 20% or less?			X
	14. Were key potential confounding variables measured and adjusted statistically for their impact on the relationship between exposure(s) and outcome(s)?		X	
	**Total**	9		
Cade et al (59), 2010	1. Was the research question or objective in this paper clearly stated?	X		
	2. Was the study population clearly specified and defined?	X		
	3. Was the participation rate of eligible persons at least 50%?	X		
	4. Were all the subjects selected or recruited from the same or similar populations (including the same time period)? Were inclusion and exclusion criteria for being in the study prespecified and applied uniformly to all participants?	X		
	5. Was a sample size justification, power description, or variance and effect estimates provided?	X		
	6. For the analyses in this paper, were the exposure(s) of interest measured prior to the outcome(s) being measured?	X		
	7. Was the timeframe sufficient so that one could reasonably expect to see an association between exposure and outcome if it existed?	X		
	8. For exposures that can vary in amount or level, did the study examine different levels of the exposure as related to the outcome (e.g., categories of exposure, or exposure measured as continuous variable)?	X		
	9. Were the exposure measures (independent variables) clearly defined, valid, reliable, and implemented consistently across all study participants?	X		
	10. Was the exposure(s) assessed more than once over time?		X	
	11. Were the outcome measures (dependent variables) clearly defined, valid, reliable, and implemented consistently across all study participants?	X		
	12. Were the outcome assessors blinded to the exposure status of participants?	X		
	13. Was loss to follow-up after baseline 20% or less?			X
	14. Were key potential confounding variables measured and adjusted statistically for their impact on the relationship between exposure(s) and outcome(s)?	X		
	**Total**	12		
Tantamango -Bartley et al (60), 2013	1. Was the research question or objective in this paper clearly stated?	X		
	2. Was the study population clearly specified and defined?	X		
	3. Was the participation rate of eligible persons at least 50%?	X		
	4. Were all the subjects selected or recruited from the same or similar populations (including the same time period)? Were inclusion and exclusion criteria for being in the study prespecified and applied uniformly to all participants?	X		
	5. Was a sample size justification, power description, or variance and effect estimates provided?		X	
	6. For the analyses in this paper, were the exposure(s) of interest measured prior to the outcome(s) being measured?	X		
	7. Was the timeframe sufficient so that one could reasonably expect to see an association between exposure and outcome if it existed?	X		
	8. For exposures that can vary in amount or level, did the study examine different levels of the exposure as related to the outcome (e.g., categories of exposure, or exposure measured as continuous variable)?			X
	9. Were the exposure measures (independent variables) clearly defined, valid, reliable, and implemented consistently across all study participants?	X		
	10. Was the exposure(s) assessed more than once over time?		X	

Source	Criteria	Yes	No	Cannot determine, not applicable
	11. Were the outcome measures (dependent variables) clearly defined, valid, reliable, and implemented consistently across all study participants?	X		
	12. Were the outcome assessors blinded to the exposure status of participants?	X		
	13. Was loss to follow-up after baseline 20% or less?			X
	14. Were key potential confounding variables measured and adjusted statistically for their impact on the relationship between exposure(s) and outcome(s)?	X		
	**Total**	10		
Gilsing et al (61), 2015	1. Was the research question or objective in this paper clearly stated?	X		
	2. Was the study population clearly specified and defined?	X		
	3. Was the participation rate of eligible persons at least 50%?	X		
	4. Were all the subjects selected or recruited from the same or similar populations (including the same time period)? Were inclusion and exclusion criteria for being in the study prespecified and applied uniformly to all participants?	X		
	5. Was a sample size justification, power description, or variance and effect estimates provided?		X	
	6. For the analyses in this paper, were the exposure(s) of interest measured prior to the outcome(s) being measured?	X		
	7. Was the timeframe sufficient so that one could reasonably expect to see an association between exposure and outcome if it existed?	X		
	8. For exposures that can vary in amount or level, did the study examine different levels of the exposure as related to the outcome (e.g., categories of exposure, or exposure measured as continuous variable)?	X		
	9. Were the exposure measures (independent variables) clearly defined, valid, reliable, and implemented consistently across all study participants?	X		
	10. Was the exposure(s) assessed more than once over time?			
	11. Were the outcome measures (dependent variables) clearly defined, valid, reliable, and implemented consistently across all study participants?	X	X	
	12. Were the outcome assessors blinded to the exposure status of participants?	X		
	13. Was loss to follow-up after baseline 20% or less?	X		
	14. Were key potential confounding variables measured and adjusted statistically for their impact on the relationship between exposure(s) and outcome(s)?	X		
	**Total**	12		
Orlich et al (62), 2015	1. Was the research question or objective in this paper clearly stated?	X		
	2. Was the study population clearly specified and defined?	X		
	3. Was the participation rate of eligible persons at least 50%?	X		
	4. Were all the subjects selected or recruited from the same or similar populations (including the same time period)? Were inclusion and exclusion criteria for being in the study prespecified and applied uniformly to all participants?	X		
	5. Was a sample size justification, power description, or variance and effect estimates provided?		X	
	6. For the analyses in this paper, were the exposure(s) of interest measured prior to the outcome(s) being measured?	X		
	7. Was the timeframe sufficient so that one could reasonably expect to see an association between exposure and outcome if it existed?	X		
	8. For exposures that can vary in amount or level, did the study examine different levels of the exposure as related to the outcome (e.g., categories of exposure, or exposure measured as continuous variable)?	X		
	9. Were the exposure measures (independent variables) clearly defined, valid, reliable, and implemented consistently across all study participants?	X		
	10. Was the exposure(s) assessed more than once over time?		X	
	11. Were the outcome measures (dependent variables) clearly defined, valid, reliable, and implemented consistently across all study participants?	X		
	12. Were the outcome assessors blinded to the exposure status of participants?	X		

Source	Criteria	Yes	No	Cannot determine, not applicable
	13. Was loss to follow-up after baseline 20% or less?			X
	14. Were key potential confounding variables measured and adjusted statistically for their impact on the relationship between exposure(s) and outcome(s)?	X		
	**Total**	11		
Gilsing et al (63), 2016	1. Was the research question or objective in this paper clearly stated?	X		
	2. Was the study population clearly specified and defined?	X		
	3. Was the participation rate of eligible persons at least 50%?	X		
	4. Were all the subjects selected or recruited from the same or similar populations (including the same time period)? Were inclusion and exclusion criteria for being in the study prespecified and applied uniformly to all participants?	X		
	5. Was a sample size justification, power description, or variance and effect estimates provided?		X	
	6. For the analyses in this paper, were the exposure(s) of interest measured prior to the outcome(s) being measured?	X		
	7. Was the timeframe sufficient so that one could reasonably expect to see an association between exposure and outcome if it existed?	X		
	8. For exposures that can vary in amount or level, did the study examine different levels of the exposure as related to the outcome (e.g., categories of exposure, or exposure measured as continuous variable)?	X		
	9. Were the exposure measures (independent variables) clearly defined, valid, reliable, and implemented consistently across all study participants?	X		
	10. Was the exposure(s) assessed more than once over time?		X	
	11. Were the outcome measures (dependent variables) clearly defined, valid, reliable, and implemented consistently across all study participants?	X		
	12. Were the outcome assessors blinded to the exposure status of participants?	X		
	13. Was loss to follow-up after baseline 20% or less?	X		
	14. Were key potential confounding variables measured and adjusted statistically for their impact on the relationship between exposure(s) and outcome(s)?	X		
	**Total**	12		
Tantamango-Bartley et al (64), 2016	1. Was the research question or objective in this paper clearly stated?	X		
	2. Was the study population clearly specified and defined?	X		
	3. Was the participation rate of eligible persons at least 50%?			X
	4. Were all the subjects selected or recruited from the same or similar populations (including the same time period)? Were inclusion and exclusion criteria for being in the study prespecified and applied uniformly to all participants?	X		
	5. Was a sample size justification, power description, or variance and effect estimates provided?		X	
	6. For the analyses in this paper, were the exposure(s) of interest measured prior to the outcome(s) being measured?	X		
	7. Was the timeframe sufficient so that one could reasonably expect to see an association between exposure and outcome if it existed?	X		
	8. For exposures that can vary in amount or level, did the study examine different levels of the exposure as related to the outcome (e.g., categories of exposure, or exposure measured as continuous variable)?	X		
	9. Were the exposure measures (independent variables) clearly defined, valid, reliable, and implemented consistently across all study participants?	X		
	10. Was the exposure(s) assessed more than once over time?		X	
	11. Were the outcome measures (dependent variables) clearly defined, valid, reliable, and implemented consistently across all study participants?	X		
	12. Were the outcome assessors blinded to the exposure status of participants?	X		
	13. Was loss to follow-up after baseline 20% or less?			X
	14. Were key potential confounding variables measured and adjusted statistically for their impact on the relationship between exposure(s) and outcome(s)?	X		

Source	Criteria	Yes	No	Cannot determine, not applicable
	**Total**	10		
Penniecook-Sawyers et al (65), 2016	1. Was the research question or objective in this paper clearly stated?	X		
	2. Was the study population clearly specified and defined?	X		
	3. Was the participation rate of eligible persons at least 50%?	X		
	4. Were all the subjects selected or recruited from the same or similar populations (including the same time period)? Were inclusion and exclusion criteria			
	for being in the study prespecified and applied uniformly to all participants?	X		
	5. Was a sample size justification, power description, or variance and effect estimates provided?		X	
	6. For the analyses in this paper, were the exposure(s) of interest measured prior to the outcome(s) being measured?			
	7. Was the timeframe sufficient so that one could reasonably expect to see an association between exposure and outcome if it existed?	X		
	8. For exposures that can vary in amount or level, did the study examine different levels of the exposure as related to the outcome (e.g., categories of	X		
	exposure, or exposure measured as continuous variable)?	X		
	9. Were the exposure measures (independent variables) clearly defined, valid, reliable, and implemented consistently across all study participants?			
	10. Was the exposure(s) assessed more than once over time?	X	X	
	11. Were the outcome measures (dependent variables) clearly defined, valid, reliable, and implemented consistently across all study participants?			
	12. Were the outcome assessors blinded to the exposure status of participants?	X		
	13. Was loss to follow-up after baseline 20% or less?	X		X
	14. Were key potential confounding variables measured and adjusted statistically for their impact on the relationship between exposure(s) and outcome(s)?			
	**Total**	X		
		11		
Rada-Fernandez de Jauregui et al (66), 2018	1. Was the research question or objective in this paper clearly stated?	X		
	2. Was the study population clearly specified and defined?	X		
	3. Was the participation rate of eligible persons at least 50%?	X		
	4. Were all the subjects selected or recruited from the same or similar populations (including the same time period)? Were inclusion and exclusion criteria for being in the study prespecified and applied uniformly to all participants?	X		
	5. Was a sample size justification, power description, or variance and effect estimates provided?		X	
	6. For the analyses in this paper, were the exposure(s) of interest measured prior to the outcome(s) being measured?	X		
	7. Was the timeframe sufficient so that one could reasonably expect to see an association between exposure and outcome if it existed?	X		
	8. For exposures that can vary in amount or level, did the study examine different levels of the exposure as related to the outcome (e.g., categories of exposure, or exposure measured as continuous variable)?	X		
	9. Were the exposure measures (independent variables) clearly defined, valid, reliable, and implemented consistently across all study participants?	X		
	10. Was the exposure(s) assessed more than once over time?		X	
	11. Were the outcome measures (dependent variables) clearly defined, valid, reliable, and implemented consistently across all study participants?	X		
	12. Were the outcome assessors blinded to the exposure status of participants?	X		
	13. Was loss to follow-up after baseline 20% or less?			X
	14. Were key potential confounding variables measured and adjusted statistically for their impact on the relationship between exposure(s) and outcome(s)?	X		
	**Total**	11		

Source	Criteria	Yes	No	Cannot determine, not applicable
Kane-Diallo et al (67), 2018	1. Was the research question or objective in this paper clearly stated?	X		
	2. Was the study population clearly specified and defined?	X		
	3. Was the participation rate of eligible persons at least 50%?	X		
	4. Were all the subjects selected or recruited from the same or similar populations (including the same time period)? Were inclusion and exclusion criteria for being in the study prespecified and applied uniformly to all participants?	X		
	5. Was a sample size justification, power description, or variance and effect estimates provided?		X	
	6. For the analyses in this paper, were the exposure(s) of interest measured prior to the outcome(s) being measured?	X		
	7. Was the timeframe sufficient so that one could reasonably expect to see an association between exposure and outcome if it existed?	X		
	8. For exposures that can vary in amount or level, did the study examine different levels of the exposure as related to the outcome (e.g., categories of exposure, or exposure measured as continuous variable)?	X		
	9. Were the exposure measures (independent variables) clearly defined, valid, reliable, and implemented consistently across all study participants?	X		
	10. Was the exposure(s) assessed more than once over time?	X		
	11. Were the outcome measures (dependent variables) clearly defined, valid, reliable, and implemented consistently across all study participants?	X		
	12. Were the outcome assessors blinded to the exposure status of participants?	X		
	13. Was loss to follow-up after baseline 20% or less?			X
	14. Were key potential confounding variables measured and adjusted statistically for their impact on the relationship between exposure(s) and outcome(s)?	X		
	**Total**	12		
Leone et al (68), 2020	1. Was the research question or objective in this paper clearly stated?	X		
	2. Was the study population clearly specified and defined?	X		
	3. Was the participation rate of eligible persons at least 50%?		X	
	4. Were all the subjects selected or recruited from the same or similar populations (including the same time period)? Were inclusion and exclusion criteria for being in the study prespecified and applied uniformly to all participants?	X		
	5. Was a sample size justification, power description, or variance and effect estimates provided?		X	
	6. For the analyses in this paper, were the exposure(s) of interest measured prior to the outcome(s) being measured?	X		
	7. Was the timeframe sufficient so that one could reasonably expect to see an association between exposure and outcome if it existed?	X		
	8. For exposures that can vary in amount or level, did the study examine different levels of the exposure as related to the outcome (e.g., categories of exposure, or exposure measured as continuous variable)?	X		
	9. Were the exposure measures (independent variables) clearly defined, valid, reliable, and implemented consistently across all study participants?	X		
	10. Was the exposure(s) assessed more than once over time?		X	
	11. Were the outcome measures (dependent variables) clearly defined, valid, reliable, and implemented consistently across all study participants?	X		
	12. Were the outcome assessors blinded to the exposure status of participants?	X		
	13. Was loss to follow-up after baseline 20% or less?	X		
	14. Were key potential confounding variables measured and adjusted statistically for their impact on the relationship between exposure(s) and outcome(s)?	X		
	**Total**	11		

Source	Criteria	Yes	No	Cannot determine, not applicable
Romanos-Nanclares et al (69), 2020	1. Was the research question or objective in this paper clearly stated?	X		
	2. Was the study population clearly specified and defined?	X		
	3. Was the participation rate of eligible persons at least 50%?	X		
	4. Were all the subjects selected or recruited from the same or similar populations (including the same time period)? Were inclusion and exclusion criteria for being in the study prespecified and applied uniformly to all participants?	X		
	5. Was a sample size justification, power description, or variance and effect estimates provided?		X	
	6. For the analyses in this paper, were the exposure(s) of interest measured prior to the outcome(s) being measured?	X		
	7. Was the timeframe sufficient so that one could reasonably expect to see an association between exposure and outcome if it existed?	X		
	8. For exposures that can vary in amount or level, did the study examine different levels of the exposure as related to the outcome (e.g., categories of exposure, or exposure measured as continuous variable)?	X		
	9. Were the exposure measures (independent variables) clearly defined, valid, reliable, and implemented consistently across all study participants?	X		
	10. Was the exposure(s) assessed more than once over time?		X	
	11. Were the outcome measures (dependent variables) clearly defined, valid, reliable, and implemented consistently across all study participants?	X		
	12. Were the outcome assessors blinded to the exposure status of participants?	X		
	13. Was loss to follow-up after baseline 20% or less?	X		
	14. Were key potential confounding variables measured and adjusted statistically for their impact on the relationship between exposure(s) and outcome(s)?	X		
	**Total**	12		
Romanos-Nanclares et al (70), 2021	1. Was the research question or objective in this paper clearly stated?	X		
	2. Was the study population clearly specified and defined?	X		
	3. Was the participation rate of eligible persons at least 50%?	X		
	4. Were all the subjects selected or recruited from the same or similar populations (including the same time period)? Were inclusion and exclusion criteria for being in the study prespecified and applied uniformly to all participants?	X		
	5. Was a sample size justification, power description, or variance and effect estimates provided?		X	
	6. For the analyses in this paper, were the exposure(s) of interest measured prior to the outcome(s) being measured?	X		
	7. Was the timeframe sufficient so that one could reasonably expect to see an association between exposure and outcome if it existed?	X		
	8. For exposures that can vary in amount or level, did the study examine different levels of the exposure as related to the outcome (e.g., categories of exposure, or exposure measured as continuous variable)?	X		
	9. Were the exposure measures (independent variables) clearly defined, valid, reliable, and implemented consistently across all study participants?	X		
	10. Was the exposure(s) assessed more than once over time?	X		
	11. Were the outcome measures (dependent variables) clearly defined, valid, reliable, and implemented consistently across all study participants?	X		
	12. Were the outcome assessors blinded to the exposure status of participants?	X		
	13. Was loss to follow-up after baseline 20% or less?	X		
	14. Were key potential confounding variables measured and adjusted statistically for their impact on the relationship between exposure(s) and outcome(s)?	X		
	**Total**	13		
Kim et al (71), 2022	1. Was the research question or objective in this paper clearly stated?	X		
	2. Was the study population clearly specified and defined?	X		

Source	Criteria	Yes	No	Cannot determine, not applicable
(Multiethnic Cohort Study)	3. Was the participation rate of eligible persons at least 50%?	X		
	4. Were all the subjects selected or recruited from the same or similar populations (including the same time period)? Were inclusion and exclusion criteria for being in the study prespecified and applied uniformly to all participants?	X		
	5. Was a sample size justification, power description, or variance and effect estimates provided?		X	
	6. For the analyses in this paper, were the exposure(s) of interest measured prior to the outcome(s) being measured?	X		
	7. Was the timeframe sufficient so that one could reasonably expect to see an association between exposure and outcome if it existed?	X		
	8. For exposures that can vary in amount or level, did the study examine different levels of the exposure as related to the outcome (e.g., categories of exposure, or exposure measured as continuous variable)?	X		
	9. Were the exposure measures (independent variables) clearly defined, valid, reliable, and implemented consistently across all study participants?			
	10. Was the exposure(s) assessed more than once over time?	X		
	11. Were the outcome measures (dependent variables) clearly defined, valid, reliable, and implemented consistently across all study participants?		X	
	12. Were the outcome assessors blinded to the exposure status of participants?	X		
	13. Was loss to follow-up after baseline 20% or less?	X		
	14. Were key potential confounding variables measured and adjusted statistically for their impact on the relationship between exposure(s) and outcome(s)?			
	**Total**	X<br>11		X
Loeb et al (72), 2022	1. Was the research question or objective in this paper clearly stated?	X		
	2. Was the study population clearly specified and defined?	X		
	3. Was the participation rate of eligible persons at least 50%?	X		
	4. Were all the subjects selected or recruited from the same or similar populations (including the same time period)? Were inclusion and exclusion criteria for being in the study prespecified and applied uniformly to all participants?	X		
	5. Was a sample size justification, power description, or variance and effect estimates provided?		X	
	6. For the analyses in this paper, were the exposure(s) of interest measured prior to the outcome(s) being measured?	X		
	7. Was the timeframe sufficient so that one could reasonably expect to see an association between exposure and outcome if it existed?	X		
	8. For exposures that can vary in amount or level, did the study examine different levels of the exposure as related to the outcome (e.g., categories of exposure, or exposure measured as continuous variable)?	X		
	9. Were the exposure measures (independent variables) clearly defined, valid, reliable, and implemented consistently across all study participants?	X		
	10. Was the exposure(s) assessed more than once over time?	X		
	11. Were the outcome measures (dependent variables) clearly defined, valid, reliable, and implemented consistently across all study participants?	X		
	12. Were the outcome assessors blinded to the exposure status of participants?	X		
	13. Was loss to follow-up after baseline 20% or less?	X		
	14. Were key potential confounding variables measured and adjusted statistically for their impact on the relationship between exposure(s) and outcome(s)?	X		
	**Total**	13		
Kim et al (73), 2023 (Multiethnic	1. Was the research question or objective in this paper clearly stated?	X		
	2. Was the study population clearly specified and defined?	X		
	3. Was the participation rate of eligible persons at least 50%?	X		

Source	Criteria	Yes	No	Cannot determine, not applicable
Cohort Study)	4. Were all the subjects selected or recruited from the same or similar populations (including the same time period)? Were inclusion and exclusion criteria for being in the study prespecified and applied uniformly to all participants?	X		
	5. Was a sample size justification, power description, or variance and effect estimates provided?		X	
	6. For the analyses in this paper, were the exposure(s) of interest measured prior to the outcome(s) being measured?	X		
	7. Was the timeframe sufficient so that one could reasonably expect to see an association between exposure and outcome if it existed?	X		
	8. For exposures that can vary in amount or level, did the study examine different levels of the exposure as related to the outcome (e.g., categories of exposure, or exposure measured as continuous variable)?	X		
	9. Were the exposure measures (independent variables) clearly defined, valid, reliable, and implemented consistently across all study participants?	X		
	10. Was the exposure(s) assessed more than once over time?		X	
	11. Were the outcome measures (dependent variables) clearly defined, valid, reliable, and implemented consistently across all study participants?	X		
	12. Were the outcome assessors blinded to the exposure status of participants?	X		
	13. Was loss to follow-up after baseline 20% or less?	X		
	14. Were key potential confounding variables measured and adjusted statistically for their impact on the relationship between exposure(s) and outcome(s)?	X		
	**Total**	12		
Kim et al (74), 2023 (NHS, NHSII, HPFS)	1. Was the research question or objective in this paper clearly stated?	X		
	2. Was the study population clearly specified and defined?	X		
	3. Was the participation rate of eligible persons at least 50%?	X		
	4. Were all the subjects selected or recruited from the same or similar populations (including the same time period)? Were inclusion and exclusion criteria for being in the study prespecified and applied uniformly to all participants?	X		
	5. Was a sample size justification, power description, or variance and effect estimates provided?		X	
	6. For the analyses in this paper, were the exposure(s) of interest measured prior to the outcome(s) being measured?	X		
	7. Was the timeframe sufficient so that one could reasonably expect to see an association between exposure and outcome if it existed?	X		
	8. For exposures that can vary in amount or level, did the study examine different levels of the exposure as related to the outcome (e.g., categories of exposure, or exposure measured as continuous variable)?	X		
	9. Were the exposure measures (independent variables) clearly defined, valid, reliable, and implemented consistently across all study participants?	X		
	10. Was the exposure(s) assessed more than once over time?	X		
	11. Were the outcome measures (dependent variables) clearly defined, valid, reliable, and implemented consistently across all study participants?	X		
	12. Were the outcome assessors blinded to the exposure status of participants?	X		
	13. Was loss to follow-up after baseline 20% or less?	X		
	14. Were key potential confounding variables measured and adjusted statistically for their impact on the relationship between exposure(s) and outcome(s)?	X		
	**Total**	13		
Shah et al (75), 2022	1. Was the research question or objective in this paper clearly stated?	X		
	2. Was the study population clearly specified and defined?	X		
	3. Was the participation rate of eligible persons at least 50%?	X		
	4. Were all the subjects selected or recruited from the same or similar populations (including the same time period)? Were inclusion and exclusion criteria for being in the study prespecified and applied uniformly to all participants?	X		

Source	Criteria	Yes	No	Cannot determine, not applicable
	5. Was a sample size justification, power description, or variance and effect estimates provided?		X	
	6. For the analyses in this paper, were the exposure(s) of interest measured prior to the outcome(s) being measured?	X		
	7. Was the timeframe sufficient so that one could reasonably expect to see an association between exposure and outcome if it existed?	X		
	8. For exposures that can vary in amount or level, did the study examine different levels of the exposure as related to the outcome (e.g., categories of exposure, or exposure measured as continuous variable)?	X		
	9. Were the exposure measures (independent variables) clearly defined, valid, reliable, and implemented consistently across all study participants?	X		
	10. Was the exposure(s) assessed more than once over time?	X		
	11. Were the outcome measures (dependent variables) clearly defined, valid, reliable, and implemented consistently across all study participants?	X		
	12. Were the outcome assessors blinded to the exposure status of participants?	X		
	13. Was loss to follow-up after baseline 20% or less?	X		
	14. Were key potential confounding variables measured and adjusted statistically for their impact on the relationship between exposure(s) and outcome(s)?	X		
	**Total**	13		
Zhong et al (76), 2023	1. Was the research question or objective in this paper clearly stated?	X		
	2. Was the study population clearly specified and defined?	X		
	3. Was the participation rate of eligible persons at least 50%?	X		
	4. Were all the subjects selected or recruited from the same or similar populations (including the same time period)? Were inclusion and exclusion criteria for being in the study prespecified and applied uniformly to all participants?	X		
	5. Was a sample size justification, power description, or variance and effect estimates provided?		X	
	6. For the analyses in this paper, were the exposure(s) of interest measured prior to the outcome(s) being measured?	X		
	7. Was the timeframe sufficient so that one could reasonably expect to see an association between exposure and outcome if it existed?	X		
	8. For exposures that can vary in amount or level, did the study examine different levels of the exposure as related to the outcome (e.g., categories of exposure, or exposure measured as continuous variable)?	X		
	9. Were the exposure measures (independent variables) clearly defined, valid, reliable, and implemented consistently across all study participants?	X		
	10. Was the exposure(s) assessed more than once over time?		X	
	11. Were the outcome measures (dependent variables) clearly defined, valid, reliable, and implemented consistently across all study participants?	X		
	12. Were the outcome assessors blinded to the exposure status of participants?	X		
	13. Was loss to follow-up after baseline 20% or less?	X		
	14. Were key potential confounding variables measured and adjusted statistically for their impact on the relationship between exposure(s) and outcome(s)?	X		
	**Total**	12		

Supplemental Figure S1. Forest Plot of Studies Examining the Association Between Plant-Based Dietary Patterns and Risks of Specific Cardiovascular Disease using Random-Effects Meta-Analysis.

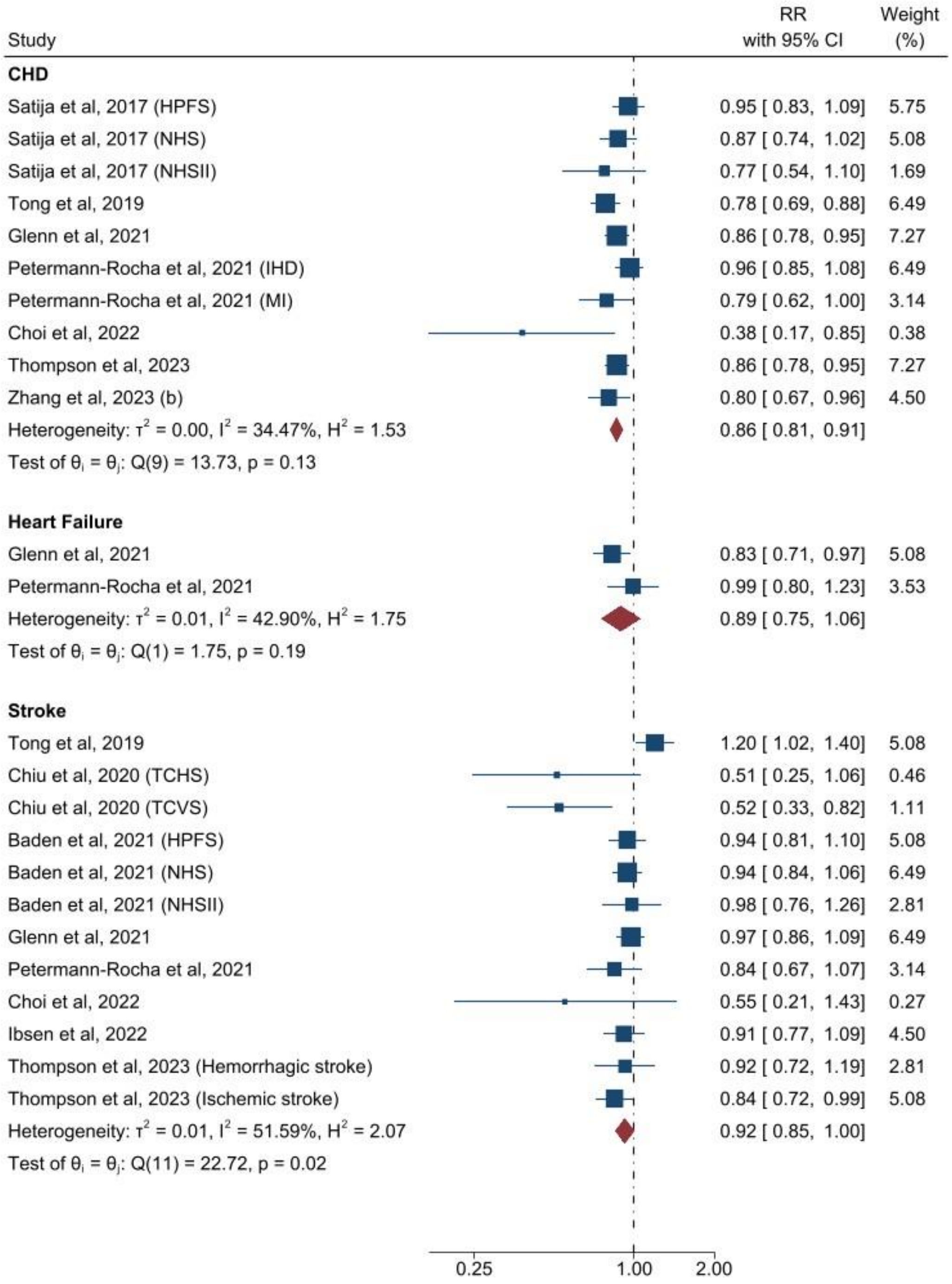

Abbreviations: IHD, ischemic heart disease; MI, myocardial infarction; TCHS, The Tzu Chi Health Study; TCVS, The Tzu Chi Vegetarian Study; HPFS, Health Professionals Follow-up Study; NHS, Nurses' Health Study; NHSII, Nurses' Health Study II.

Supplemental Figure S2. Forest Plot of Studies Examining the Association Between Plant-Based Dietary Patterns and Risks of Specific Cancer using Random-Effects Meta-Analysis.

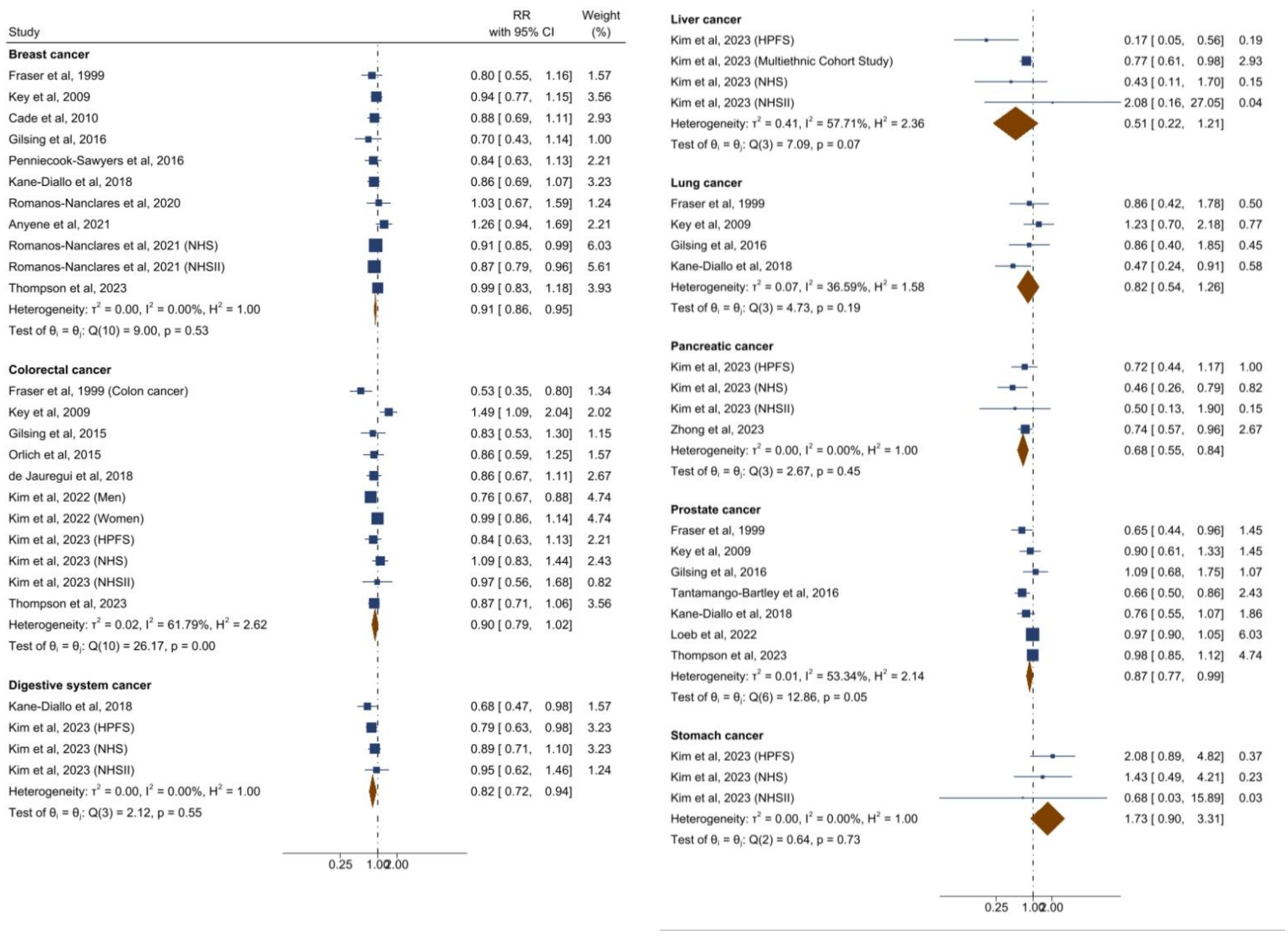

Abbreviations: NHS, Nurses' Health Study; NHSII, Nurses' Health Study II; HPFS: Health Professionals Follow-up Study.

Supplemental Figure S3. Forest Plot of Studies Examining the Association Between Plant-Based Dietary Patterns and Risks of Specific Mortality using Random-Effects Meta-Analysis.

(A) CVD mortality

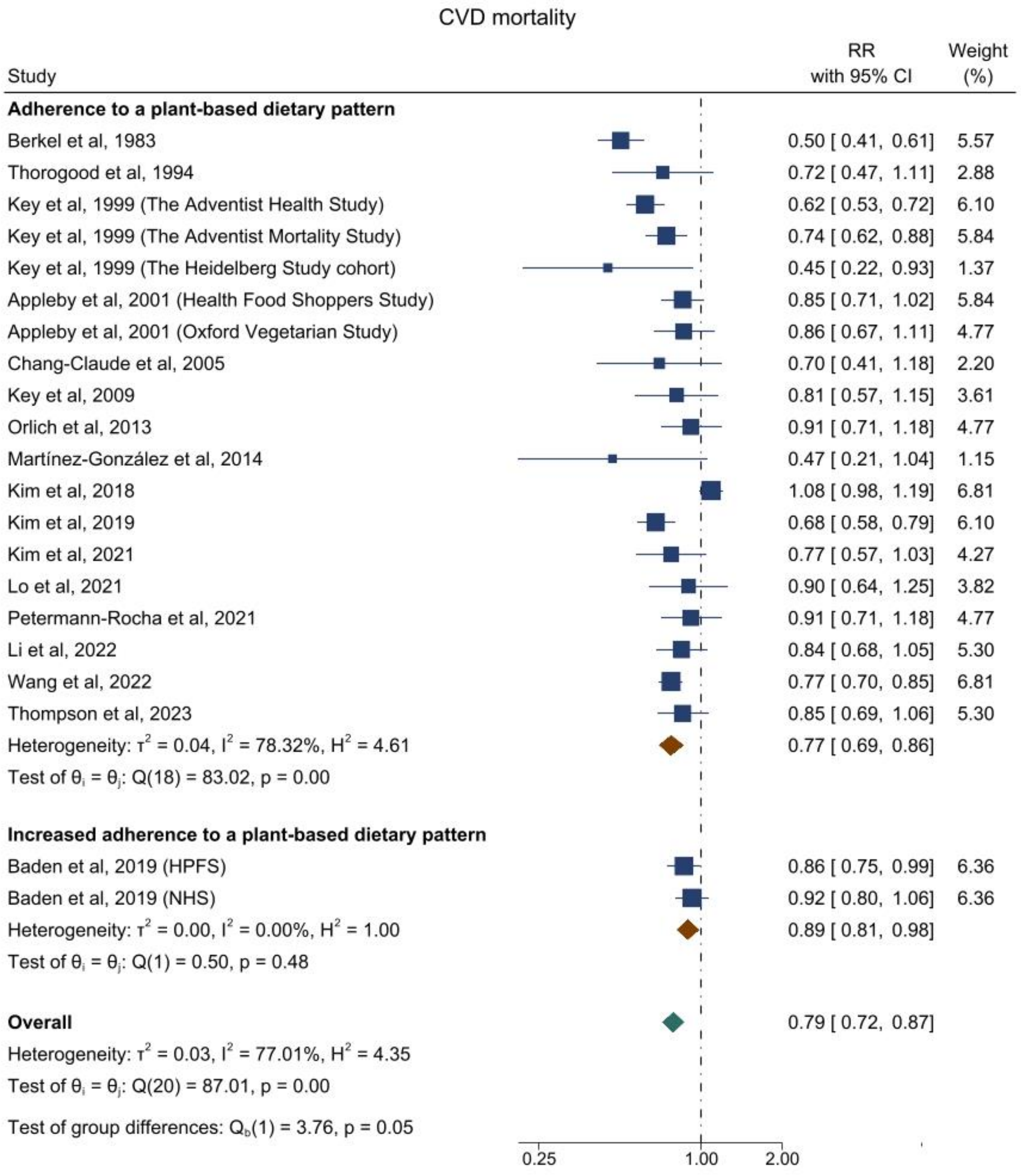

(B) Cancer mortality

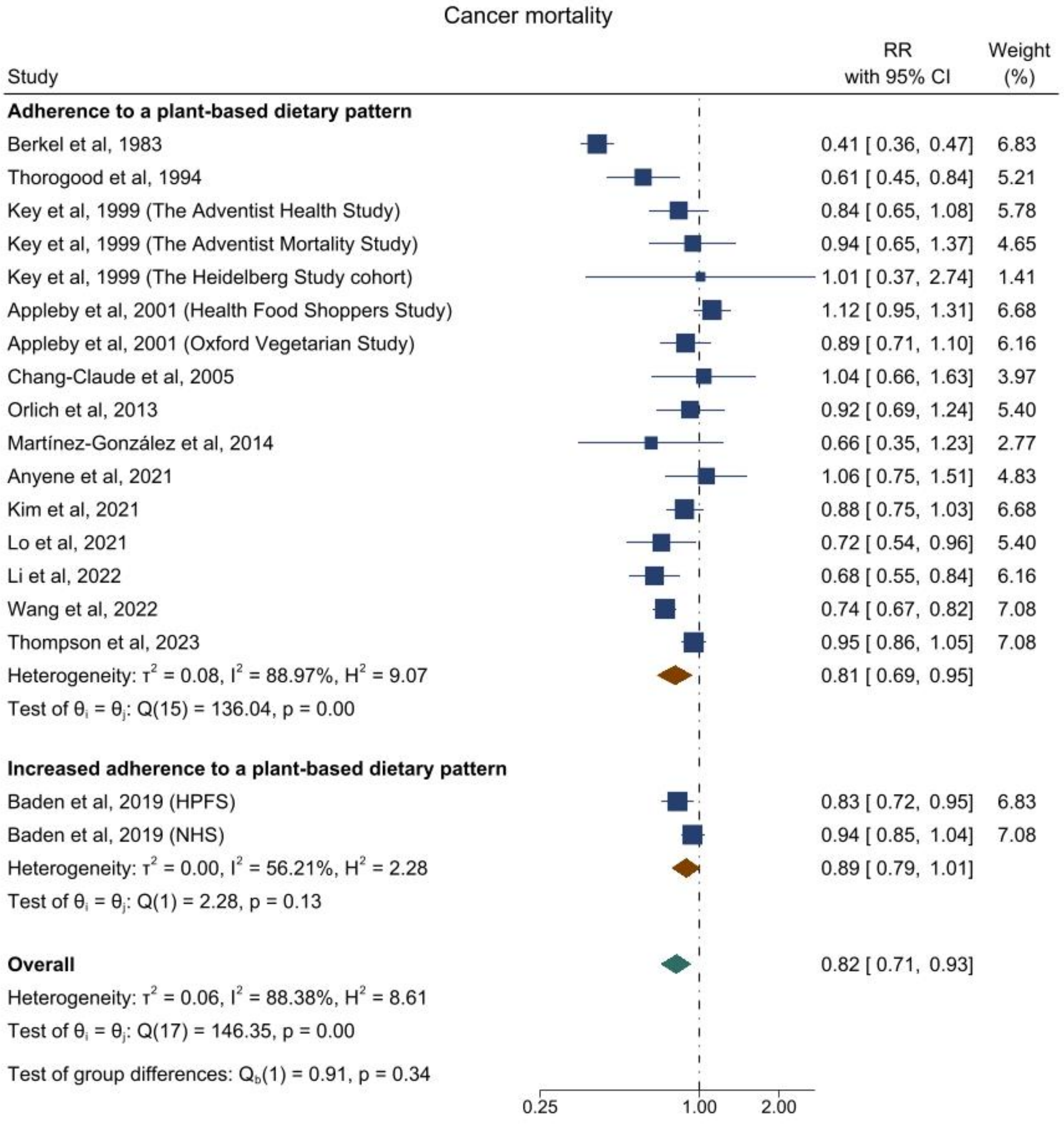

Abbreviations: HPFS, Health Professionals Follow-up Study; NHS, Nurses' Health Study.

Supplemental Figure S4. Forest Plot of Studies Examining the Association Between Plant-Based Dietary Patterns and Risks of Type 2 Diabetes using Inverse-Variance Fixed-Effects Meta-Analysis.

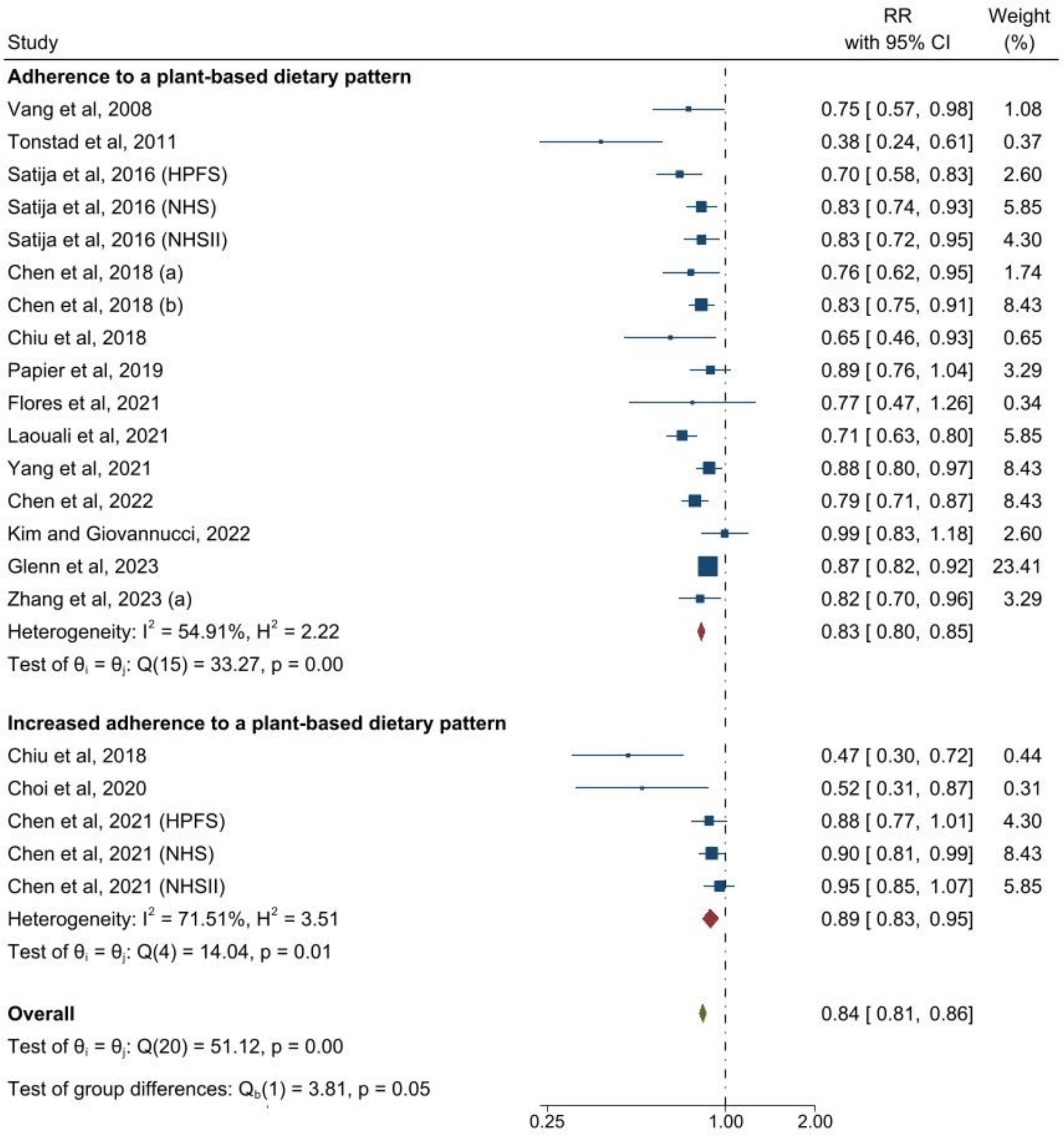

Abbreviations: HPFS, Health Professionals Follow-up Study; NHS, Nurses' Health Study; NHSII, Nurses' Health Study II.

Supplemental Figure S5. Forest Plot of Studies Examining the Association Between Plant-Based Dietary Patterns and Risks of Cardiovascular Disease using Inverse-Variance Fixed-Effects Meta-Analysis.

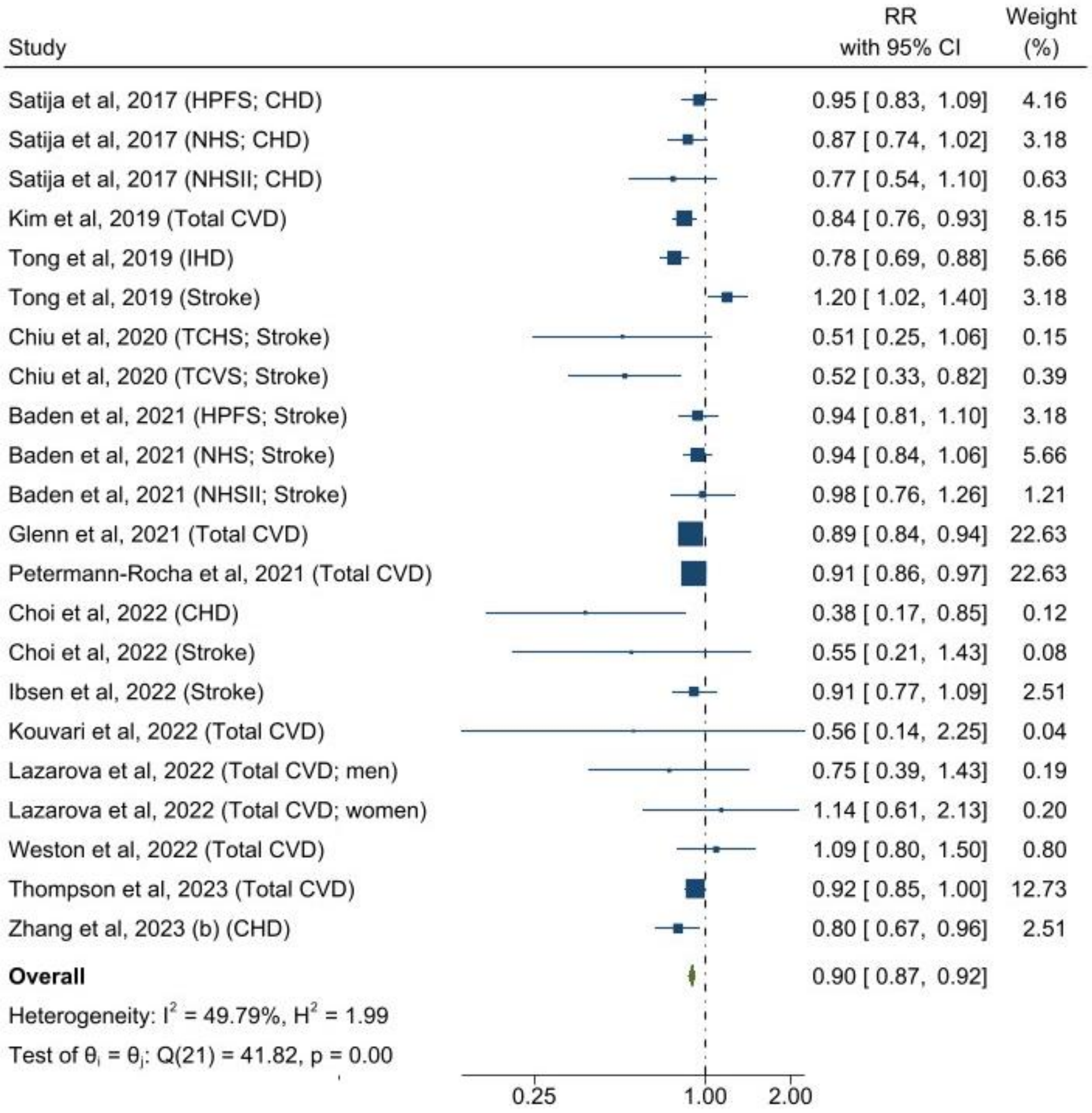

Abbreviations: CHD, coronary heart disease; CVD, cardiovascular disease; IHD, ischemic heart disease; HPFS, Health Professionals Follow-up Study; NHS, Nurses' Health Study; NHSII, Nurses' Health Study II; TCHS, The Tzu Chi Health Study; TCVS, The Tzu Chi Vegetarian Study.

Supplemental Figure S6. Forest Plot of Studies Examining the Association Between Plant-Based Dietary Patterns and Risks of Cancer using Inverse-Variance Fixed-Effects Meta-Analysis.

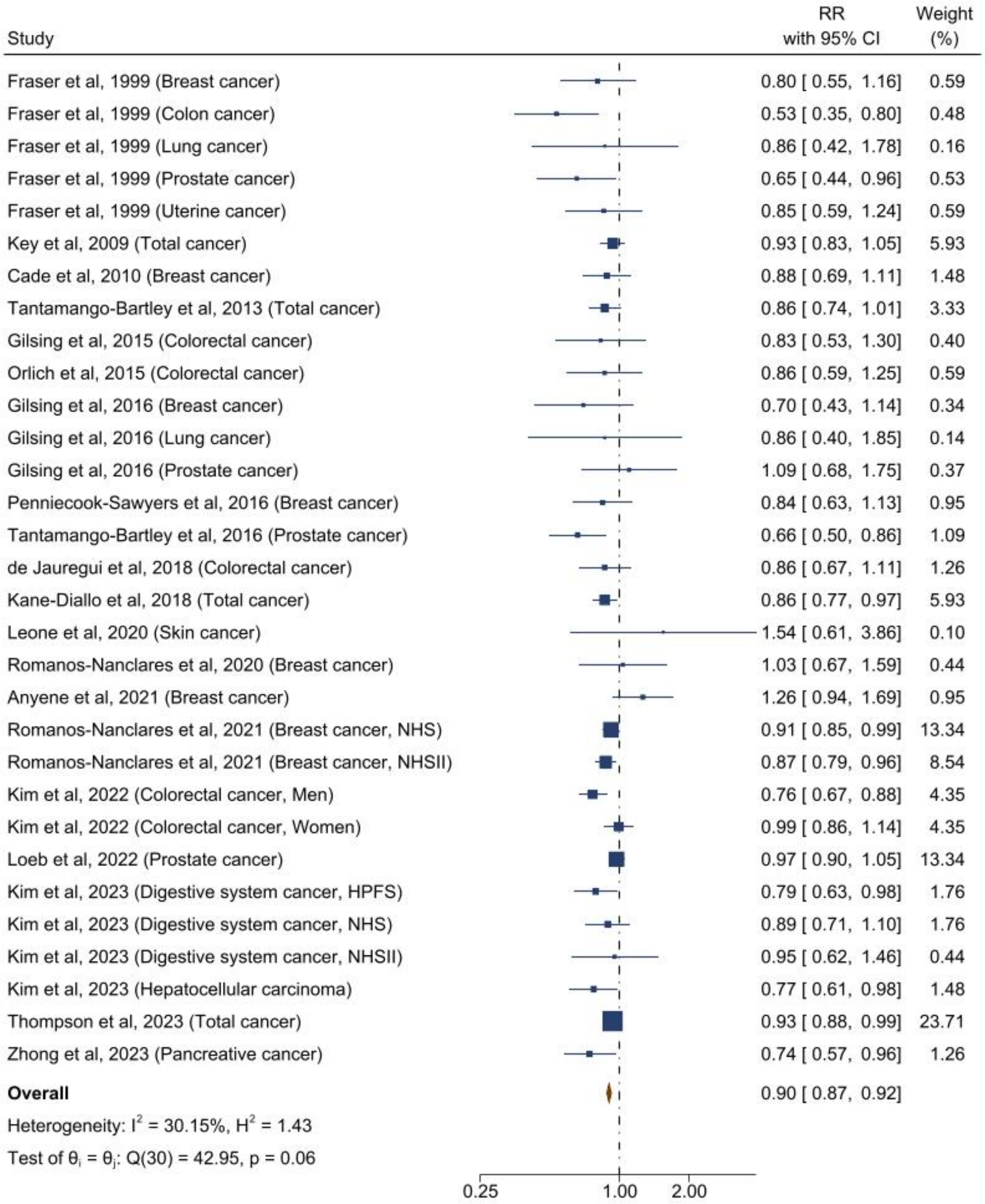

Abbreviations: NHS, Nurses' Health Study; NHSII, Nurses' Health Study II; HPFS, Health Professionals Follow-up Study.

Supplemental Figure S7. Forest Plot of Studies Examining the Association Between Plant-Based Dietary Patterns and Risks of Mortality using Inverse-Variance Fixed-Effects Meta-Analysis.

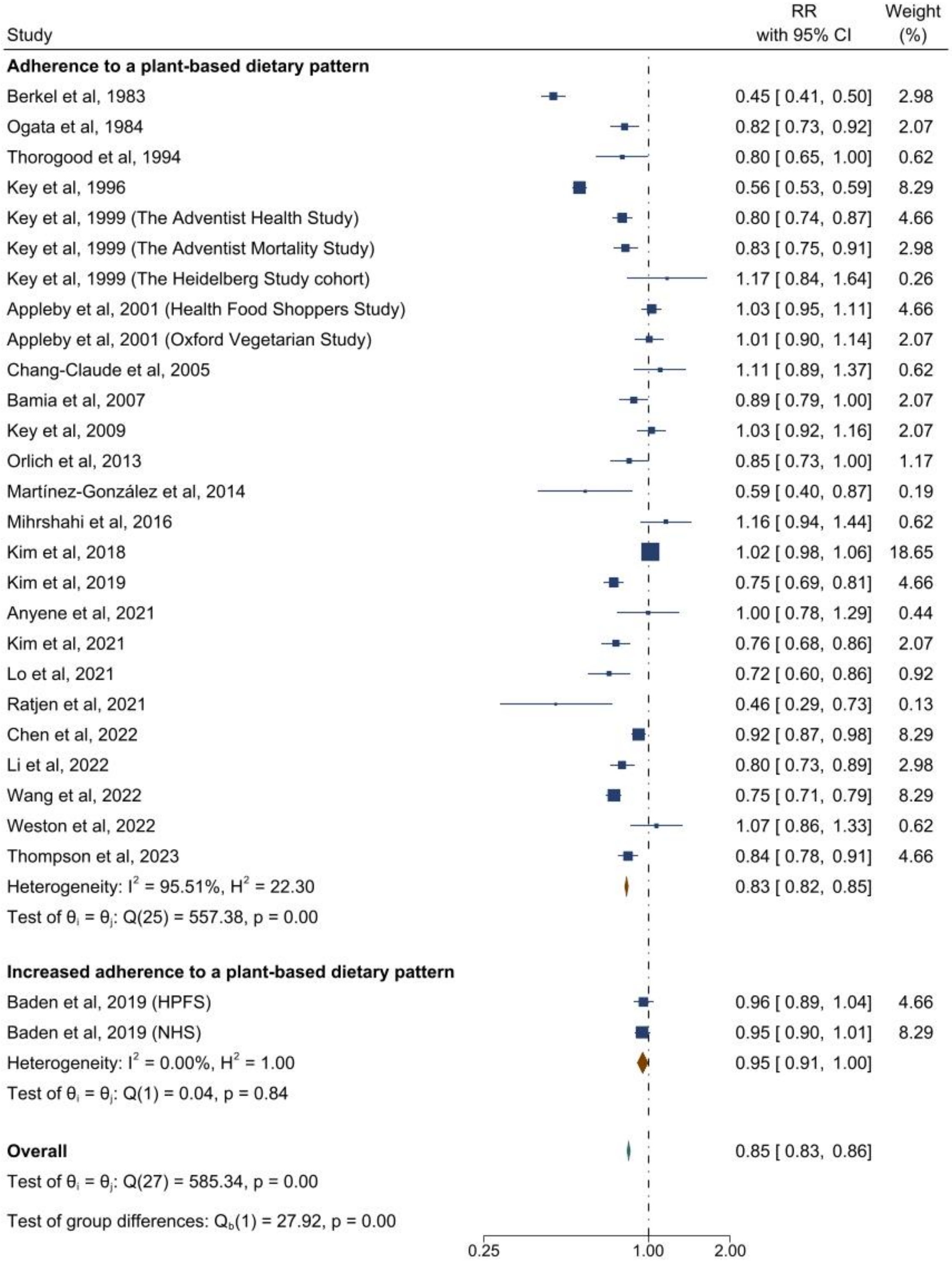

Abbreviations: HPFS, Health Professionals Follow-up Study; NHS, Nurses' Health Study.

Supplemental Figure S8. Changes to the Overall Association Between Plant-Based Dietary Patterns and Risks of Incident Type 2 Diabetes, Cardiovascular Disease, Cancer, and Mortality When Removing One Study at a time, Calculated Using Random-Effects Meta-Analysis.

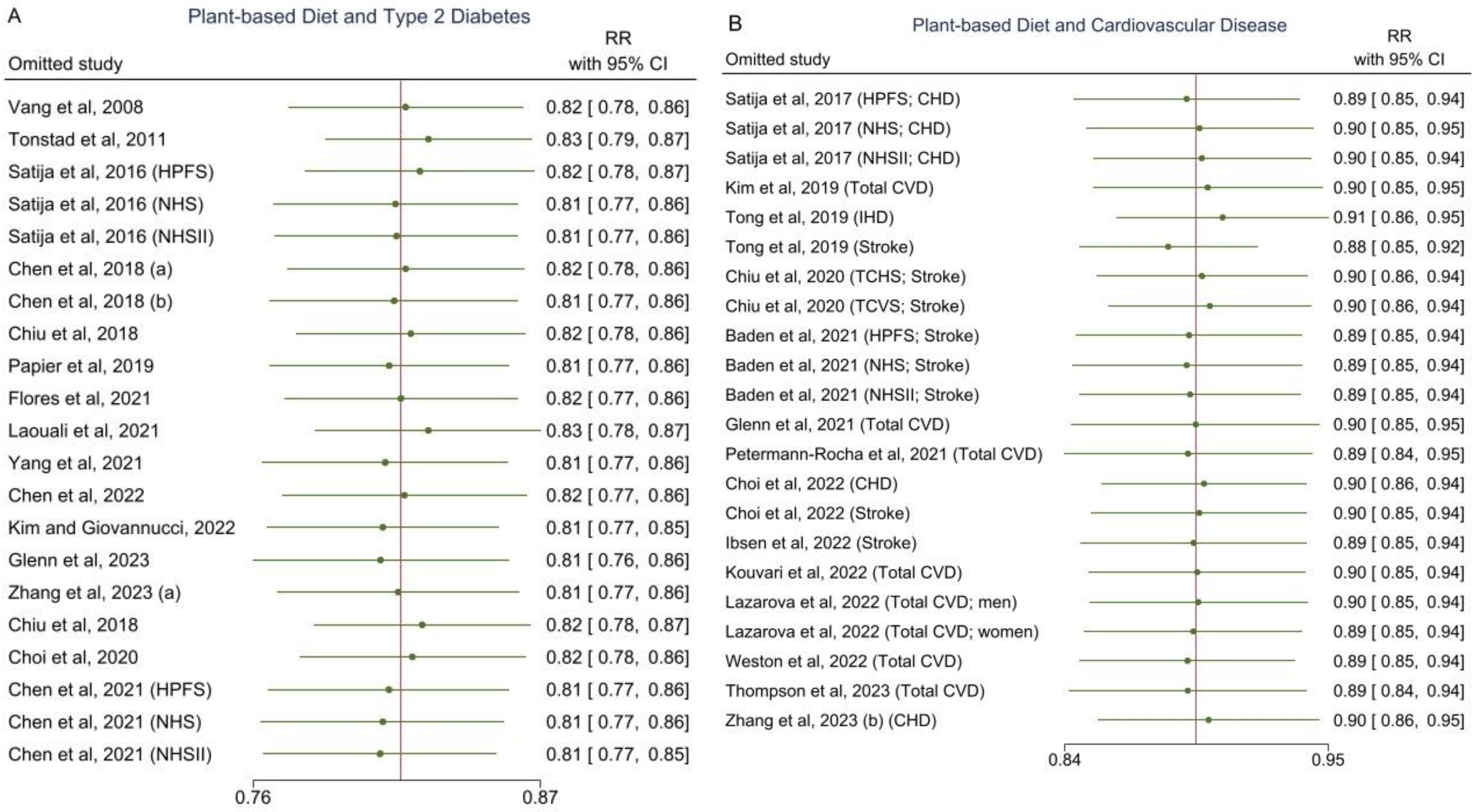

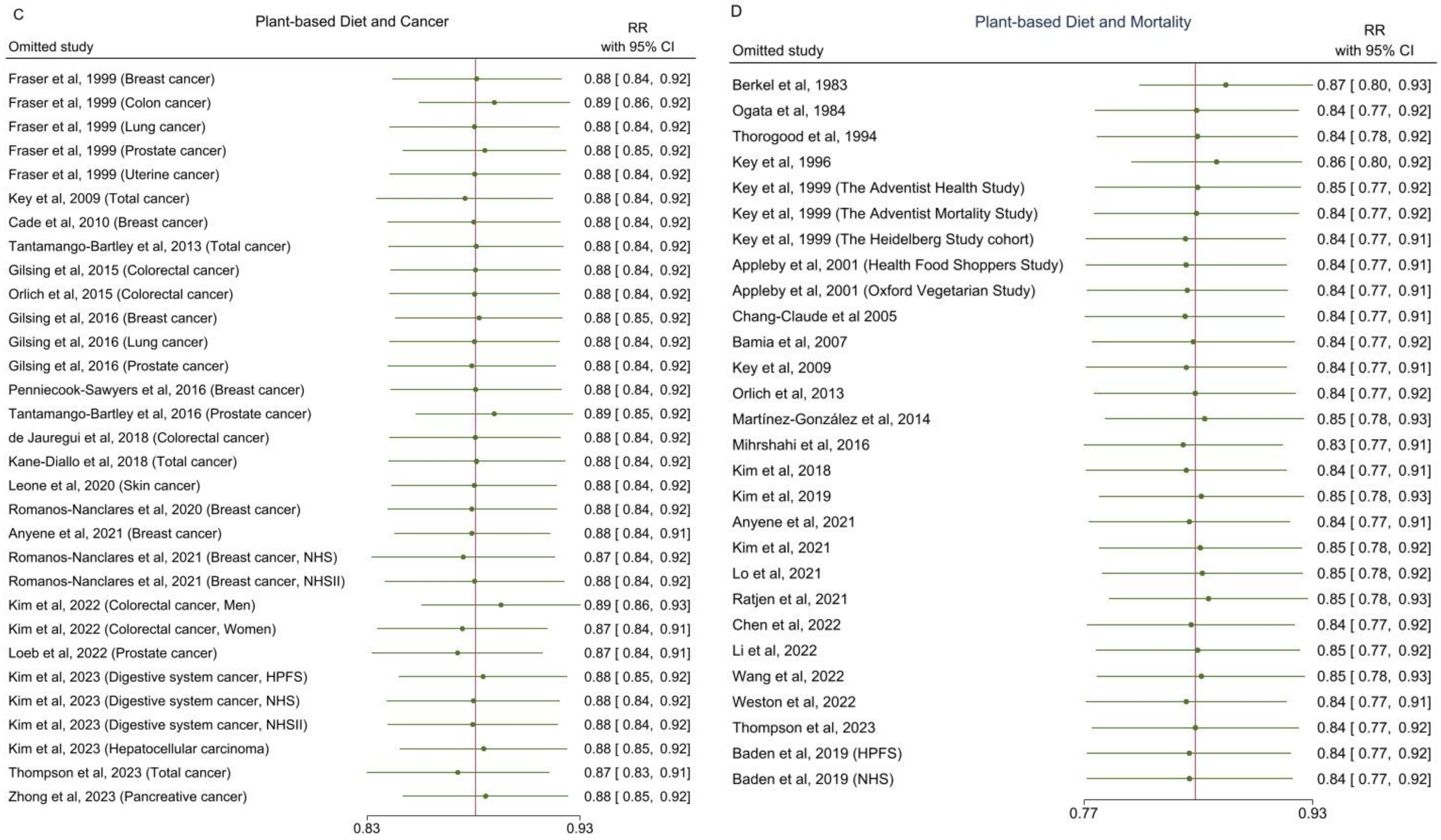

Abbreviations: CHD, coronary heart disease; CVD, cardiovascular disease; IHD, ischemic heart disease; HPFS, Health Professionals Follow-up Study; NHS, Nurses' Health Study; NHSII, Nurses' Health Study II; TCHS, The Tzu Chi Health Study; TCVS, The Tzu Chi Vegetarian Study.

Supplemental Figure S9. Forest Plot of Studies Examining the Association Between Healthful Plant-Based Dietary Patterns and Risks of Type 2 Diabetes, Cardiovascular Disease and subtypes, Cancer and subtypes, and Mortality and subtypes using Random-Effects Meta-Analysis.

(A) Type 2 Diabetes

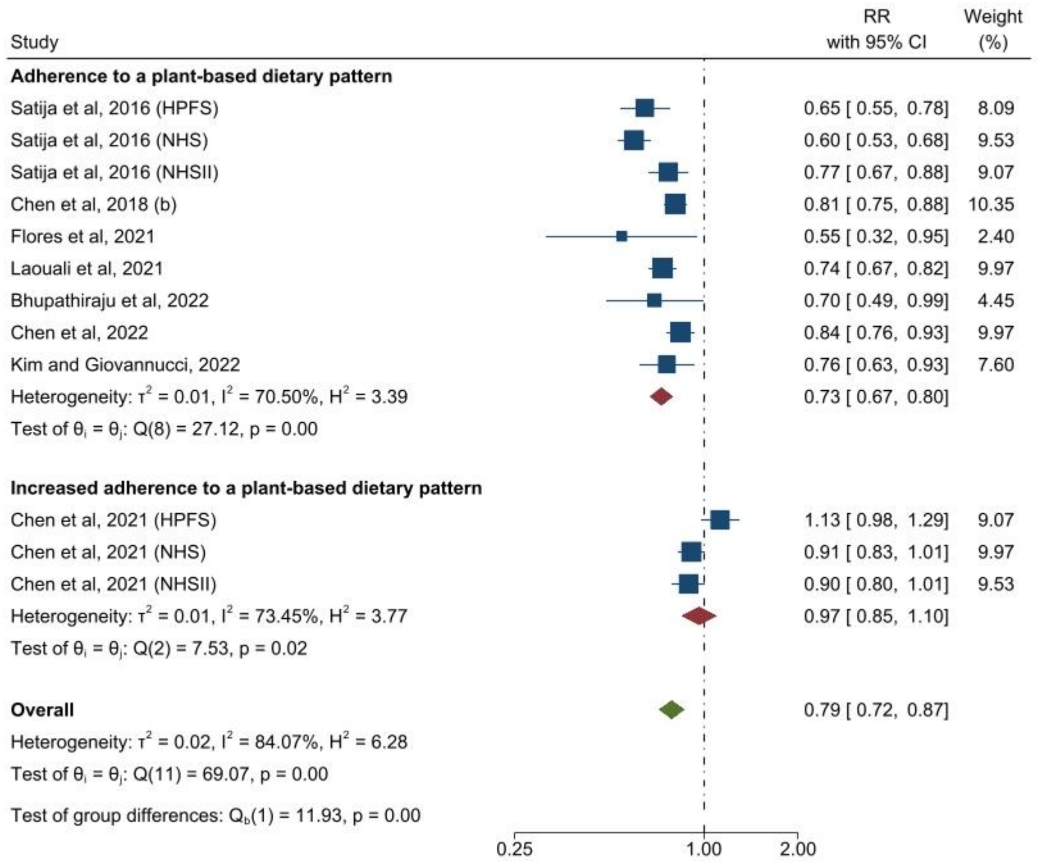

(B) CVD and subtypes

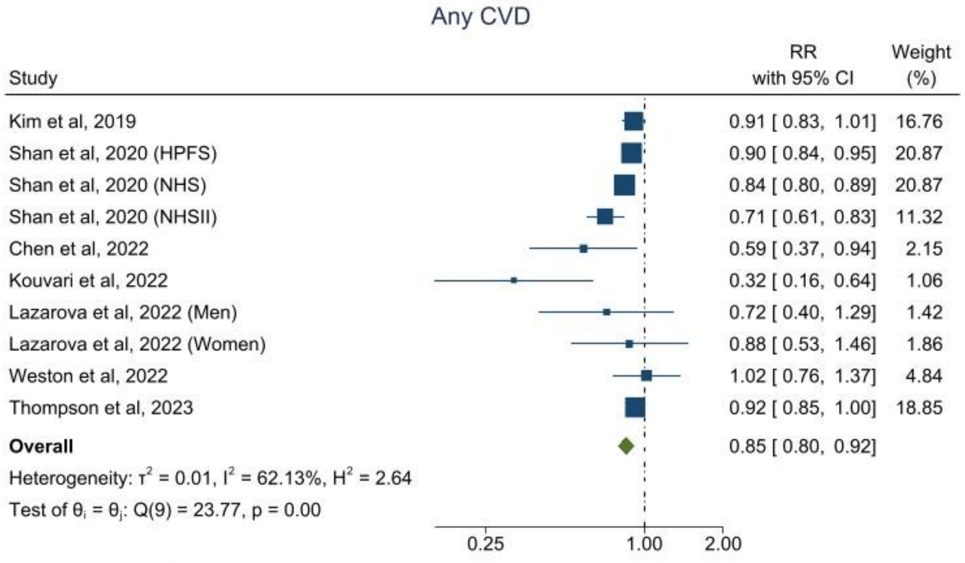

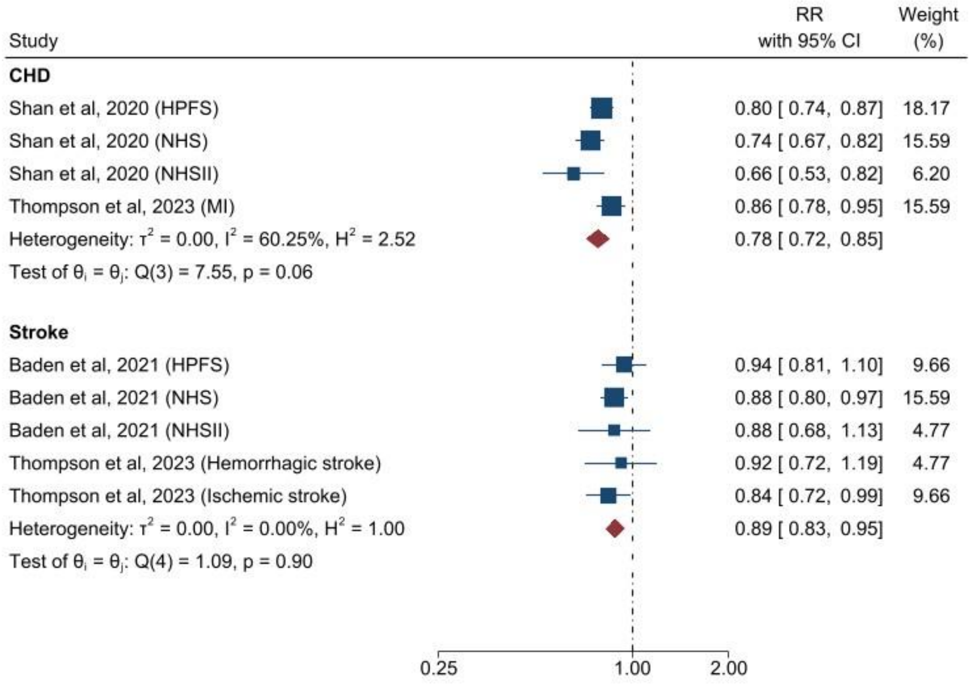

(C) Cancer and subtypes

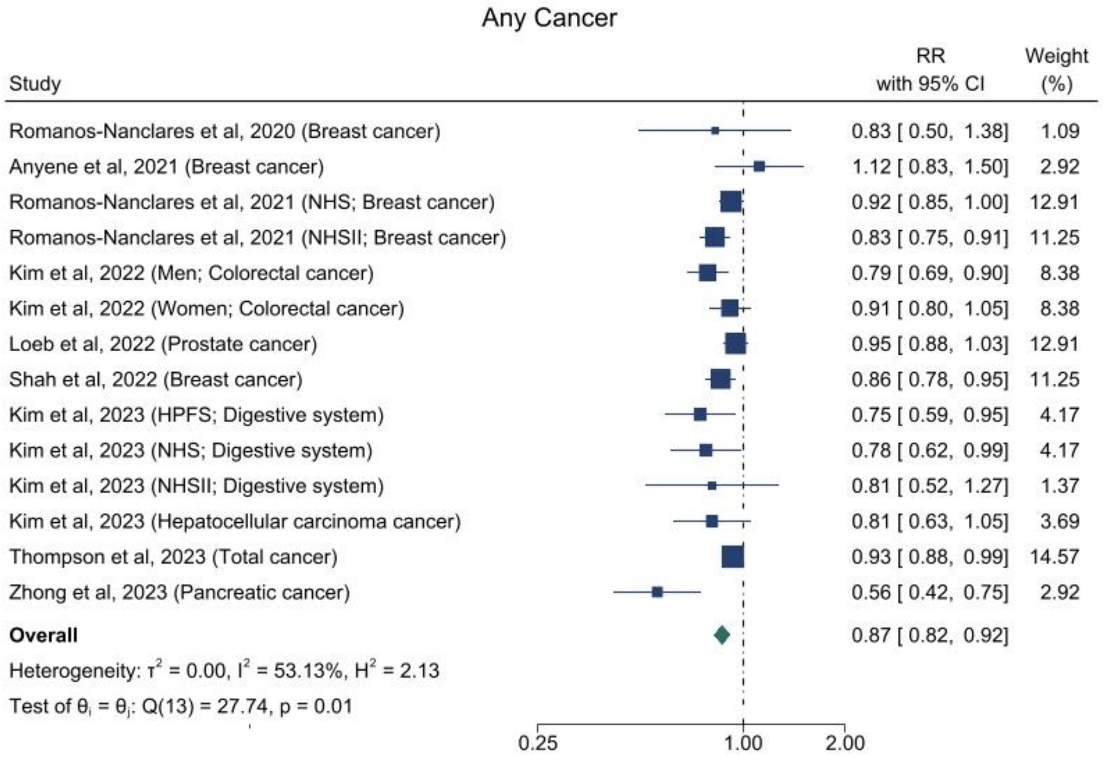

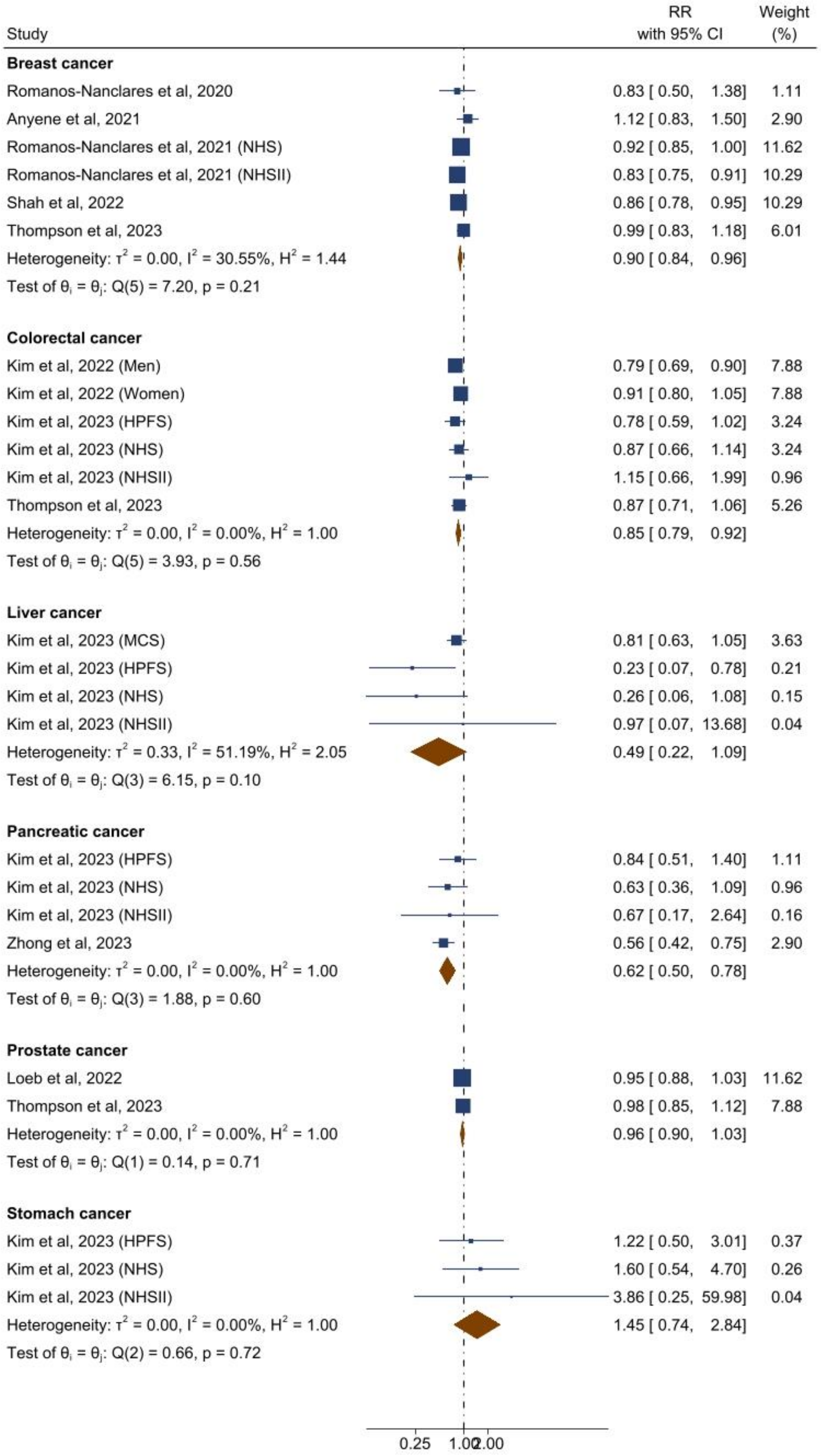
Study
RR with 95% CI
Weight (%)
Breast cancer
Romanos-Nanclares et al, 2020 0.83 [0.50, 1.38] 1.11
Anyene et al, 2021 1.12 [0.83, 1.50] 2.90
Romanos-Nanclares et al, 2021 (NHS) 0.92 [0.85, 1.00] 11.62
Romanos-Nanclares et al, 2021 (NHSII) 0.83 [0.75, 0.91] 10.29
Shah et al, 2022 0.86 [0.78, 0.95] 10.29
Thompson et al, 2023 0.99 [0.83, 1.18] 6.01
Heterogeneity: τ² = 0.00, I² = 30.55%, H² = 1.44 0.90 [0.84, 0.96]
Test of θi = θj: Q(5) = 7.20, p = 0.21
Colorectal cancer
Kim et al, 2022 (Men) 0.79 [0.69, 0.90] 7.88
Kim et al, 2022 (Women) 0.91 [0.80, 1.05] 7.88
Kim et al, 2023 (HPFS) 0.78 [0.59, 1.02] 3.24
Kim et al, 2023 (NHS) 0.87 [0.66, 1.14] 3.24
Kim et al, 2023 (NHSII) 1.15 [0.66, 1.99] 0.96
Thompson et al, 2023 0.87 [0.71, 1.06] 5.26
Heterogeneity: τ² = 0.00, I² = 0.00%, H² = 1.00 0.85 [0.79, 0.92]
Test of θi = θj: Q(5) = 3.93, p = 0.56
Liver cancer
Kim et al, 2023 (MCS) 0.81 [0.63, 1.05] 3.63
Kim et al, 2023 (HPFS) 0.23 [0.07, 0.78] 0.21
Kim et al, 2023 (NHS) 0.26 [0.06, 1.08] 0.15
Kim et al, 2023 (NHSII) 0.97 [0.07, 13.68] 0.04
Heterogeneity: τ² = 0.33, I² = 51.19%, H² = 2.05 0.49 [0.22, 1.09]
Test of θi = θj: Q(3) = 6.15, p = 0.10
Pancreatic cancer
Kim et al, 2023 (HPFS) 0.84 [0.51, 1.40] 1.11
Kim et al, 2023 (NHS) 0.63 [0.36, 1.09] 0.96
Kim et al, 2023 (NHSII) 0.67 [0.17, 2.64] 0.16
Zhong et al, 2023 0.56 [0.42, 0.75] 2.90
Heterogeneity: τ² = 0.00, I² = 0.00%, H² = 1.00 0.62 [0.50, 0.78]
Test of θi = θj: Q(3) = 1.88, p = 0.60
Prostate cancer
Loeb et al, 2022 0.95 [0.88, 1.03] 11.62
Thompson et al, 2023 0.98 [0.85, 1.12] 7.88
Heterogeneity: τ² = 0.00, I² = 0.00%, H² = 1.00 0.96 [0.90, 1.03]
Test of θi = θj: Q(1) = 0.14, p = 0.71
Stomach cancer
Kim et al, 2023 (HPFS) 1.22 [0.50, 3.01] 0.37
Kim et al, 2023 (NHS) 1.60 [0.54, 4.70] 0.26
Kim et al, 2023 (NHSII) 3.86 [0.25, 59.98] 0.04
Heterogeneity: τ² = 0.00, I² = 0.00%, H² = 1.00 1.45 [0.74, 2.84]
Test of θi = θj: Q(2) = 0.66, p = 0.72
0.25
1.00
2.00

(D) Mortality and subtypes

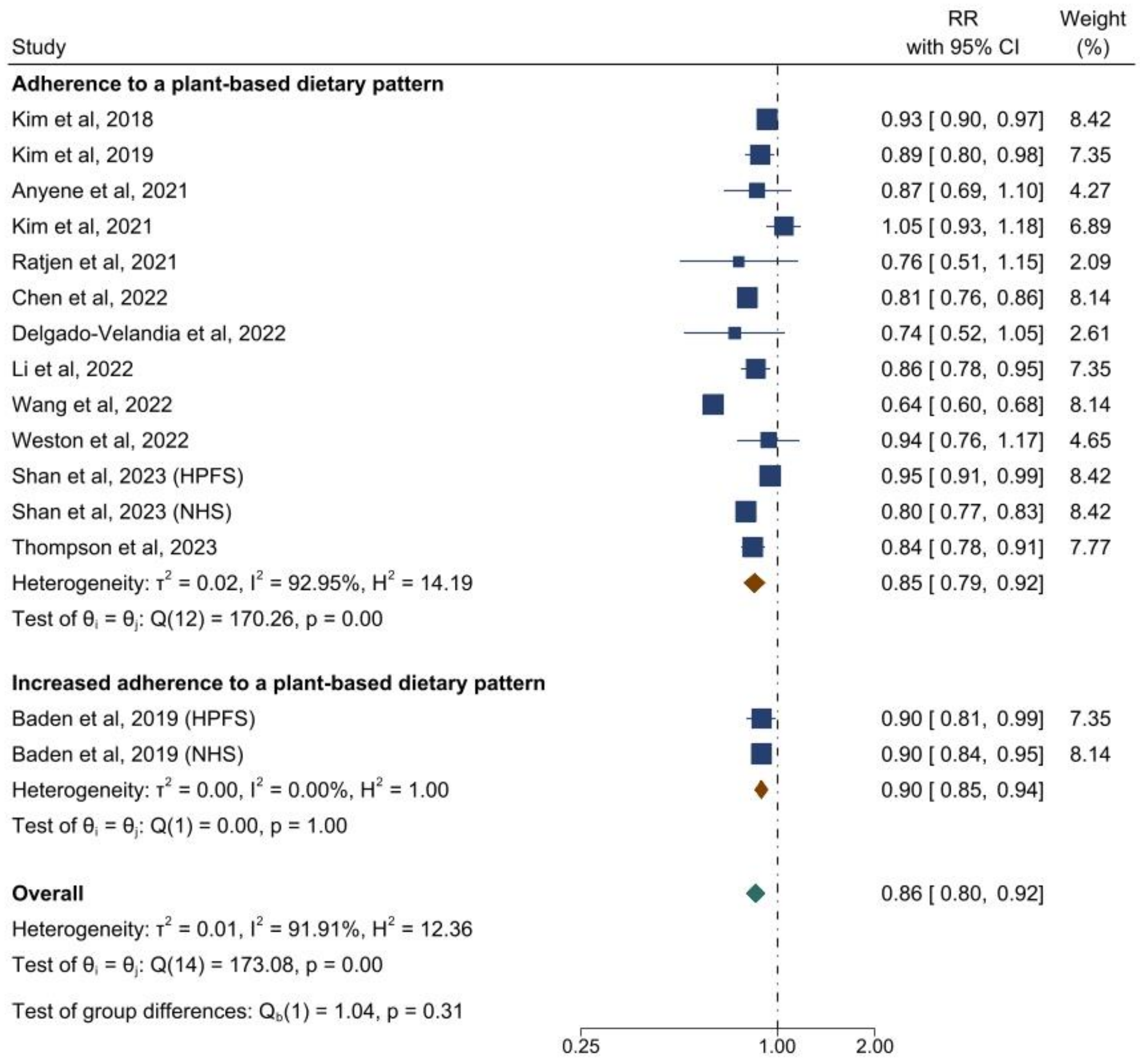

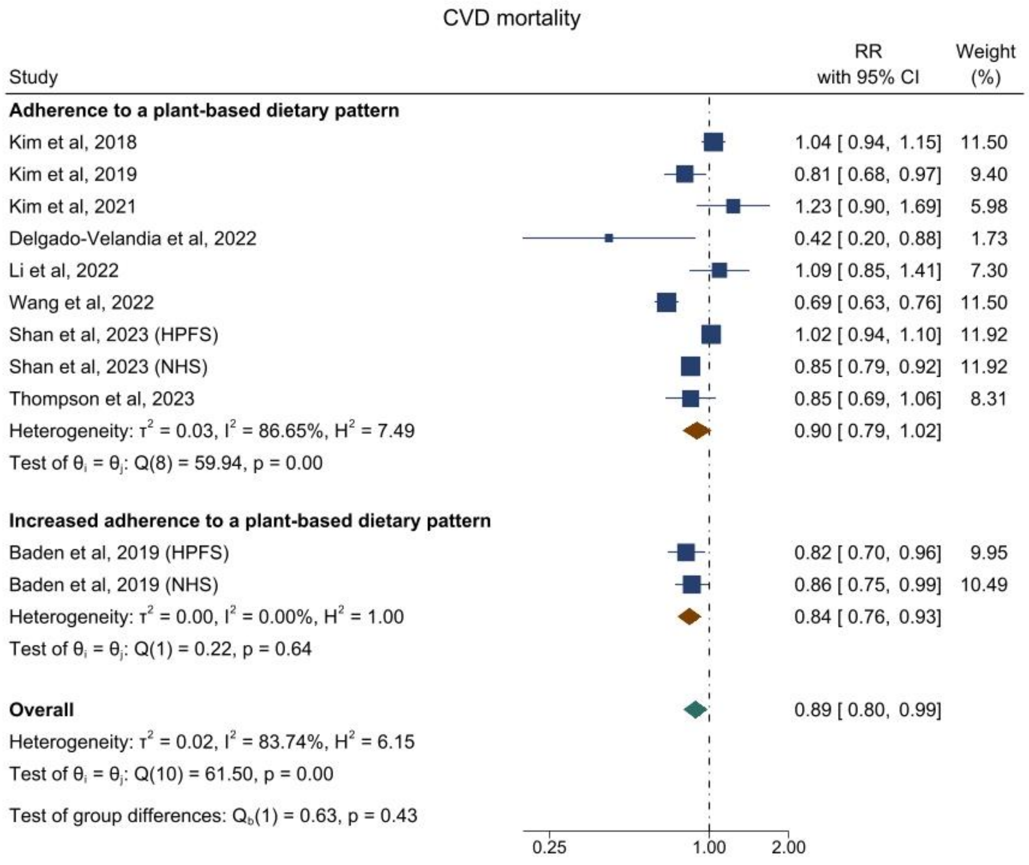

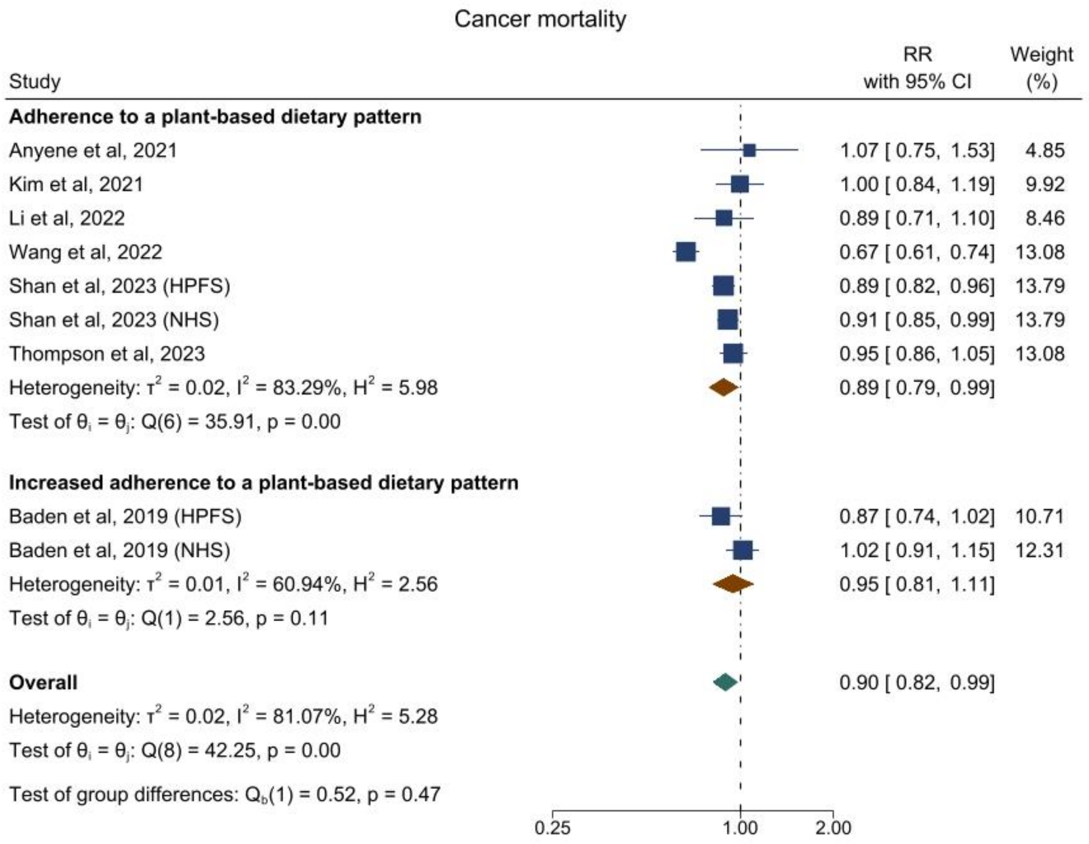

Abbreviations: HPFS, Health Professionals Follow-up Study; NHS, Nurses' Health Study; NHSII, Nurses' Health Study II; MCS, Multiethnic Cohort Study; CVD, cardiovascular disease; MI, myocardial infarction.

Supplemental Figure S10. Forest Plot of Studies Examining the Association Between Unhealthful Plant-Based Dietary Patterns and Risks of Type 2 Diabetes, Cardiovascular Disease and subtypes, Cancer and subtypes, and Mortality and subtypes using Random-Effects Meta-Analysis.

(A) Type 2 Diabetes

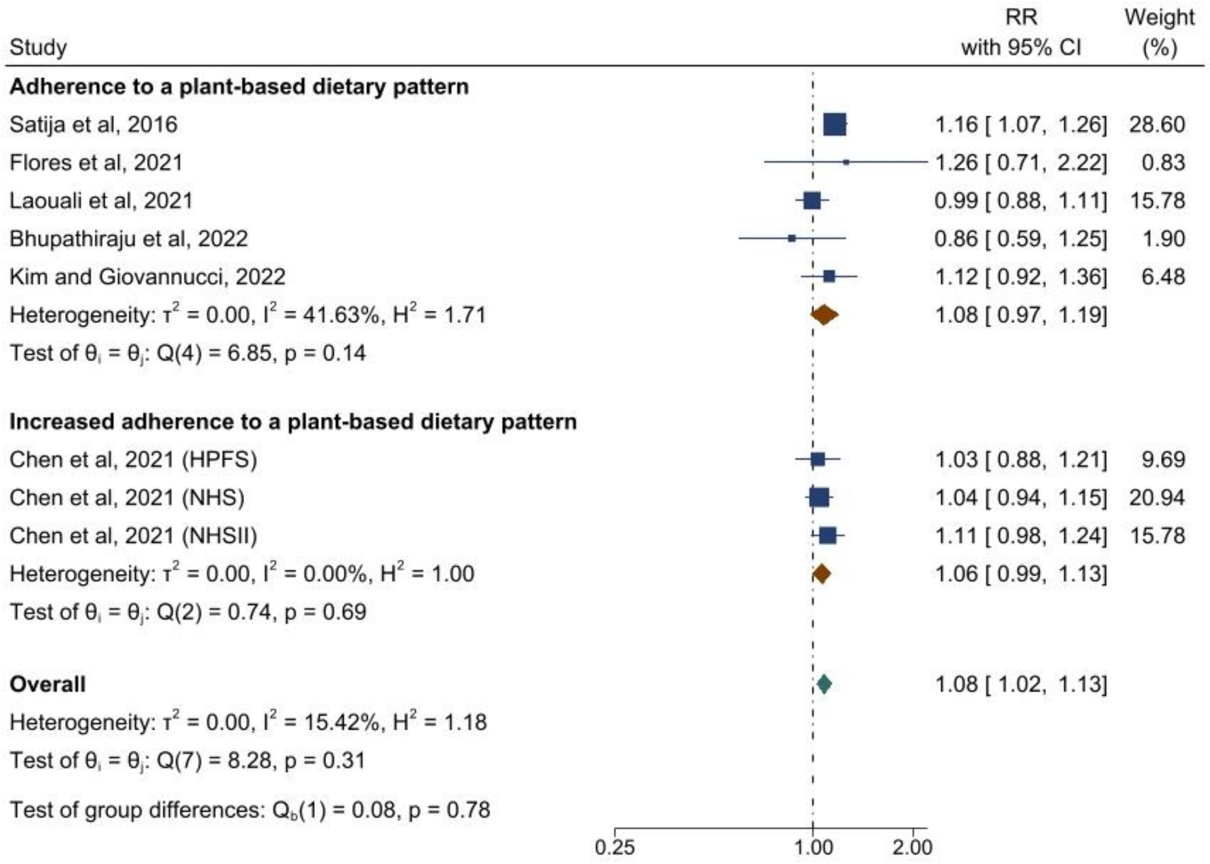

(B) CVD and subtypes

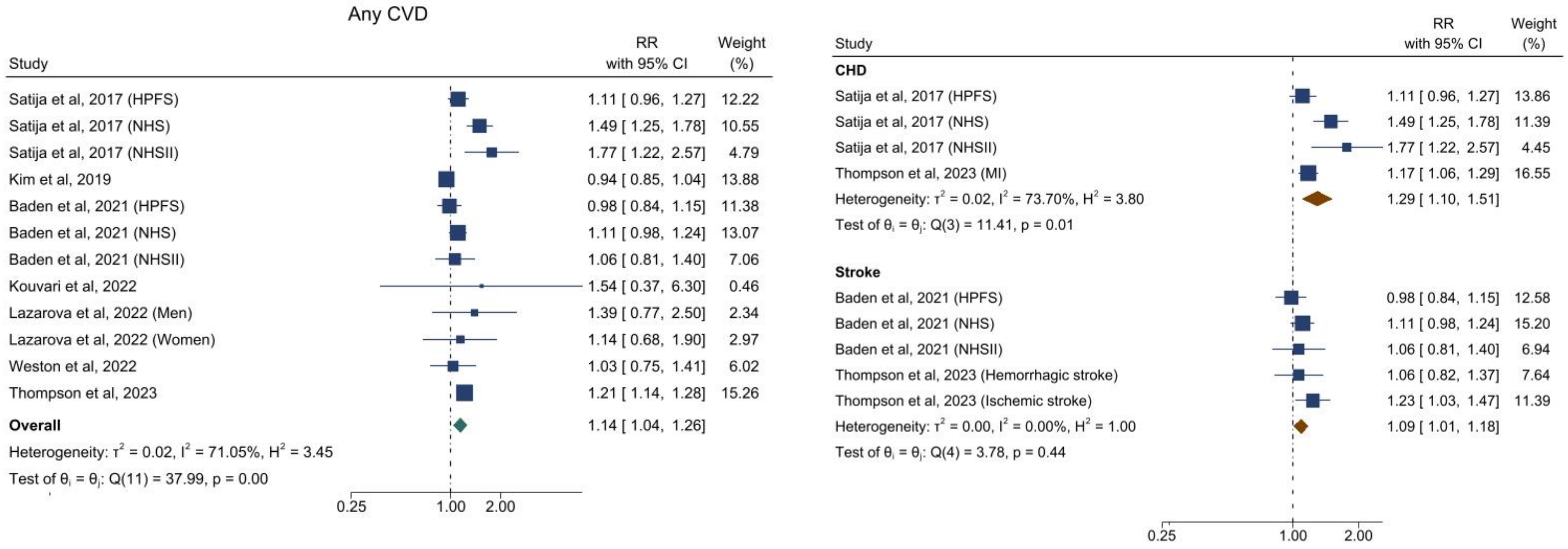

(C) Cancer and subtypes

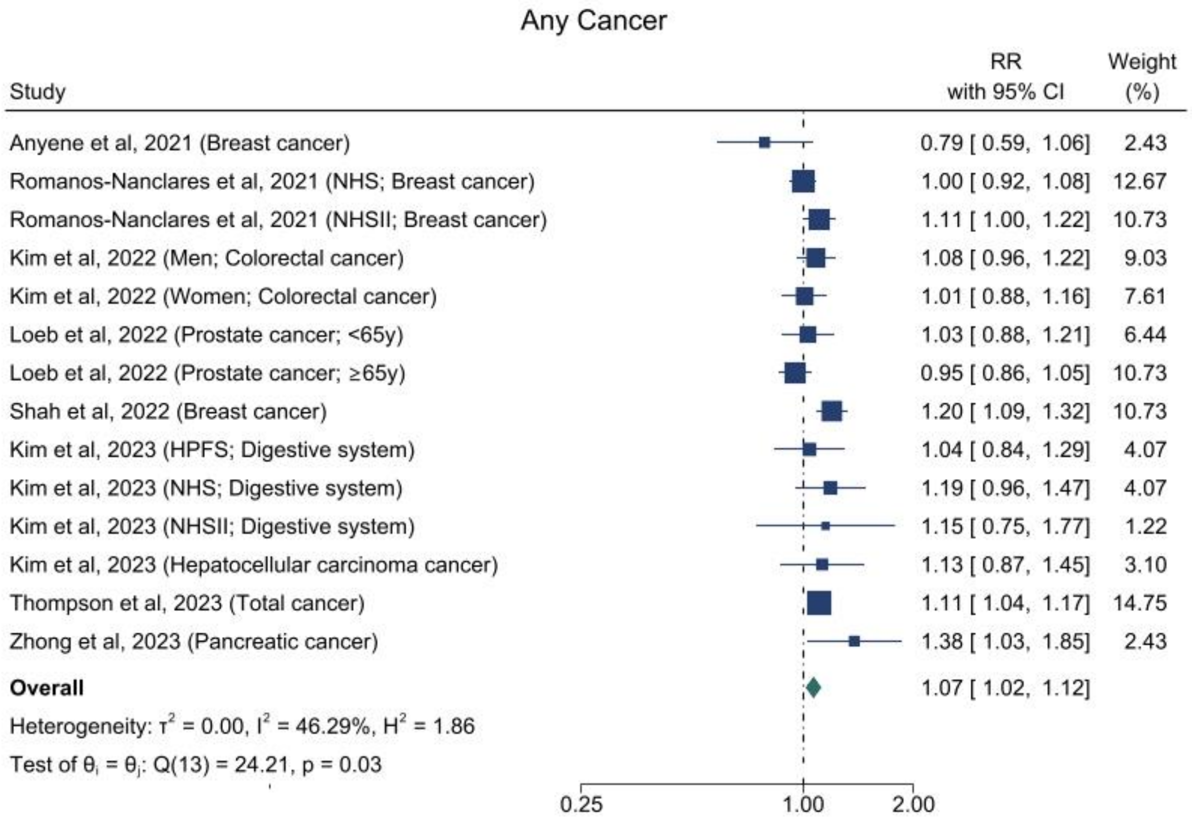

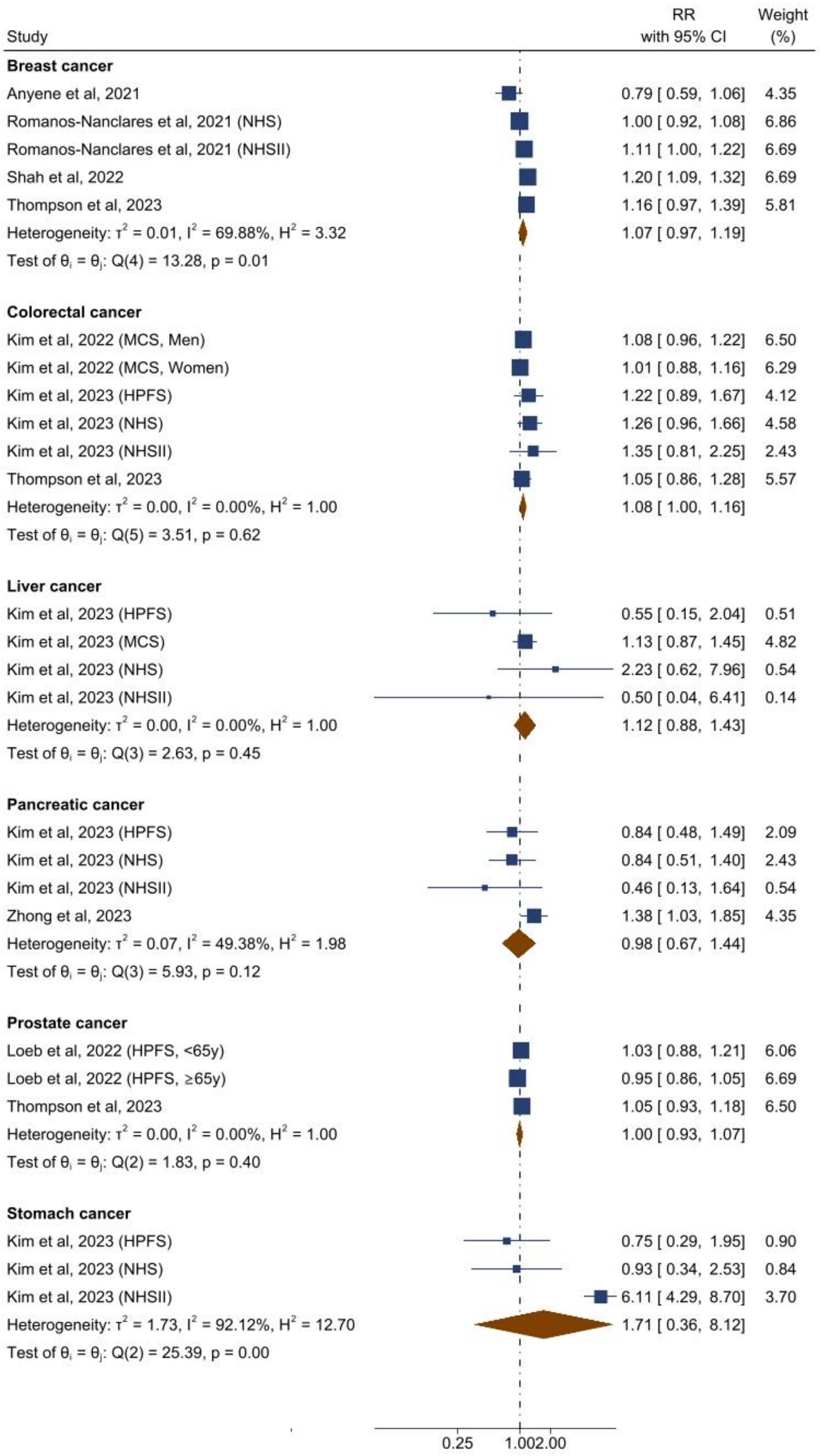
Study
RR with 95% CI
Weight (%)
Breast cancer
Anyene et al, 2021 0.79 [0.59, 1.06] 4.35
Romanos-Nanclares et al, 2021 (NHS) 1.00 [0.92, 1.08] 6.86
Romanos-Nanclares et al, 2021 (NHSII) 1.11 [1.00, 1.22] 6.69
Shah et al, 2022 1.20 [1.09, 1.32] 6.69
Thompson et al, 2023 1.16 [0.97, 1.39] 5.81
Heterogeneity: τ^2 = 0.01, I^2 = 69.88%, H^2 = 3.32 1.07 [0.97, 1.19]
Test of $\theta_i = \theta_j$: Q(4) = 13.28, p = 0.01
Colorectal cancer
Kim et al, 2022 (MCS, Men) 1.08 [0.96, 1.22] 6.50
Kim et al, 2022 (MCS, Women) 1.01 [0.88, 1.16] 6.29
Kim et al, 2023 (HPFS) 1.22 [0.89, 1.67] 4.12
Kim et al, 2023 (NHS) 1.26 [0.96, 1.66] 4.58
Kim et al, 2023 (NHSII) 1.35 [0.81, 2.25] 2.43
Thompson et al, 2023 1.05 [0.86, 1.28] 5.57
Heterogeneity: τ^2 = 0.00, I^2 = 0.00%, H^2 = 1.00 1.08 [1.00, 1.16]
Test of $\theta_i = \theta_j$: Q(5) = 3.51, p = 0.62
Liver cancer
Kim et al, 2023 (HPFS) 0.55 [0.15, 2.04] 0.51
Kim et al, 2023 (MCS) 1.13 [0.87, 1.45] 4.82
Kim et al, 2023 (NHS) 2.23 [0.62, 7.96] 0.54
Kim et al, 2023 (NHSII) 0.50 [0.04, 6.41] 0.14
Heterogeneity: τ^2 = 0.00, I^2 = 0.00%, H^2 = 1.00 1.12 [0.88, 1.43]
Test of $\theta_i = \theta_j$: Q(3) = 2.63, p = 0.45
Pancreatic cancer
Kim et al, 2023 (HPFS) 0.84 [0.48, 1.49] 2.09
Kim et al, 2023 (NHS) 0.84 [0.51, 1.40] 2.43
Kim et al, 2023 (NHSII) 0.46 [0.13, 1.64] 0.54
Zhong et al, 2023 1.38 [1.03, 1.85] 4.35
Heterogeneity: τ^2 = 0.07, I^2 = 49.38%, H^2 = 1.98 0.98 [0.67, 1.44]
Test of $\theta_i = \theta_j$: Q(3) = 5.93, p = 0.12
Prostate cancer
Loeb et al, 2022 (HPFS, <65y) 1.03 [0.88, 1.21] 6.06
Loeb et al, 2022 (HPFS, ≥65y) 0.95 [0.86, 1.05] 6.69
Thompson et al, 2023 1.05 [0.93, 1.18] 6.50
Heterogeneity: τ^2 = 0.00, I^2 = 0.00%, H^2 = 1.00 1.00 [0.93, 1.07]
Test of $\theta_i = \theta_j$: Q(2) = 1.83, p = 0.40
Stomach cancer
Kim et al, 2023 (HPFS) 0.75 [0.29, 1.95] 0.90
Kim et al, 2023 (NHS) 0.93 [0.34, 2.53] 0.84
Kim et al, 2023 (NHSII) 6.11 [4.29, 8.70] 3.70
Heterogeneity: τ^2 = 1.73, I^2 = 92.12%, H^2 = 12.70 1.71 [0.36, 8.12]
Test of $\theta_i = \theta_j$: Q(2) = 25.39, p = 0.00
0.25
1.002.00

(D) Mortality and subtypes

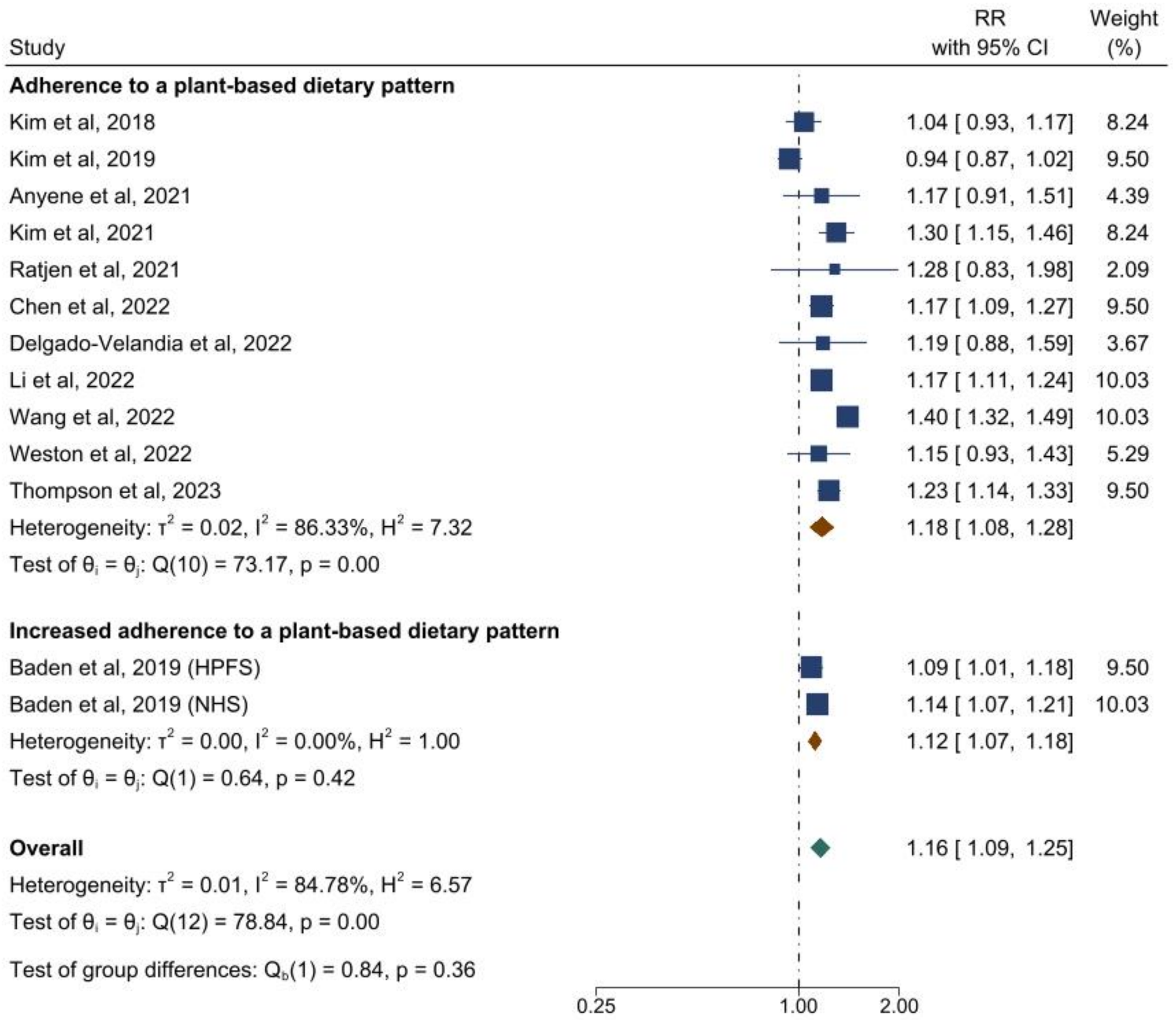

CVD mortality

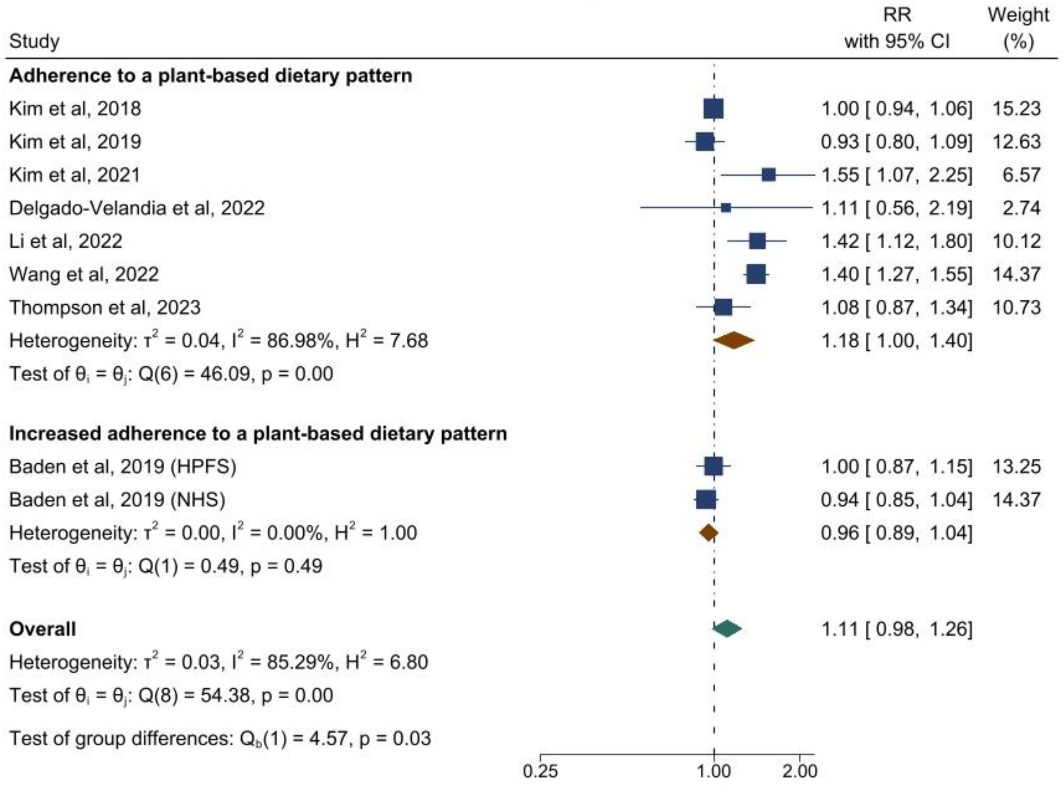

Cancer mortality

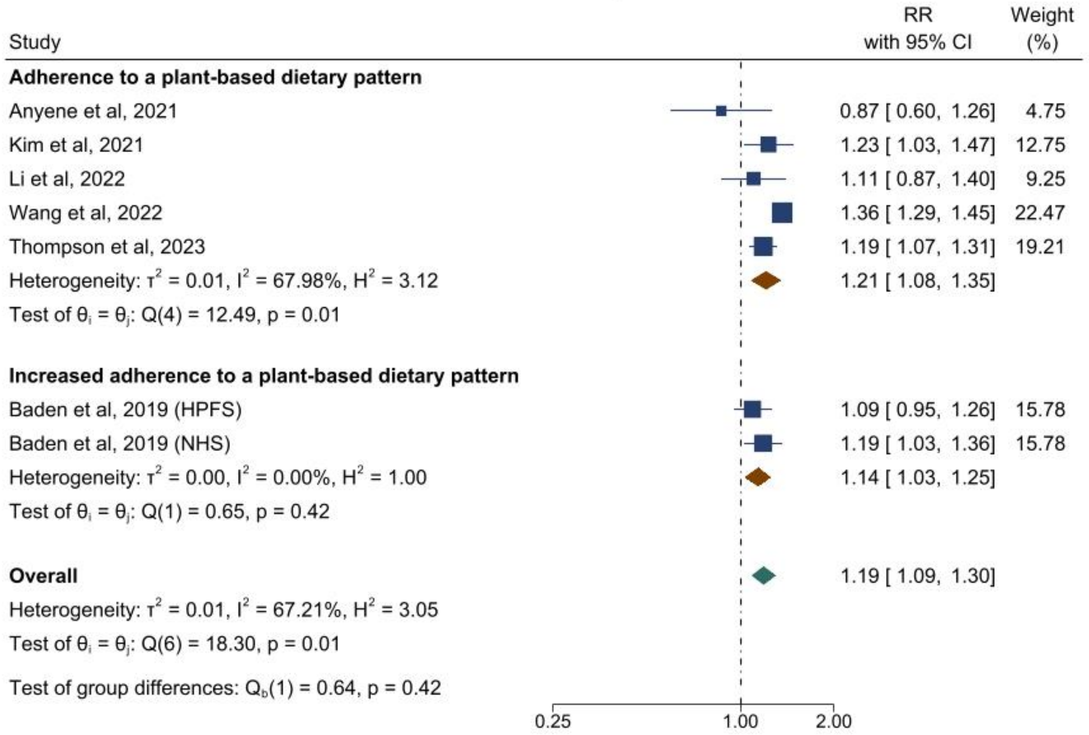

Abbreviations: HPFS, Health Professionals Follow-up Study; NHS, Nurses' Health Study; NHSII, Nurses' Health Study II; MCS, Multiethnic Cohort Study; CVD, cardiovascular disease; MI, myocardial infarction.

Supplemental Figure S11. Funnel Plot of Prospective Studies Examining the Association Between Plant-Based Dietary Patterns and Risks of Incident Type 2 Diabetes, Cardiovascular Disease, Cancer, and Mortality using Random-Effects Meta-Analysis.

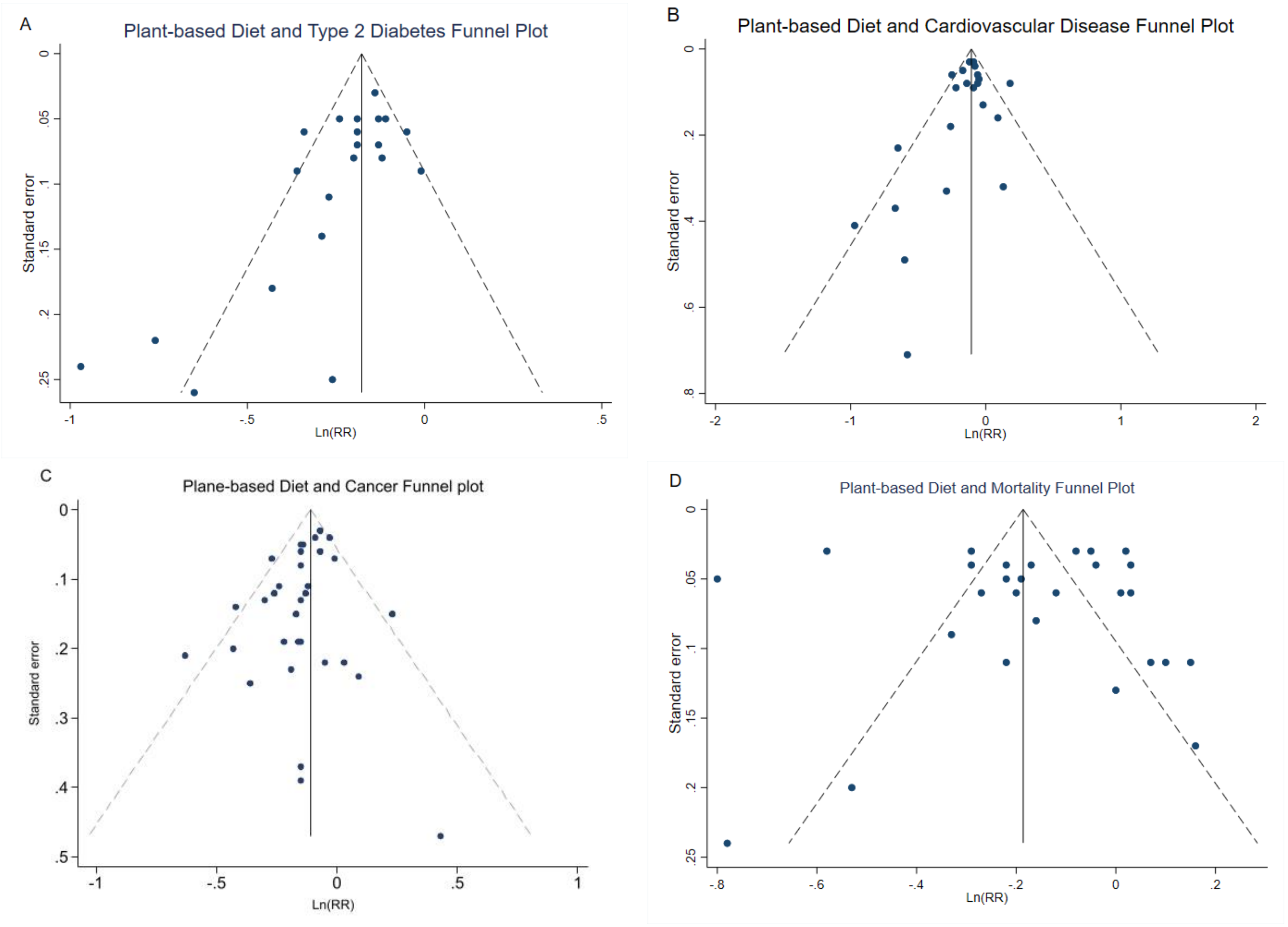

Supplemental Figure S12. Trim-and-fill Analysis to Account for Potential Publication Bias using Random-Effects Meta-Analysis.

Legend: Funnel plot was updated with additional studies (in orange circles) that was filled in by the *trimfill* module in STATA.

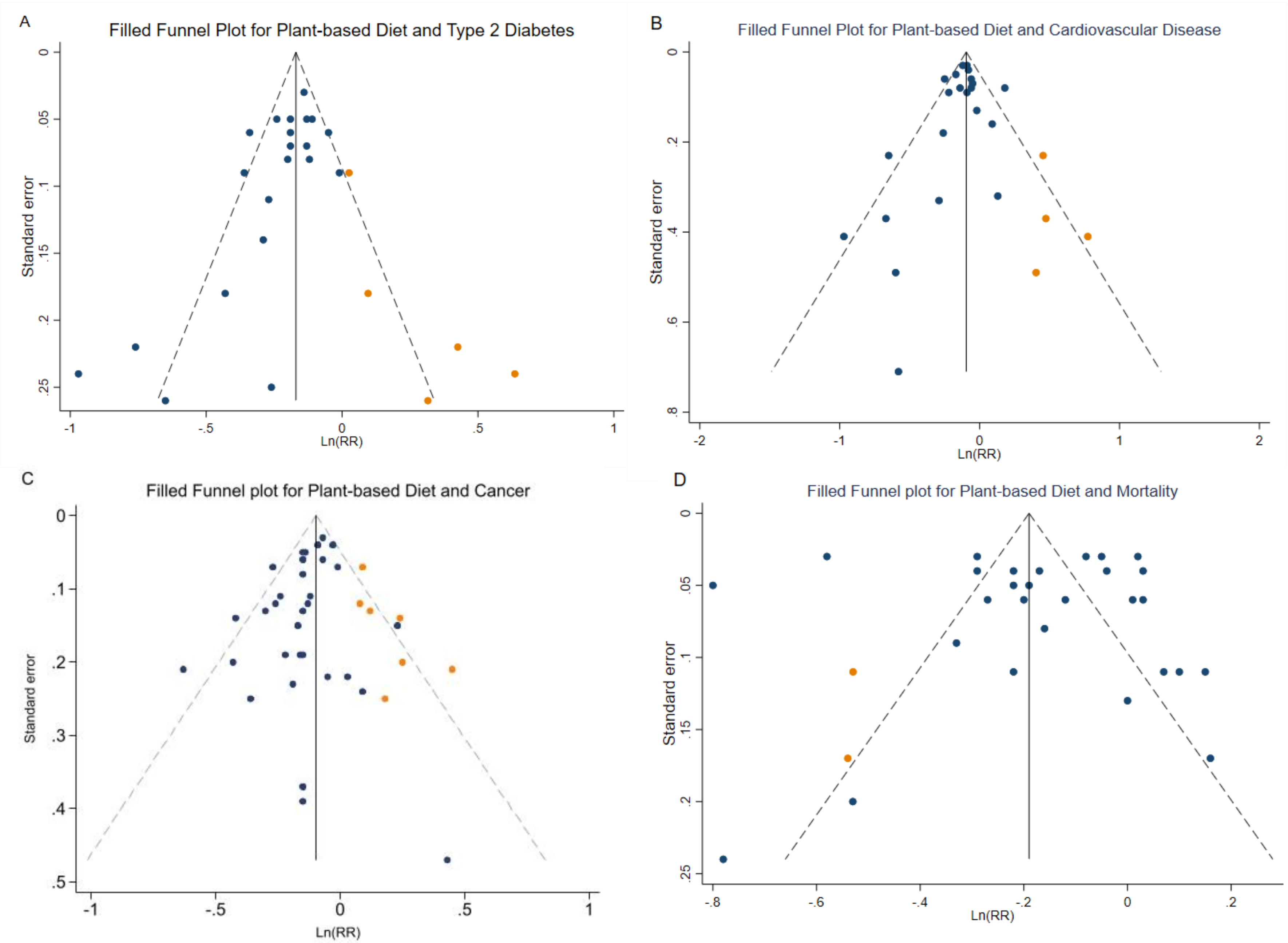

REFERENCES

1. Vang A, Singh PN, Lee JW, Haddad EH, Brinegar CH. Meats, processed meats, obesity, weight gain and occurrence of diabetes among adults: findings from Adventist Health Studies. Annals of nutrition & metabolism. 2008/03/20 ed. 2008;52(2):96–104.
2. Tonstad S, Stewart K, Oda K, Batech M, Herring RP, Fraser GE. Vegetarian diets and incidence of diabetes in the Adventist Health Study-2. Nutr Metab Cardiovasc Dis. 2011/10/11 ed. 2013 Apr;23(4):292–9.
3. Satija A, Bhupathiraju SN, Rimm EB, Spiegelman D, Chiuve SE, Borgi L, et al. Plant-Based Dietary Patterns and Incidence of Type 2 Diabetes in US Men and Women: Results from Three Prospective Cohort Studies. PLoS medicine. 2016/06/15 ed. 2016 Jun;13(6):e1002039.
4. Chen Z, Zuurmond MG, van der Schaft N, Nano J, Wijnhoven HAH, Ikram MA, et al. Plant versus animal based diets and insulin resistance, prediabetes and type 2 diabetes: the Rotterdam Study. Eur J Epidemiol. 2018/06/28 ed. 2018 Sep;33(9):883–93.
5. Chen GC, Koh WP, Neelakantan N, Yuan JM, Qin LQ, van Dam RM. Diet Quality Indices and Risk of Type 2 Diabetes Mellitus: The Singapore Chinese Health Study. Am J Epidemiol. 2018;187(12):2651–61.
6. Chiu THT, Pan WH, Lin MN, Lin CL. Vegetarian diet, change in dietary patterns, and diabetes risk: a prospective study. Nutr Diabetes. 2018/03/20 ed. 2018 Mar 9;8(1):12.
7. Papier K, Appleby PN, Fensom GK, Knuppel A, Perez-Cornago A, Schmidt JA, et al. Vegetarian diets and risk of hospitalisation or death with diabetes in British adults: results from the EPIC-Oxford study. Nutr Diabetes. 2019/02/26 ed. 2019 Feb 25;9(1):7.
8. Choi Y, Larson N, Gallaher DD, Odegaard AO, Rana JS, Shikany JM, et al. A Shift Toward a Plant-Centered Diet From Young to Middle Adulthood and Subsequent Risk of Type 2 Diabetes and Weight Gain: The Coronary Artery Risk Development in Young Adults (CARDIA) Study. Diabetes Care. 2020/08/28 ed. 2020 Nov;43(11):2796–803.
9. Chen Z, Drouin-Chartier JP, Li Y, Baden MY, Manson JE, Willett WC, et al. Changes in Plant-Based Diet Indices and Subsequent Risk of Type 2 Diabetes in Women and Men: Three U.S. Prospective Cohorts. Diabetes Care. 2021/01/15 ed. 2021 Mar;44(3):663–71.
10. Flores AC, Heron C, Kim JI, Martin B, Al-Shaar L, Tucker KL, et al. Prospective Study of Plant-Based Dietary Patterns and Diabetes in Puerto Rican Adults. J Nutr. 2021 Sep 13;151(12):3795–800.
11. Laouali N, Shah S, MacDonald CJ, Mahamat-Saleh Y, El Fatouhi D, Mancini F, et al. BMI in the Associations of Plant-Based Diets with Type 2 Diabetes and Hypertension Risks in Women: The E3N Prospective Cohort Study. J Nutr. 2021/07/09 ed. 2021 Sep 4;151(9):2731–40.
12. Yang X, Li Y, Wang C, Mao Z, Chen Y, Ren P, et al. Association of plant-based diet and type 2 diabetes mellitus in Chinese rural adults: The Henan Rural Cohort Study. J Diabetes Investig. 2021/02/10 ed. 2021 Sep;12(9):1569–76.
13. Bhupathiraju SN, Sawicki CM, Goon S, Gujral UP, Hu FB, Kandula NR, et al. A healthy plant–based diet is favorably associated with cardiometabolic risk factors among participants of South Asian ancestry. Am J Clin Nutr. 2022 Jun 22;116(4):1078–90.
14. Chen B, Zeng J, Qin M, Xu W, Zhang Z, Li X, et al. The Association Between Plant-Based Diet Indices and Obesity and Metabolic Diseases in Chinese Adults: Longitudinal Analyses From the China Health and Nutrition Survey. Front Nutr. 2022 Jun 20;9:881901.
15. Kim J, Giovannucci E. Healthful Plant-Based Diet and Incidence of Type 2 Diabetes in Asian Population. Nutrients. 2022 Jul 27;14(15):3078.
16. Glenn AJ, Li J, Lo K, Jenkins DJA, Boucher BA, Hanley AJ, et al. The Portfolio Diet and Incident Type 2 Diabetes: Findings From the Women's Health Initiative Prospective Cohort Study. Diabetes Care. 2022 Sep 26;46(1):28–37.
17. Zhang S, Stubbendorff A, Olsson K, Ericson U, Niu K, Qi L, et al. Adherence to the EAT-Lancet diet, genetic susceptibility, and risk of type 2 diabetes in Swedish adults. Metabolism - Clinical and

Experimental [Internet]. 2023 Apr 1 [cited 2023 Jul 29];141. Available from: https://www.metabolismjournal.com/article/S0026-0495(23)00004-5/fulltext
18. Satija A, Bhupathiraju SN, Spiegelman D, Chiuve SE, Manson JE, Willett W, et al. Healthful and Unhealthful Plant-Based Diets and the Risk of Coronary Heart Disease in U.S. Adults. J Am Coll Cardiol. 2017 Jul 25;70(4):411–22.
19. Kim H, Caulfield LE, Garcia-Larsen V, Steffen LM, Coresh J, Rebholz CM. Plant-Based Diets Are Associated With a Lower Risk of Incident Cardiovascular Disease, Cardiovascular Disease Mortality, and All-Cause Mortality in a General Population of Middle-Aged Adults. J Am Heart Assoc. 2019/08/08 ed. 2019 Aug 20;8(16):e012865.
20. Tong TYN, Appleby PN, Bradbury KE, Perez-Cornago A, Travis RC, Clarke R, et al. Risks of ischaemic heart disease and stroke in meat eaters, fish eaters, and vegetarians over 18 years of follow-up: results from the prospective EPIC-Oxford study. BMJ. 2019;366:l4897.
21. Chiu THT, Chang HR, Wang LY, Chang CC, Lin MN, Lin CL. Vegetarian diet and incidence of total, ischemic, and hemorrhagic stroke in 2 cohorts in Taiwan. Neurology. 2020/02/28 ed. 2020 Mar 17;94(11):e1112–21.
22. Shan Z, Li Y, Baden MY, Bhupathiraju SN, Wang DD, Sun Q, et al. Association Between Healthy Eating Patterns and Risk of Cardiovascular Disease. JAMA Intern Med. 2020/06/17 ed. 2020 Aug 1;180(8):1090–100.
23. Baden MY, Shan Z, Wang F, Li Y, Manson JE, Rimm EB, et al. Quality of Plant-Based Diet and Risk of Total, Ischemic, and Hemorrhagic Stroke. Neurology. 2021;96(15):e1940–53.
24. Glenn AJ, Lo K, Jenkins DJA, Boucher BA, Hanley AJ, Kendall CWC, et al. Relationship Between a Plant-Based Dietary Portfolio and Risk of Cardiovascular Disease: Findings From the Women’s Health Initiative Prospective Cohort Study. J Am Heart Assoc. 2021/08/05 ed. 2021 Aug 17;10(16):e021515.
25. Petermann-Rocha F, Parra-Soto S, Gray S, Anderson J, Welsh P, Gill J, et al. Vegetarians, fish, poultry, and meat-eaters: who has higher risk of cardiovascular disease incidence and mortality? A prospective study from UK Biobank. Eur Heart J. 2020/12/15 ed. 2021 Mar 21;42(12):1136–43.
26. Chen YY, Chen GC, Abittan N, Xing J, Mossavar-Rahmani Y, Sotres-Alvarez D, et al. Healthy dietary patterns and risk of cardiovascular disease in US Hispanics/Latinos: the Hispanic Community Health Study/Study of Latinos (HCHS/SOL). The American Journal of Clinical Nutrition. 2022 Oct 1;116(4):920–7.
27. Choi Y, Gallaher DD, Svendsen K, Meyer KA, Steffen LM, Schreiner PJ, et al. Simple Nutrient-Based Rules vs. a Nutritionally Rich Plant-Centered Diet in Prediction of Future Coronary Heart Disease and Stroke: Prospective Observational Study in the US. Nutrients. 2022 Jan 21;14(3):469.
28. Ibsen DB, Christiansen AH, Olsen A, Tjønneland A, Overvad K, Wolk A, et al. Adherence to the EAT-Lancet Diet and Risk of Stroke and Stroke Subtypes: A Cohort Study. Stroke. 2022 Jan;53(1):154–63.
29. Kouvari M, Tsiampalis T, Chrysohoou C, Georgousopoulou E, Skoumas J, Mantzoros CS, et al. Quality of plant-based diets in relation to 10-year cardiovascular disease risk: the ATTICA cohort study. Eur J Nutr. 2022 Aug 1;61(5):2639–49.
30. Lazarova SV, Sutherland JM, Jessri M. Adherence to emerging plant-based dietary patterns and its association with cardiovascular disease risk in a nationally representative sample of Canadian adults. Am J Clin Nutr. 2022 Mar 10;116(1):57–73.
31. Zhang S, Dukuzimana J, Stubbendorff A, Ericson U, Borné Y, Sonestedt E. Adherence to the EAT-Lancet diet and risk of coronary events in the Malmö Diet and Cancer cohort study. The American Journal of Clinical Nutrition. 2023 May 1;117(5):903–9.
32. Thompson AS, Tresserra-Rimbau A, Karavasiloglou N, Jennings A, Cantwell M, Hill C, et al. Association of Healthful Plant-based Diet Adherence With Risk of Mortality and Major Chronic Diseases Among Adults in the UK. JAMA Network Open. 2023 Mar 28;6(3):e234714.

33. Weston LJ, Kim H, Talegawkar SA, Tucker KL, Correa A, Rebholz CM. Plant-based diets and incident cardiovascular disease and all-cause mortality in African Americans: A cohort study. PLoS Med. 2022 Jan 5;19(1):e1003863.
34. Berkel J, de Waard F. Mortality pattern and life expectancy of Seventh-Day Adventists in the Netherlands. Int J Epidemiol. 1983/12/01 ed. 1983 Dec;12(4):455–9.
35. Ogata M, Ikeda M, Kuratsune M. Mortality among Japanese Zen priests. J Epidemiol Community Health. 1984/06/01 ed. 1984 Jun;38(2):161–6.
36. Thorogood M, Mann J, Appleby P, McPherson K. Risk of death from cancer and ischaemic heart disease in meat and non-meat eaters. Bmj. 1994/06/25 ed. 1994 Jun 25;308(6945):1667–70.
37. Key TJ, Thorogood M, Appleby PN, Burr ML. Dietary habits and mortality in 11,000 vegetarians and health conscious people: results of a 17 year follow up. Bmj. 1996/09/28 ed. 1996 Sep 28;313(7060):775–9.
38. Key TJ, Fraser GE, Thorogood M, Appleby PN, Beral V, Reeves G, et al. Mortality in vegetarians and nonvegetarians: detailed findings from a collaborative analysis of 5 prospective studies. Am J Clin Nutr. 1999/09/09 ed. 1999 Sep;70(3 Suppl):516s–24s.
39. Appleby PN, Key TJ, Thorogood M, Burr ML, Mann J. Mortality in British vegetarians. Public Health Nutr. 2002 Feb;5(1):29–36.
40. Chang-Claude J, Hermann S, Eilber U, Steindorf K. Lifestyle determinants and mortality in German vegetarians and health-conscious persons: results of a 21-year follow-up. Cancer Epidemiol Biomarkers Prev. 2005/04/13 ed. 2005 Apr;14(4):963–8.
41. Bamia C, Trichopoulos D, Ferrari P, Overvad K, Bjerregaard L, Tjønneland A, et al. Dietary patterns and survival of older Europeans: the EPIC-Elderly Study (European Prospective Investigation into Cancer and Nutrition). Public Health Nutr. 2007/03/27 ed. 2007 Jun;10(6):590–8.
42. Key TJ, Appleby PN, Spencer EA, Travis RC, Roddam AW, Allen NE. Mortality in British vegetarians: results from the European Prospective Investigation into Cancer and Nutrition (EPIC-Oxford). Am J Clin Nutr. 2009/03/20 ed. 2009 May;89(5):1613s–9s.
43. Orlich MJ, Singh PN, Sabaté J, Jaceldo-Siegl K, Fan J, Knutsen S, et al. Vegetarian dietary patterns and mortality in Adventist Health Study 2. JAMA Intern Med. 2013/07/10 ed. 2013 Jul 8;173(13):1230–8.
44. Martínez-González MA, Sánchez-Tainta A, Corella D, Salas-Salvadó J, Ros E, Arós F, et al. A provegetarian food pattern and reduction in total mortality in the Prevención con Dieta Mediterránea (PREDIMED) study. Am J Clin Nutr. 2014/05/30 ed. 2014 Jul;100 Suppl 1:320s–8s.
45. Mihrshahi S, Ding D, Gale J, Allman-Farinelli M, Banks E, Bauman AE. Vegetarian diet and all-cause mortality: Evidence from a large population-based Australian cohort - the 45 and Up Study. Prev Med. 2017/01/04 ed. 2017 Apr;97:1–7.
46. Kim H, Caulfield LE, Rebholz CM. Healthy Plant-Based Diets Are Associated with Lower Risk of All-Cause Mortality in US Adults. J Nutr. 2018/04/17 ed. 2018 Apr 1;148(4):624–31.
47. Baden MY, Liu G, Satija A, Li Y, Sun Q, Fung TT, et al. Changes in Plant-Based Diet Quality and Total and Cause-Specific Mortality. Circulation. 2019;140(12):979–91.
48. Anyene IC, Ergas IJ, Kwan ML, Roh JM, Ambrosone CB, Kushi LH, et al. Plant-Based Dietary Patterns and Breast Cancer Recurrence and Survival in the Pathways Study. Nutrients. 2021/10/24 ed. 2021 Sep 25;13(10).
49. Kim J, Kim H, Giovannucci EL. Plant-based diet quality and the risk of total and disease-specific mortality: A population-based prospective study. Clinical Nutrition. 2021 Dec 1;40(12):5718–25.
50. Lo K, Glenn AJ, Yeung S, Kendall CWC, Sievenpiper JL, Jenkins DJA, et al. Prospective Association of the Portfolio Diet with All-Cause and Cause-Specific Mortality Risk in the Mr. OS and Ms. OS Study. Nutrients. 2021 Dec 3;13(12):4360.
51. Ratjen I, Enderle J, Burmeister G, Koch M, Nöthlings U, Hampe J, et al. Post-diagnostic reliance on plant-compared with animal-based foods and all-cause mortality in omnivorous long-term colorectal cancer survivors. The American Journal of Clinical Nutrition. 2021;114(2):441–9.

52. Chen H, Shen J, Xuan J, Zhu A, Ji JS, Liu X, et al. Plant-based dietary patterns in relation to mortality among older adults in China. Nat Aging. 2022 Mar;2(3):224–30.
53. Delgado-Velandia M, Maroto-Rodríguez J, Ortolá R, García-Esquinas E, Rodríguez-Artalejo F, Sotos-Prieto M. Plant-Based Diets and All-cause and Cardiovascular Mortality in a Nationwide Cohort in Spain: The ENRICA Study. Mayo Clinic Proceedings. 2022 Nov 1;97(11):2005–15.
54. Li H, Zeng X, Wang Y, Zhang Z, Zhu Y, Li X, et al. A prospective study of healthful and unhealthful plant-based diet and risk of overall and cause-specific mortality. Eur J Nutr. 2021/08/12 ed. 2022 Feb;61(1):387–98.
55. Wang DD, Li Y, Nguyen XMT, Song RJ, Ho YL, Hu FB, et al. Degree of adherence to plant-based diet and total and cause-specific mortality: prospective cohort study in the Million Veteran Program. Public Health Nutrition. 2023 Feb;26(2):381–92.
56. Shan Z, Wang F, Li Y, Baden MY, Bhupathiraju SN, Wang DD, et al. Healthy Eating Patterns and Risk of Total and Cause-Specific Mortality. JAMA Internal Medicine. 2023 Feb 1;183(2):142–53.
57. Fraser GE. Associations between diet and cancer, ischemic heart disease, and all-cause mortality in non-Hispanic white California Seventh-day Adventists. Am J Clin Nutr. 1999/09/09 ed. 1999 Sep;70(3 Suppl):532s–8s.
58. Key TJ, Appleby PN, Spencer EA, Travis RC, Roddam AW, Allen NE. Cancer incidence in vegetarians: results from the European Prospective Investigation into Cancer and Nutrition (EPIC-Oxford). Am J Clin Nutr. 2009/03/13 ed. 2009 May;89(5):1620s–6s.
59. Cade JE, Taylor EF, Burley VJ, Greenwood DC. Common dietary patterns and risk of breast cancer: analysis from the United Kingdom Women’s Cohort Study. Nutr Cancer. 2010/04/02 ed. 2010;62(3):300–6.
60. Tantamango-Bartley Y, Jaceldo-Siegl K, Fan J, Fraser G. Vegetarian diets and the incidence of cancer in a low-risk population. Cancer Epidemiol Biomarkers Prev. 2012/11/22 ed. 2013 Feb;22(2):286–94.
61. Gilsing AM, Schouten LJ, Goldbohm RA, Dagnelie PC, van den Brandt PA, Weijenberg MP. Vegetarianism, low meat consumption and the risk of colorectal cancer in a population based cohort study. Sci Rep. 2015/09/01 ed. 2015 Aug 28;5:13484.
62. Orlich MJ, Singh PN, Sabaté J, Fan J, Sveen L, Bennett H, et al. Vegetarian dietary patterns and the risk of colorectal cancers. JAMA Intern Med. 2015/03/10 ed. 2015 May;175(5):767–76.
63. Gilsing AM, Weijenberg MP, Goldbohm RA, Dagnelie PC, van den Brandt PA, Schouten LJ. Vegetarianism, low meat consumption and the risk of lung, postmenopausal breast and prostate cancer in a population-based cohort study. Eur J Clin Nutr. 2016/03/05 ed. 2016 Jun;70(6):723–9.
64. Tantamango-Bartley Y, Knutsen SF, Knutsen R, Jacobsen BK, Fan J, Beeson WL, et al. Are strict vegetarians protected against prostate cancer? Am J Clin Nutr. 2015/11/13 ed. 2016 Jan;103(1):153–60.
65. Penniecook-Sawyers JA, Jaceldo-Siegl K, Fan J, Beeson L, Knutsen S, Herring P, et al. Vegetarian dietary patterns and the risk of breast cancer in a low-risk population. Br J Nutr. 2016/03/19 ed. 2016 May 28;115(10):1790–7.
66. Rada-Fernandez de Jauregui D, Evans CEL, Jones P, Greenwood DC, Hancock N, Cade JE. Common dietary patterns and risk of cancers of the colon and rectum: Analysis from the United Kingdom Women’s Cohort Study (UKWCS). Int J Cancer. 2018/03/09 ed. 2018 Aug 15;143(4):773–81.
67. Kane-Diallo A, Srour B, Sellem L, Deschasaux M, Latino-Martel P, Hercberg S, et al. Association between a pro plant-based dietary score and cancer risk in the prospective NutriNet-santé cohort. International Journal of Cancer. 2018;143(9):2168–76.
68. Leone A, Martínez-González M, Martin-Gorgojo A, Sánchez-Bayona R, De Amicis R, Bertoli S, et al. Mediterranean diet, Dietary Approaches to Stop Hypertension, and Pro-vegetarian dietary pattern in relation to the risk of basal cell carcinoma: a nested case-control study within the Seguimiento Universidad de Navarra (SUN) cohort. Am J Clin Nutr. 2020/06/04 ed. 2020 Aug 1;112(2):364–72.

69. Romanos-Nanclares A, Toledo E, Sánchez-Bayona R, Sánchez-Quesada C, Martínez-González M, Gea A. Healthful and unhealthful provegetarian food patterns and the incidence of breast cancer: Results from a Mediterranean cohort. Nutrition. 2020/08/01 ed. 2020 Nov;79–80:110884.
70. Romanos-Nanclares A, Willett WC, Rosner BA, Collins LC, Hu FB, Toledo E, et al. Healthful and Unhealthful Plant-Based Diets and Risk of Breast Cancer in U.S. Women: Results from the Nurses' Health Studies. Cancer Epidemiology, Biomarkers & Prevention. 2021;30(10):1921–31.
71. Kim J, Boushey CJ, Wilkens LR, Haiman CA, Le Marchand L, Park SY. Plant-based dietary patterns defined by a priori indices and colorectal cancer risk by sex and race/ethnicity: the Multiethnic Cohort Study. BMC Medicine. 2022 Nov 29;20(1):430.
72. Loeb S, Fu BC, Bauer SR, Pernar CH, Chan JM, Van Blarigan EL, et al. Association of plant-based diet index with prostate cancer risk. Am J Clin Nutr. 2021 Nov 13;115(3):662–70.
73. Kim J, Setiawan VW, Wilkens LR, Le Marchand L, Park SY. Healthful Plant-Based Dietary Pattern and Risk of Hepatocellular Carcinoma in a Multiethnic Population: A Cohort Study. The American Journal of Clinical Nutrition. 2023 Jul 1;118(1):194–200.
74. Kim J, Khil J, Kim H, Keum N, Zhang X, Giovannucci E. Plant-based dietary patterns and the risk of digestive system cancers in 3 large prospective cohort studies. Eur J Epidemiol. 2023 Jun 1;38(6):617–27.
75. Shah S, Mahamat-Saleh Y, Ait-Hadad W, Koemel NA, Varraso R, Boutron-Ruault MC, et al. Long-term adherence to healthful and unhealthful plant-based diets and breast cancer risk overall and by hormone receptor and histologic subtypes among postmenopausal females. The American Journal of Clinical Nutrition. 2023 Mar 1;117(3):467–76.
76. Zhong GC, Li Z, You AJ, Zhu Q, Wang CR, Yang PF. Plant-based diets and the risk of pancreatic cancer: a large prospective multicenter study. The American Journal of Clinical Nutrition. 2023 Feb 1;117(2):235–42.
